# Non-random transmission of parental alleles into crop-wild and crop-weed hybrid lineages separated by a transgene and neutral identifiers in rice

**DOI:** 10.1038/s41598-017-10596-4

**Published:** 2017-09-05

**Authors:** Zhe Wang, Lei Wang, Zhi Wang, Bao-Rong Lu

**Affiliations:** Ministry of Education Key Laboratory for biodiversity science and Ecological Engineering, Department of Ecology and Evolutionary Biology, Fudan University, Shanghai, 200433 China

## Abstract

It is essential to assess environmental impact of transgene flow from genetically engineered crops to their wild or weedy relatives before commercialization. Measuring comparative trials of fitness in the transgene-flow-resulted hybrids plays the key role in the assessment, where the segregated isogenic hybrid lineages/subpopulations with or without a transgene of the same genomic background are involved. Here, we report substantial genomic differentiation between transgene-present and -absent lineages (F_2_-F_3_) divided by a glyphosate-resistance transgene from a crop-wild/weed hybrid population in rice. We further confirmed that such differentiation is attributed to increased frequencies of crop-parent alleles in transgenic hybrid lineages at multiple loci across the genome, as estimated by SSR (simple sequence repeat) markers. Such preferential transmission of parental alleles was also found in equally divided crop-wild/weed hybrid lineages with or without a particular neutral SSR identifier. We conclude that selecting either a transgene or neutral marker as an identifier to create hybrid lineages will result in different genomic background of the lineages due to non-random transmission of parental alleles. Non-random allele transmission may misrepresent the outcomes of fitness effects. We therefore propose seeking other means to evaluate fitness effects of transgenes for assessing environmental impact caused by crop-to-wild/weed gene flow.

## Introduction

The undesired environmental impact caused by transgene flow from genetically engineered (GE) crops to their wild and weedy relatives has stimulated great biosafety concerns worldwide^1–6^. It is necessary then to assess the environmental impact prior to the commercialization of any GE crops. The common practice to assess such impact includes two key components: determining frequencies of (trans)gene flow and estimating fitness effects of a transgene acquired by wild/weedy relative populations^5–7^. Concerns centre on the possibility that transgenes with natural selective advantages or disadvantages may convey fitness benefits or costs to wild/weedy populations^6, 8, 9^, creating unwanted environmental and evolutionary consequences^3, 7, 10–12^. Transgenic fitness has been extensively estimated in many types of hybrid descendants derived from crosses between GE crop species and their wild relatives to predict the potential environmental impact of transgene flow, including oilseed rape^13, 14^, squashes^1, 15^, sunflowers^10, 16^, maize^17^, and rice^18–20^. Therefore, fitness becomes essential for assessing the environmental impact caused by transgene flow, after the frequency of gene flow is determined^5^.

**Figure 1:**
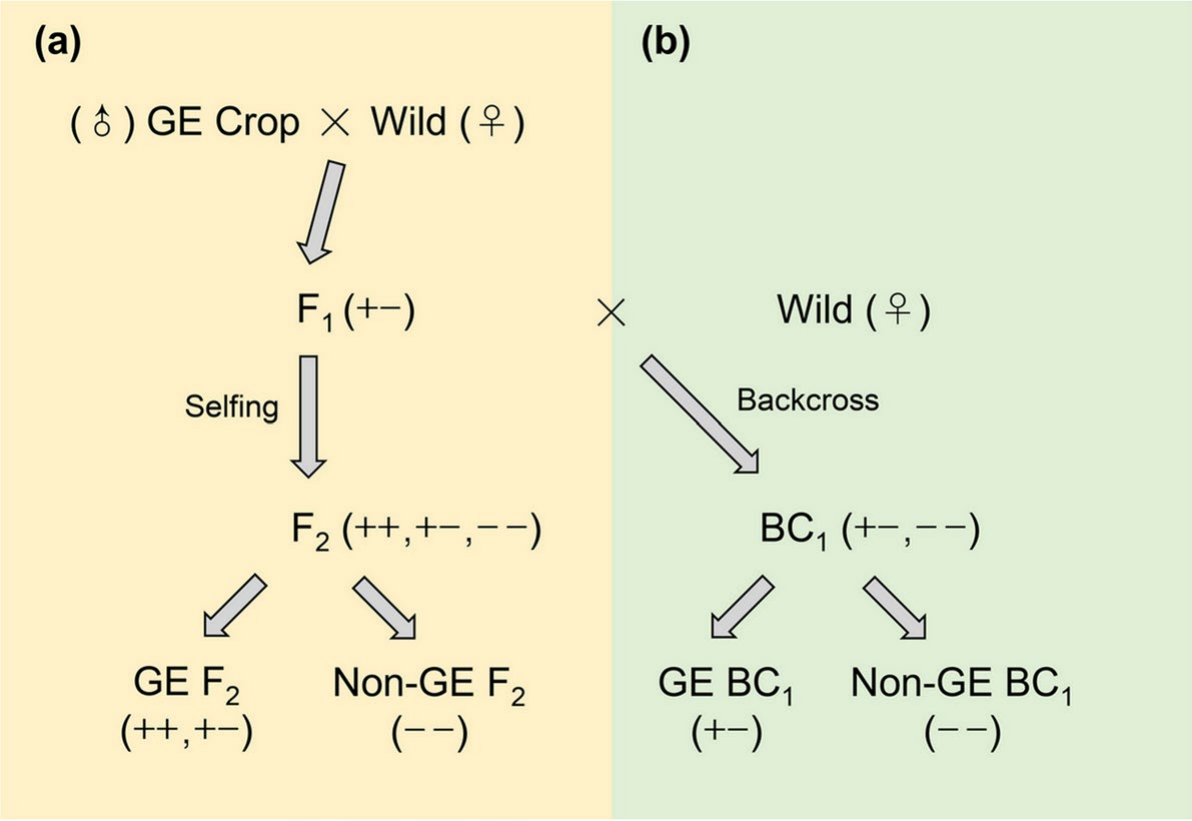
A schematic pedigree to illustrate the production of F_2_ (**a**) and BC_1_ (**b**) crop-wild or crop-weed hybrid lineages for estimating the fitness effect of a transgene. “GE crop” indicates a genetically engineered crop parent; “Wild” indicates a wild or weedy parent; “++ and +−” indicate transgene-homozygous and -hemizygous GE lineages, and “− −” indicates non-GE lineages.

Usually, transgenic fitness is estimated in a common-garden experiment, where artificially produced ISOGENIC hybrid lineages (or subpopulations) with or without a transgene from a crop-wild/weed hybrid population (F_2_ or BC_1_) are included for comparison^10, 17–22^. Our literature survey from the Web of Science (http://apps.webofknowledge.com/) indicated that more than 70% of the recently published relevant research articles (2000–2017) included the isogenic hybrid lineages to estimate transgenic fitness (Supplementary Fig. 1). Therefore, simulating crop-wild or crop-weed transgene flow, hybrids between a GE crop and its wild/weedy relative species are produced and F_2_ or BC_1_ hybrid populations were derived through self-pollination of F_1_ hybrids (Fig. 1a) or backcrosses of F_1_ hybrids with their wild/weedy parents (Fig. 1b). The key point to assess fitness of a transgene is to divide a F_2_ or BC_1_ hybrid population into isogenic lineages/subpopulations with the target transgene (including transgene-homozygous and -heterozygous) or without a transgene, using the transgene as an identifier (Fig. 1a,b). The fitness of a transgene is estimated by comparing fitness-related traits between the transgene-present and -absent lineages in the common-garden experiments^10, 17–22^, under the assumption that transgene-present and -absent lineages only differ on average in the presence of the transgene. In other words, the genetic composition of the hybrid lineages/subpopulations with or without the transgene are considered to be genetically equivalent and effectively isogenic. Consequently, the detected differences between these lineages are only due to the presence or absence of the transgene(s)^17–22^. However, the assumption has never been properly tested.

**Figure 2:**
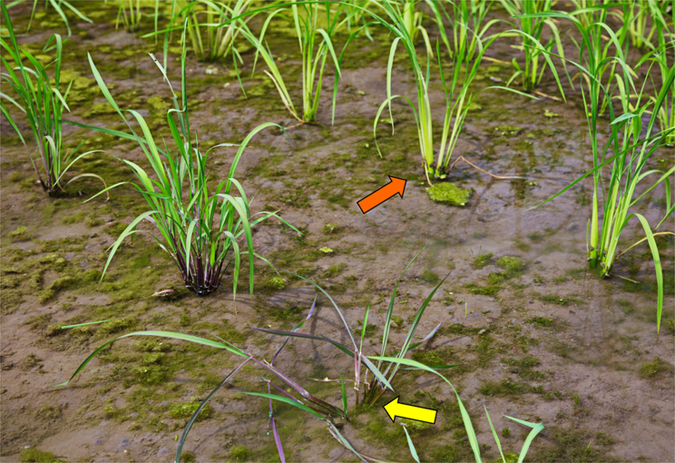
Phenotypic variation of seedlings in F_3_ transgene-present (orange arrow) and transgene-absent (yellow arrow) hybrid lineages derived from an artificial cross between an *epsps* (5-enolpyruvoylshikimate-3-phosphate synthase) transgenic rice line and wild rice (*Oryza rufipogon*).

In our common garden experiment aimed to estimate fitness of an *epsps* (5-enolpyruvoylshikimate-3-phosphate synthase) transgene, we observed phenotypic differences between F_3_ transgenic and non-transgenic seedlings derived from the same crop-wild F_1_ hybrids (Fig. 2). The hybrids included two parents: an herbicide-resistant GE rice (*Oryza sativa*) line and a wild rice (*O. rufipogon*) accession, sharing the AA genome^23, 24^. We further noticed that the transgenic seedlings exhibited more traits from the cultivated parent (e.g., shorter and more erect plants without pigmentation at the base, see Fig. 2), whereas the non-transgenic seedlings showed more traits from the wild parent (e.g., taller and prostrating plants with purple pigmentation at the base, see Fig. 2). Apparently, transgenic lineages inherited more traits from the crop parent, while non-transgenic lineages inherited more traits from the wild parent.

**Figure 3:**
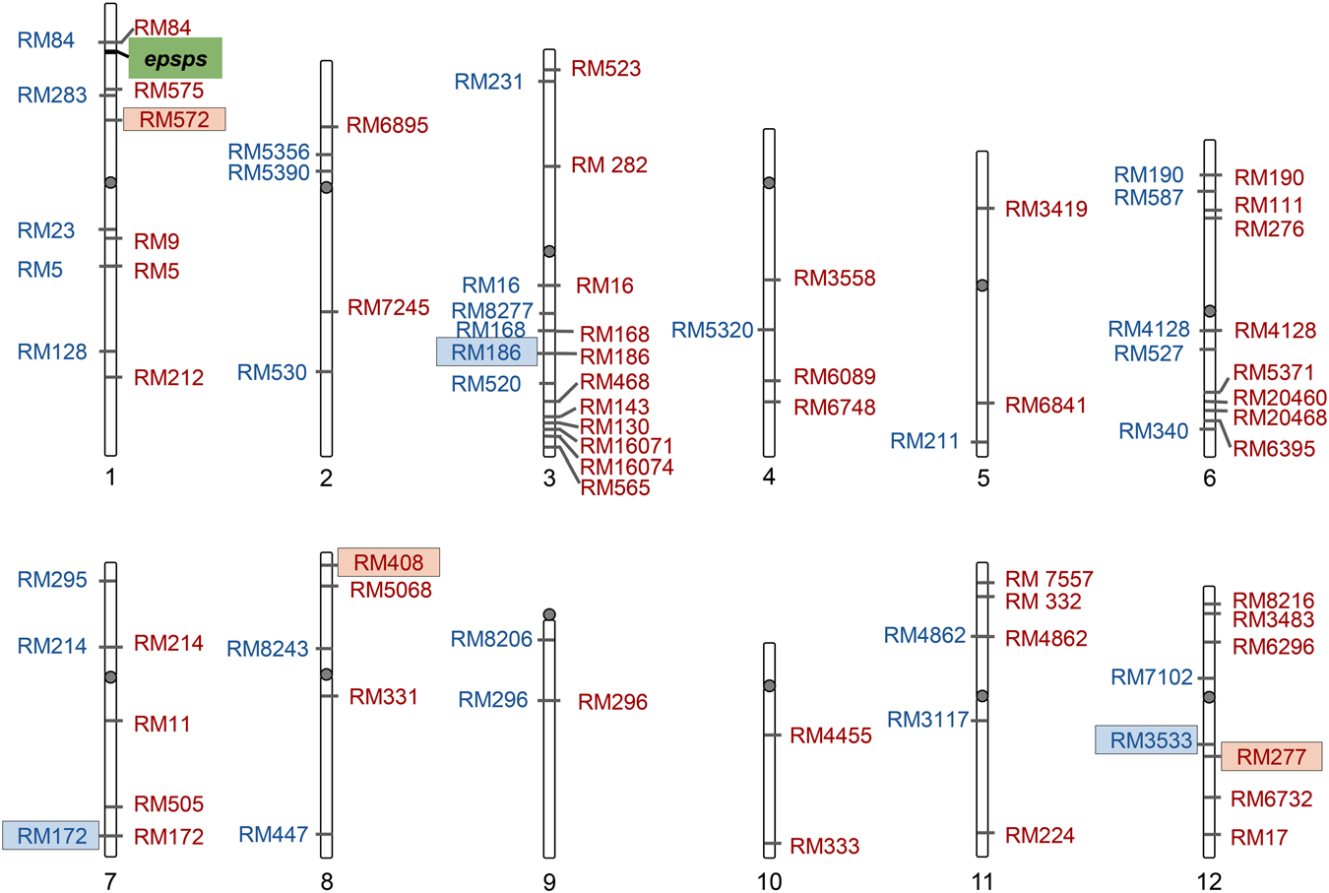
A genomic linkage map illustrating the physical location of the *epsps* (5-enolpyruvoylshikimate-3-phosphate synthase) transgene on chromosome-1 (green box), the six neutral SSR (simple sequence repeat) identifiers (pink boxes for crop-wild and blue boxes for crop-weed hybrids), and 52 (red letters, for crop-wild hybrids) and 32 (blue letters, for crop-weed hybrids) SSR markers located across the 12 rice chromosomes.

The phenotypic differences between transgenic and non-transgenic hybrid seedlings should not be observed under the assumption that the isogenic hybrid lineages only differ in the transgene but share on average the identical genomic background. It is known that genetic hitchhiking shapes the genome of hybrid populations under natural selection^25, 26^. Similarly, can the artificial selection for a specific gene (identifier) distort a part of the genome by hitchhiking towards one of the parents? To address this question, we produced F_2_-F_3_ crop-wild and crop-weed hybrid lineages with or without the *epsps* transgene and neutral microsatellite or SSR (simple sequence repeat) identifiers (Fig. 3) in a new experiment to test the following hypotheses. (1) Dividing hybrid lineages with or without a transgene by selecting transgenic identifier will result in a difference in their genomic background caused by non-random parent-allele transmission; (2) non-random transmission of parental alleles is not necessarily related to selection of the transgene identifier but to any random identifier; and (3) preferential allelic transmission from one parent is associated with the selection of an identifier from the specific parent. The proof of the hypotheses will facilitate our design of a better method to estimate transgenic fitness, which is important for risk assessment of transgene flow from a GE crop to its wild or weedy relative populations. In addition, the generated knowledge can also increase our understanding of the effect of selection on transmission of parental alleles into their hybrid descendants.

## Results

### Differences in the genomic background between transgenic and non-transgenic hybrid lineages

We found considerable differences in the genomic background between the “isogenic” F_2_-F_3_ crop-wild/weed hybrid lineages with or without the *epsps* transgene. The diversity coefficient (*F*_*st*_) analyses indicated a certain level (*F*_*st*_ = 0.02–0.05) of genomic differentiation between transgenic and non-transgenic crop-wild/weed hybrid lineages (Table 1). As a comparison, the *F*_*st*_ values between the corresponding ideal groups were all less than 0.001 for both crop-wild/weed hybrid populations (Table 1), suggesting extremely low genomic differentiation. These results indicated that the division of transgenic and non-transgenic hybrid lineages using the transgene as an identifier would result in differences in their genomic background, probably due to the non-random transmission of parental alleles.

**Table 1:** Diversity coefficient (*F*_*st*_) values (left) and numbers of non-neutral loci (right) between hybrid lineages with or without the *epsps* transgene (as an identifier, GE), with or without the crop-parent SSR markers (as identifiers, CM), and with or without the wild/weedy-parent SSR markers (WM), created from an F_2_/F_3_ crop-wild (W) or crop-weedy (WD) rice hybrid population.

Hybrid population	GE lineages	CM lineages			WM lineages		
		RM572	RM408	RM277	RM572	RM408	RM277
F_2_-W	0.018/14	0.020/8	0.025/13	0.026/14	0.017/7	0.017/10	0.026/13
F_3_-W	0.013/16	0.026/15	0.024/15	0.030/13	0.026/14	0.024/10	0.027/13
		**RM186**	**RM172**	**RM3533**	**RM186**	**RM172**	**RM3533**
F_2_-WD	0.051/15	0.043/16	0.091/9	0.060/8	0.037/9	0.077/11	0.077/11
F_3_-WD	0.045/11	0.046/9	0.104/12	0.052/16	0.045/5	0.129/11	0.053/9

*F*_*st*_ values between the ideal groups are all <0.001. Numbers of non-neutral loci between the ideal groups are close to zero.

**Figure 4:**
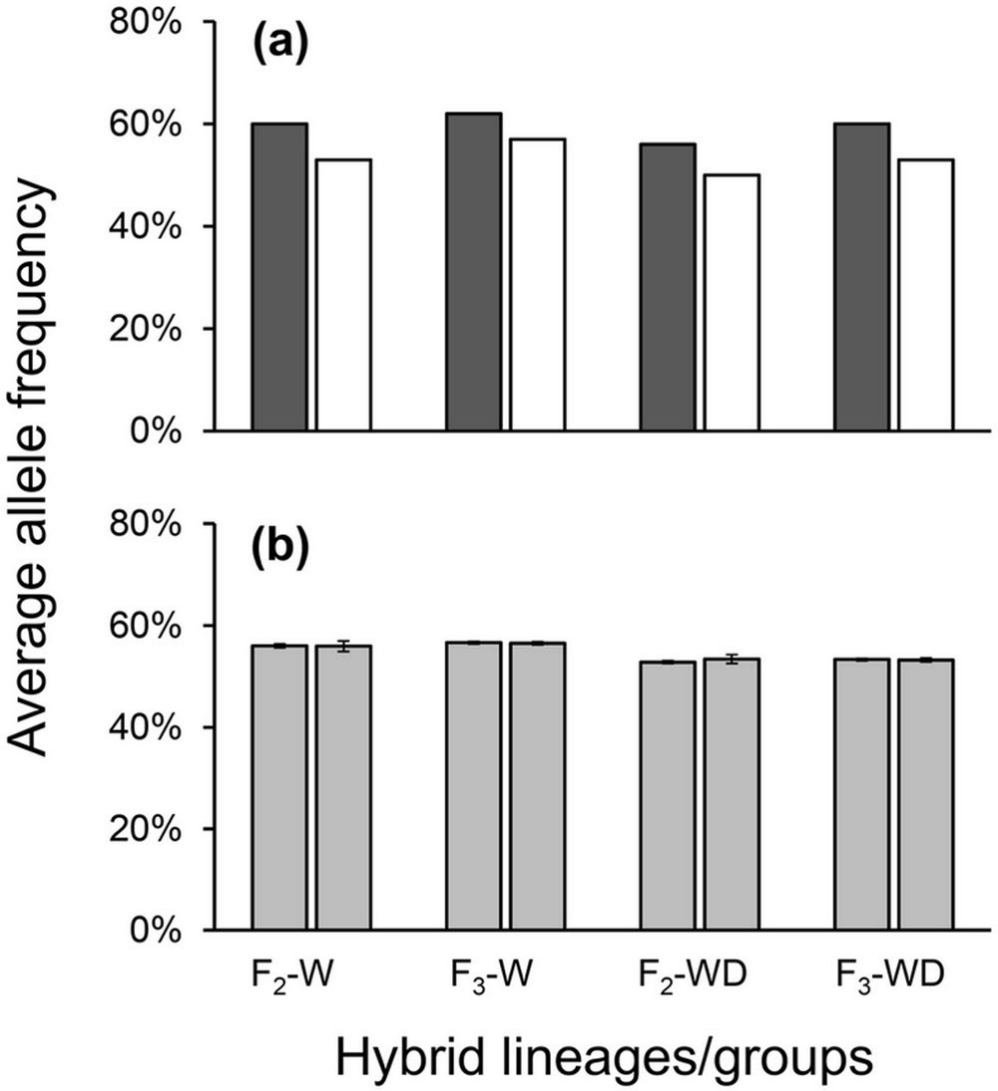
Average frequencies of crop-parent alleles in hybrid lineages (**a**) and groups (**b**) separated from an F_2_ or F_3_ hybrid population containing an *epsps* transgene. “W” represents wild rice (*Oryza rufipogon*); “WD” represents weedy rice. Dark-gray and white columns in (**a**) indicate lineages with and without a transgene, respectively. Light-gray columns in (**b**) indicate randomly formed groups. Bars indicate standard deviation.

In addition, we also detected much higher frequencies of alleles derived from the *epsps* rice parent in the transgenic F_2_ and F_3_ hybrid lineages than in their corresponding non-transgenic lineages (Fig. 4a,b), suggesting the influences of sampling transgene as an identifier on transmission of parent alleles at multiple loci. The bootstrap percentile method (BPM) analyses of each SSR locus indicated significant differences in parent-allele frequencies at multiple SSR loci between transgenic and non-transgenic crop-wild (46–52% loci), as well as crop-weed (28–59% loci) hybrid lineages (Supplementary Tables 1, 2). The neutrality test also confirmed the significant deviation of parental alleles from the theoretical values at most of these loci for both crop-wild and crop-weed hybrid lineages (Supplementary Tables 3, 4). Altogether, these results suggested that the separation of transgenic and non-transgenic hybrid lineages by using a transgene as an identifier caused non-random transmission of parental alleles into the resulted hybrid lineages.

Interestingly, we detected more alleles from cultivated rice in transgenic crop-wild hybrid lineages than in their corresponding non-transgenic lineages at the loci that showed significant differences in parental allele frequencies (Fig. 4a). Allele frequencies of cultivated rice were 60–62% in transgenic and 53–57% in non-transgenic crop-wild lineages (Fig. 4a). Similar results were also found in transgenic crop-weed hybrid lineages (Fig. 4a). In contrast, the two corresponding ideal groups of crop-wild/weed hybrid descendants did not show significant differences in crop parent-allele frequencies (Fig. 4b). These results suggested preferential transmission of crop alleles into transgenic hybrid lineages (F_2_ and F_3_), likely associated with the sampling of the transgene as an identifier to create transgenic and non-transgenic hybrid lineages.

### Non-random transmission of parental alleles into hybrid lineages with or without neutral identifiers

**Figure 5:**
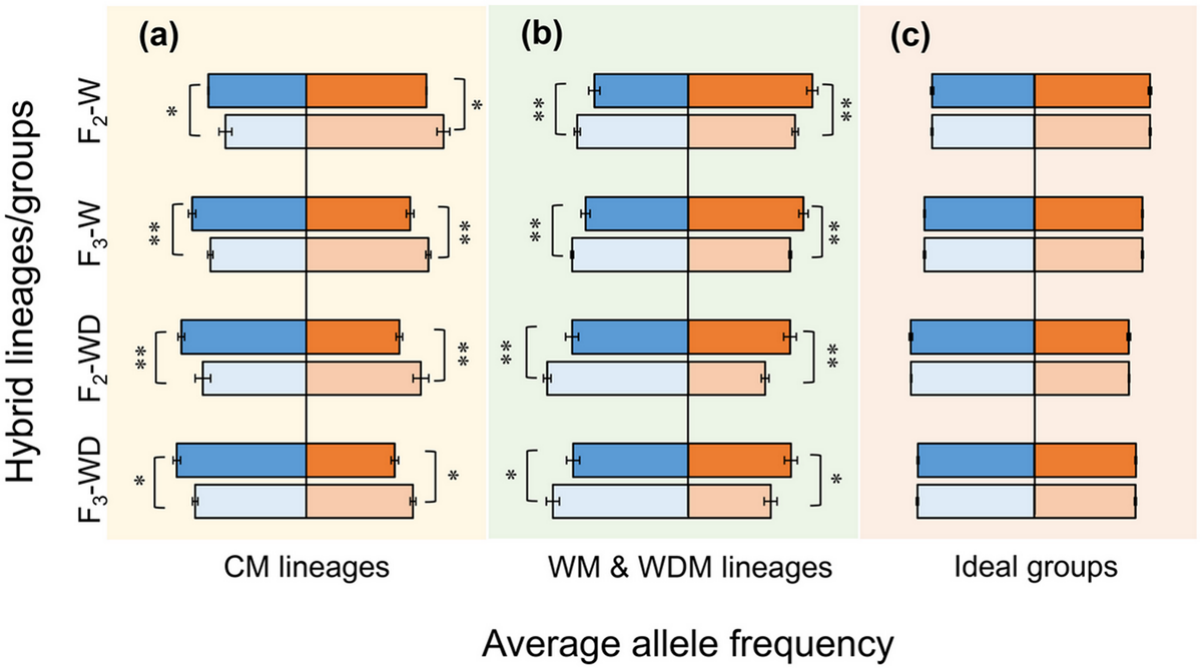
Average frequencies of parental alleles in hybrid lineages (**a**,**b**) and ideal groups (**c**) separated from a transgene-free F_2_ or F_3_ crop-wild (W) /weed (WD) hybrid population. (**a**) CM lineages are grouped by sampling individuals with crop-parent markers; (**b**) WM or WDM lineages are grouped by sampling individuals with wild- or weedy-parent markers; (**c**) Ideal groups are formed randomly. Blue and light-blue columns indicate frequencies of crop-parent alleles in lineages with and without markers, respectively; orange and light-orange columns indicate frequencies of wild- or weedy-parent alleles in lineages with and without markers, respectively. Bars indicate standard deviation. *,**Significance at *P* < 0.05 or *P* < 0.01, based on the one-tail paired *t* test.

We found considerable differences in genomic background between crop-wild/weed hybrid lineages with or without the identifiers when neutral SSR markers (identifiers) were used to create lineages in transgene-free hybrid combinations. This finding suggested that the non-random transmission of parental alleles into hybrid lineages might not necessarily be related to the transgene, but related any random identifiers (Table 1, Fig. 5a–c, Supplementary Tables 3–6).

Comparatively high *F*_*st*_ values were detected between the corresponding hybrid lineages with or without the three random selected identifiers, ranging from 0.02–0.03 for crop-wild lineages, and from 0.04–0.13 for crop-weed lineages (Table 1). In contrast, the *F*_*st*_ values between the corresponding ideal groups were < 0.001. These results suggested considerable genomic differentiation between hybrid lineages with or without the neutral identifier. The BPM analyses showed 19–71% SSR loci of which the parent-allele frequencies were significantly different (*P* < 0.05) between the corresponding crop-wild/weed hybrid lineages (Supplementary Tables 5, 6). The neutrality test also confirmed the significant deviation of parental alleles from the theoretical values in crop-wild/weed hybrid lineages (Table 1, Supplementary Tables 3, 4).

### Preferential transmission of parental alleles into hybrid lineages with selecting for a specific identifier

We found increased allelic frequencies of a parent in crop-wild and crop-weed hybrid lineages that were created by using a neutral identifier from this specific parent, suggesting that preferential allelic transmission from one parent was closely associated with the selection of an identifier located on the genome of this specific parent (Fig. 5a-c, Supplementary Tables 5–6).

Significantly higher (*P* < 0.05) average frequencies of crop alleles were detected in crop-wild hybrid lineages (45–53%) with the identifiers from the crop parent than in the hybrid lineages (37–44%) without the crop identifiers, based on the one-tail paired *t* test (Fig. 5a, Supplementary Table 5). Similar trends were also observed in crop-weed hybrid lineages (Fig. 5a, Supplementary Table 6). On the other hand, significantly higher average frequencies of wild alleles were detected in crop-wild hybrid lineages (53–57%) with the identifiers from the wild parent than in the lineages (47–49%) without the wild identifiers (Fig. 5b, Supplementary Table 5). Similar trends were also found in crop-weed hybrid lineages (Fig. 5b, Supplementary Table 6). However, no significant differences in allele frequencies were observed in the corresponding ideal groups of crop-wild/weed hybrid descendants (Fig. 5c).

## Discussion

Results from this study demonstrated evident genomic differentiation between transgenic and non-transgenic hybrid lineages divided by using the *epsps* transgene as an identifier, suggesting that the hybrid lineages/subpopulations created from a hybrid population using this method are not isogenic. This finding can well explain our previous observation about the phenotypical differences between transgenic and non-transgenic crop-wild hybrid seedlings (Fig. 2). Apparently, the selection of the transgenic identifier located on the genome of cultivated rice caused differences in the genomic background, as well as the phenotypes, between transgenic and non-transgenic hybrid lineages. Therefore, selecting a transgenic identifier from cultivated rice resulted in more traits from the crop parent that contained a transgene. In addition, different frequencies of parental alleles were found between transgenic hybrid lineages and their corresponding non-transgenic counterparts at multiple loci (Fig. 4), suggesting non-random transmission of parental alleles into hybrid lineages. The findings should be sound because a substantial amount of analytical data (Table 1, Fig. 4, Supplementary Tables 1–4) from F_2_-F_3_ hybrid descendants derived from artificial crosses between a GE *epsps* rice line and an accession of wild or weedy rice (*O. sativa* f. *spontanea*) was analyzed. In addition, we also included F_2_ and F_3_ hybrid descendants in this study to examine the consistency of the obtained results between independent generations. The obtained results support our first hypothesis that selecting a transgene as an identifier to produce “isogenic” hybrid lineages with or without a transgene can result in differences in their genomic background, although the influences of the transgene on genomic differentiation between hybrid lineages cannot be completely ruled out.

To circumvent the possible influences of the *epsps* transgene (Fig. 4) on genomic differentiation and non-random transmission of parental alleles, we used transgene-free crop-wild/weed hybrid lineages derived from crosses between a rice variety (Minghui-86) and a wild/weedy rice accession to determine the possible differences in the genomic background. Similar to the results obtained from transgenic hybrid lineages, we also detected significant differentiation in the genomic background of transgene-free hybrid lineages with or without the neutral identifiers used to separate hybrid descendants (F_2_-F_3_). The finding indicated that even without the involvement of a transgene, genomic differentiation still occurred between the identifier-present and -absent F_2_-F_3_ hybrid lineages separated by using the neutral identifiers. In addition, significant differences in frequencies of parental alleles were also found between the transgene-free hybrid lineages with or without the neutral identifiers at multiple loci. The differences in allele frequencies indicated that selecting a neutral identifier would also influence transmission of the parental alleles into hybrid lineages^27–30^. These findings based on a great amount of data (Table 1, Fig. 5, Supplementary Tables 3–6) support our second hypothesis that non-random transmission of parental alleles is not necessarily related to the selection of a transgenic identifier but most likely to any type of random identifiers.

The above findings indicate the strong influence of artificial sampling or selection of a target identifier, regardless of a transgene or other types of markers (e.g., neutral SSR markers), on genomic differentiation and non-random allelic transmission in the identifier-based lineages of hybrid descendants. In this study, the sampling of transgenic and non-transgenic hybrid lineages has played a critical role as a selection force, causing the preferential transmission of parental alleles that are genetically linked with the transgene into transgenic lineages, similar to the “hitchhiking effect” in evolution^27–30^. Remington *et al*.^31^ identified considerably altered genome-wide linkage disequilibrium patterns in maize by the artificial grouping of the maize lines into different subpopulations according to the regions of their origins from the total sample set^31^. This case study also indicates the strong influences of artificial selection on the allele assortment at multiple loci, supporting our findings of the non-random transmission of parental alleles in crop-wild/weed hybrid lineages. If this assumption holds true, the commonly used method to evaluate fitness of a transgene(s) by dividing F_2_/F_3_ or BC_1_ hybrid populations into transgenic and non-transgenic lineages^10, 17–22^ may create different genomic bases between the compared lineages for fitness evaluation. Therefore, the method may not be appropriate for the assessment of the environmental biosafety impact caused by transgene flow, even though it has been commonly applied to estimate fitness of a transgene(s) in different crop-wild and crop-weed hybrid combinations for decades^10, 17–22^. Thus, it is necessary to develop a more appropriate method for transgenic fitness evaluation, by considering differences in the genomic background between transgenic and non-transgenic hybrid lineages.

Interestingly, we also found preferential transmission of parental alleles into F_2_-F_3_ lineages in the transgene-free hybrid combinations, which was associated with the selection of a specific parental identifier. In other words, more alleles from a parent were detected at multiple loci in hybrid lineages having a neutral identifier from this specific parent, no matter it was used as a male or female parent. This result supports our third hypothesis about preferential allelic transmission from a parent, which is associated with the selection of an identifier from the specific parent. The preferential transmission of parental alleles at multiple loci is probably due to the positive selection or hitchhiking effect of these alleles that are genetically linked to the selected identifiers. The phenomena of association between the selected markers/genes and the genetically linked alleles in hybrid populations are widely reported in many plant species, such as rice and maize^28, 32, 33^. The observation demonstrates the critical role of artificial selection in increased frequencies of traits (genes) favored by human beings and other genetically linked alleles by the hitchhiking effect. In addition, model simulation also supported the presence of positive selection or “hitchhiking effect” between genetically linked alleles^27, 34^. The finding of preferential transmission of genetically linked alleles from a parent by selection may have its important implications in molecular marker-assisted selection in plant breeding, as indicated in the studies of tomatoes^35^, sour cherries^36^, rice^37^, and maize^38^. In addition, this finding has its significance in the studies of evolution under selection.

In summary, we found that using either a transgene or a neutral SSR marker as an identifier to separate a crop-wild or crop-weed hybrid population into transgene-present and -absent lineages/subpopulations can cause non-random transmission of the parental alleles at multiple loci, resulting in differences in the genomic background of the two hybrid lineages used for transgenic fitness evaluation. Our finding suggests that the commonly used method for transgene fitness assessment involving so-called isogenic F_2_/F_3_ or BC_1_ hybrid lineages with or without a transgene may not be appropriate for comparison, because of the differences in the genomic background of the compared hybrid lineages. We therefore propose that other means to evaluate fitness effects of transgene flow or introgression should be sought and designed to assess the environmental impact caused by crop-to-wild/weedy transgene flow, because the presumption of genomic equivalence between lineages of a segregated F_2_/F_3_ hybrid population cannot be justified. In addition, our finding of preferential parent-allele transmission into hybrid lineages at multiple loci also provides useful insights for understanding the strong effect of artificial selection of a marker (identifier), regardless of a transgene or a neutral allele, on parent-allele transmission into hybrid lineages. The generated knowledge also has important implications for molecular marker-assisted selection in plant breeding and for evolutionary studies by means of selection.

## Methods

### Creation of F_2_-F_3_ crop-wild and crop-weed hybrid lineages/groups

An herbicide-resistant GE rice line (EP3), its non-transgenic rice parent (Minghui-86), and a wild (one biotype from China, Jiangxi) and weedy rice accession (one biotype from Nepal), all containing the AA genome, were used to produce crop-wild/weedy hybrids. EP3 contained a transgene overexpressing *epsps* produced through agrobacterium-mediated transformation from Minghui-86^39^. EP3 was bred to T_5_ generation with one copy of the homozygous transgene^40^. For hybridization, cultivated rice line/variety was used as the male parents (pollen donors) and wild/weedy rice as the female parents (pollen recipients). All field experiments were conducted in the designated Biosafety Assessment Centers of Fujian Academy of Agricultural Sciences, Fuzhou, China.

**Figure 6:**
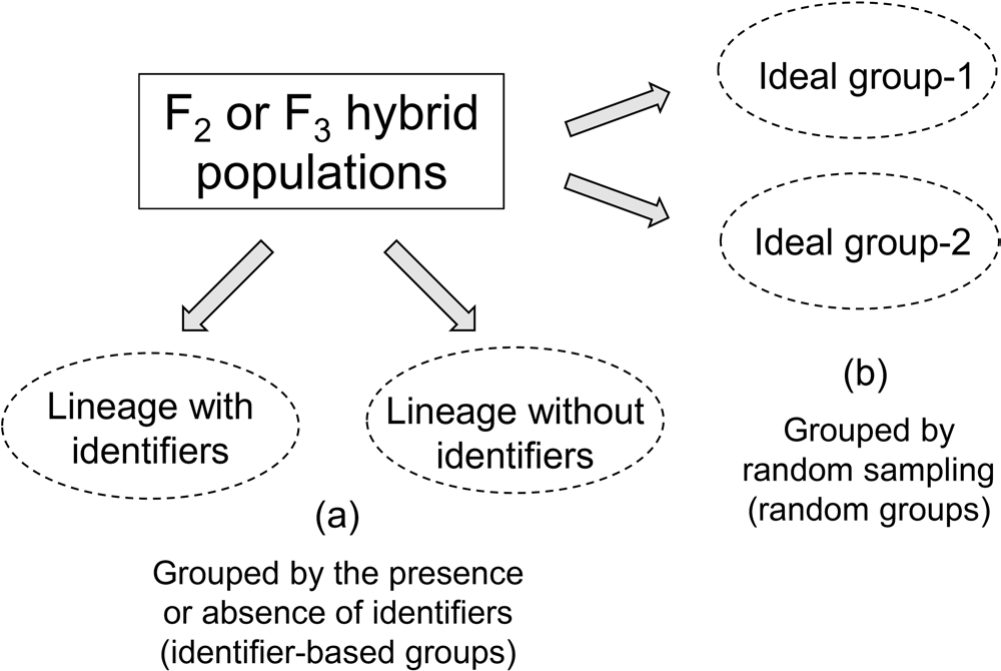
Hybrid lineages (**a**) and ideal groups (**b**) created from an experimental hybrid population (F_2_ or F_3_) for analyses. Identifier represents either a transgene or a neutral marker that are used to separate hybrid lineages.

For transgenic and non-transgenic hybrid combinations, F_2_ and F_3_ crop-wild and crop-weed descendants were included for analyses. The transgenic hybrid descendants were derived from F_1_ hybrids (~100 plants) between EP3 and wild or weedy rice independently through self-pollination. To create hybrid lineages (or subpopulations) with or without a transgene, we used the *epsps* transgene as an identifier to separate GE and non-GE lineages from the F_2_ (400 individuals) or F_3_ (600 individuals) crop-wild and crop-weed hybrid populations (Fig. 6a, identifier-based lineages). The presence and absence of the *epsps* transgene in each plant was identified through PCR (polymerase chain reactions) analyses following the method of Wang *et al*.^21^.

The non-transgenic F_2_ and F_3_ hybrid descendants were derived F_1_ hybrids (~100 plants) between Minghui-86 and wild or weedy rice independently through self-pollination. To create hybrid lineages with or without an identifier, we used neutral SSR markers as identifiers to separated identifier-present and -absent hybrid lineages from F_2_ (400 individuals) or F_3_ (600 individuals) hybrid populations (Fig. 6a, identifier-based lineages). These neutral SSR identifiers showed a Mendelian segregation pattern in the F_2_ and F_3_ crop-wild/weed hybrid populations. For the three sets of crop-wild hybrid lineages, three neutral SSR identifiers (RM572, RM408 and RM277) were used for PCR analyses^41^, while for the three sets of crop-weed hybrid lineages, other three neutral SSR identifiers (RM186, RM172, and RM3533) were used.

In addition, we also created ideal groups (or subpopulations) (Fig. 6b, random groups) from the F_2_ and F_3_ crop-wild/weedy hybrid populations, as a reference to the identifier-based lineages. To create the ideal groups, we randomly drew individuals from the F_2_ (400 individuals) or F_3_ (600 individuals) hybrid populations for ten times to ensure minimal variation (with standard deviation < 0.01) in parent-allele frequencies between the two ideal groups (group-1 and -2) in each hybrid populations.

### PCR amplification and data collection

Total genomic DNA of all F_2_ and F_3_ crop-wild/weed hybrid samples were extracted from leaf tissues followed the CTAB protocol of Murray & Thompson^42^. Fifty-two and 32 rice SSR markers that were randomly distributed on the rice genome with physical distances > 800, 000 bp (Fig. 3) and polymorphic between the cultivated rice and wild/weedy rice parents were included for analyses. SSR markers were selected from the Gramene Database (http://www.gramene.org), with information on their primer sequences (Supplementary Table 7). The forward primers were fluorescently labeled to visualize the PCR products by FAM (blue), ROX (red), or JOE (green)^41, 43^. PCR was carried out using a 2720 Thermal Cycler Set (Applied Biosystems) following the method of Jiang *et al*. (2012) and Yang *et al*. (2014)^41, 43^. PCR products were separated by capillary electrophoresis with fluorescence on an ABI 3130 DNA Analyzer (Applied Biosystems). The electrophoretic outputs were scored as genotype data using the software GeneMapper version 4.0 (Applied Biosystems). Frequencies of parental alleles in hybrid lineages at each locus were calculated to estimate allele transmission.

### Data analysis

The diversity coefficient (*F*_*st*_) between hybrid lineages/groups was analyzed using the software GenAlEx version 6.5 to estimate their genetic differentiation^44^. The bootstrap percentile method (BPM)^45, 46^ was used to determine differences in frequencies of parental alleles at each SSR loci between crop-wild/weed hybrid lineages/groups, at the significant level of *P* < 0.05. The sampling size for bootstrap of each hybrid lineage/group was determined by its sample size^45, 46^, with re-sampling replicates for 10,000, using the Microsoft Excel 2013 Visual Basic for Applications^47^. The one-tail paired *t* test was applied to determine differences in average frequencies of parental alleles between hybrid lineages with or without the crop or wild/weedy identifiers for the loci with significant differences in parental allele frequencies based on the BPM analyses. The neutrality test was conducted to test the deviation of alleles from the theoretical values at each locus in corresponding hybrid lineages/groups through 1,000 simulations using the program PopGene Version 1.31^48^, at the significant level of *P* < 0.05.

### Data availability

Files with data are available on Data Dryad.

## Electronic supplementary material

Supplementary Information
