# Supplementary material for: Non-random transmission of parental alleles into crop-wild and crop-weed hybrid lineages separated by a transgene and neutral identifiers in rice
Source: Sci Rep. 2017 Sep 5;7:10436. doi: 10.1038/s41598-017-10596-4 (PMC5585250; doi:10.1038/s41598-017-10596-4)
Supplement: Supplementary file 1 — Supplementary Information [file 41598_2017_10596_MOESM1_ESM.pdf]

Non-random transmission of parental alleles into crop-wild and crop-weed hybrid lineages separated by a transgene and neutral identifiers in rice

Zhe Wang, Lei Wang, Zhi Wang, Bao-Rong Lu*

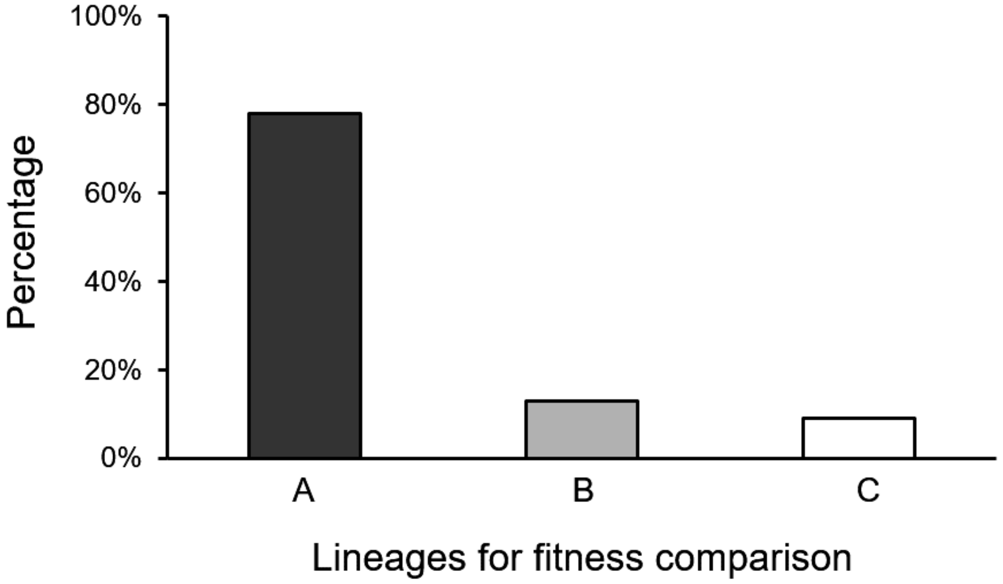

Supplementary Figure 1. Percentage of research articles in which different types of plant materials with the same genomes were used to estimate transgenic fitness, based on the 32 relevant research articles of recent publications (2000-2017) from a literature survey of the Web of Science (apps.webofknowledge.com/). A: Comparison between isogenic hybrid lineages (including F_2-F_3/BC_1) with or without a transgene created by separating the lineages using a transgene as an identifier. B: Comparison between parents of wild relatives and transgenic hybrids. C: Comparison between GE crop-wild/weed hybrids and non-GE crop-wild/weed hybrids.

Please see the following relevant references obtained from the literature survey, using different combinations of the key words: transgene, transgenic, fitness, hybrid.

1. Burke, J. M., & Rieseberg, L. H. Fitness effects of transgenic disease resistance in sunflowers. *Science.* **300**, 1250-1250 (2003).
2. Busi, R., & Powles, S. B. Transgenic glyphosate-resistant canola (*Brassica napus*) can persist outside agricultural fields in Australia. *Agriculture Ecosystems & Environment*. **220**, 28-34 (2016).
3. Cao, Q. J., Xia, H., Yang, X. & Lu, B. R. Performance of hybrids between weedy rice and insect-resistant transgenic rice under field experiments: implication for environmental biosafety assessment. *J. Integr. Plant Biol*. **51**, 1138-1148 (2009).
4. Chun, Y. J. et al. Gene flow from herbicide-tolerant GM rice and the heterosis of GM rice-weed F2 progeny. *Planta*, **233**, 807-815 (2011).
5. Clegg, J., Ellstrand, N. C. & Guadagnuolo, R. Relative Fitness of Transgenic vs. Non-Transgenic Maize ×Teosinte Hybrids: a Field Evalutation. *Ecol. Appl*. **16**, 1967-1974 (2006).
6. Dong, S. S. *et al*. No effect of transgene and strong wild parent effects on seed dormancy in crop-wild hybrids of rice: implications for transgene persistence in wild populations. *Ann. Appl. Biol*. **159**, 348-357 (2011).
7. Dong, S. S. *et al*. Persistence of transgenes in wild rice populations depends on the interaction between genetic background of recipients and environmental conditions. *Ann. Appl. Biol*. Doi:10.1111/aab.12365 (2017).

8. Fuchs, M., Chirco, E. M., Mcferson, J. R. & Gonsalves, D. Comparative fitness of a wild squash species and three generations of hybrids between wild × virus-resistant transgenic squash. *Environmental Biosafety Res*. **3**, 17-28 (2004).
9. Guadagnuolo, R., Clegg, J. & Ellstrand, N. C. Relative fitness of transgenic vs. non-transgenic maize × teosinte hybrids: a field evaluation. *Ecol. Appl.* **16**, 1967-74 (2006).
10. Guan, Z. J. *et al*. Performance of hybrid progeny formed between genetically modified herbicide-tolerant soybean and its wild ancestor. *AoB Plants*. **7** (2015).
11. Halfhill, M. D. et al. Growth, productivity, and competitiveness of introgressed weedy Brassica rapa hybrids selected for the presence of *Bt cry1Ac* and *gfp* transgenes. *Mol Ecol*. **14**, 3177-3189 (2005).
12. Harvey-Samuel, T., Ant, T., Gong, H., Morrison, N. I., & Alphey, L. Population-level effects of fitness costs associated with repressible female-lethal transgene insertions in two pest insects. *Evol Appl*. **7**, 597-606 (2014).
13. Laughlin, K. D., Power, A. G., Snow, A. A. & Spencer, L. J. Risk assessment of genetically engineered crops: fitness effects of virus-resistance transgenes in wild *cucurbita pepo*. *Ecol. Appl*. **19**, 1091-101 (2009).
14. Li, L. *et al*. Limited ecological risk of insect-resistance transgene flow from cultivated rice to its wild ancestor based on life-cycle fitness assessment. *Sci. Bull.* **61**, 1440-1450 (2016).
15. Liu, Y. B. *et al*. The effect of *Bt* -transgene introgression on plant growth and reproduction in wild *Brassica juncea*. *Transgenic Res*. **24**, 537-547 (2014).
16. Londo, J. P., Bollman, M. A., Sagers, C. L., Lee, E. H. & Watrud, L. S. Changes in fitness-associated traits due to the stacking of transgenic glyphosate resistance and insect resistance in *brassica napus*. *Heredity*. **107**, 328-37 (2011).
17. Londo, J. P., Bautista, N. S., Sagers, C. L., Lee, E. H. & Watrud, L. S. Glyphosate drift promotes changes in fitness and transgene gene flow in canola (*Brassica napus*) and hybrids. *Ann. Bot*. **106**, 957-965 (2010).
18. Magomere, T. *et al*. Evaluation of fitness in F_2 generations of *Africa biofortified sorghum* event 188 and weedy *Sorghum bicolor, ssp. drummondii*. *Electron. J. Biotechn.* **22**, 52-61 (2016).
19. Oard, J., Cohn, M. A., Linscombe, S., Gealy, D. & Gravois, K. Field evaluation of seed production, shattering, and dormancy in hybrid populations of transgenic rice (*Oryza sativa*) and the weed, red rice (*Oryza sativa*). *Plant Sci.* **157**, 13-22 (2000).
20. Shengnan, L., Xiaoling, S., Yanhua, H., Weiming, D. & Sheng, Q. Fitness of hybrids between two types of transgenic rice and six and weed rice accessions. *Crop Sci*. **56**, 2751-2765 (2016).
21. Snow, A. A. *et al*. A *Bt* transgene reduces herbivory and enhances fecundity in wild sunflowers. *Ecol Appl*. **13**, 279-286 (2003).
22. Tian, D., Traw, M. B., Chen, J. Q., Kreitman, M., & Bergelson, J. Fitness costs of R-gene-mediated resistance in *Arabidopsis thaliana*. *Nature*. **423**, 74-77 (2003).
23. Ting, P. *et al*. Reproductive fitness of outcrossed hybrids between transgenic Broccoli (*Brassica oleracea*) carrying the *ipt* transgene and conventional varieties of kale, broccoli and cauliflower. *Pak. J. Bot*. **46**, 1437-1444 (2014).
24. Wang, W. *et al*. A novel 5-enolpyruvoylshikimate-3-phosphate (*epsp*) synthase transgene for glyphosate resistance stimulates growth and fecundity in weedy rice (*Oryza sativa*) without herbicide. *New Phytol.* **202**, 679-88 (2014).
25. Yang, X. *et al*. Transgenes for insect resistance reduce herbivory and enhance fecundity in advanced generations of crop-weed hybrids of rice. *Evol. Appl*. **4**, 672-684 (2011).

26. Yang, X., Wang, F., Su, J. & Lu, B.- R. Limited fitness advantages of crop-weed hybrid progeny containing insect-resistant transgenes (*Bt/Cpti*) in transgenic rice field. *Plos One*. **7**, 398-398 (2012).
27. Yang, X. *et al*. Efficacy of insect-resistance *Bt/CpTI* transgenes in F_5-F_7 generations of rice crop-weed hybrid progeny: implications for assessing ecological impact of transgene flow. *Sci. Bull*. **60**, 1563-1571 (2015).
28. Xia, H. *et al*. Ambient insect pressure and recipient genotypes determine fecundity of transgenic crop-weed rice hybrid progeny: Implications for environmental biosafety assessment. *Evol Appl*. **9**, 847-856 (2016).
29. Warwick, S. I., Legere, A., Simard, M. J. & James, T. Do escaped transgenes persist in nature? The case of an herbicide resistance transgene in a weedy *Brassica rapa* population. *Mol. Ecol*. **17**, 1387-1395 (2008).
30. Olguin, E. R. S., Arrieta-Espinoza, G., Lobo, J. A., & Espinoza-Esquivel, A. M. Assessment of gene flow from a herbicide-resistant *indica* rice (*Oryza sativa L.*) to the Costa Rican weedy rice (*Oryza sativa*) in Tropical America: factors affecting hybridization rates and characterization of F_1 hybrids. *Transgenic Res*. **18**, 633-647 (2009).
31. Zhang, N., Linscombe, S. & Oard, J. Out-crossing frequency and genetic analysis of hybrids between transgenic glufosinate herbicide-resistant rice and the weed, red rice. *Euphytica*. **130**, 35-45(2003).
32. Zuo, J., Qiang, S., & Song, X. L. Fitness of progenies between transgenic rice and weedy rice under greenhouse conditions. *Rice Sci*. **24**, 608-616 (2010).

Supplementary Table 1 Parent-allele frequencies (crop : wild) in hybrid lineages with (GE) or without the transgene (non-GE) in F_2 and F_3 crop-wild populations, compared with the ideal groups. Frequencies in bold indicate significant differences (P <0.05) between lineages with (GE) or without a transgene (non-GE) grouped from an F_2/F_3 hybrid population.

Primer name	F_2 GE	F_2 non-GE	F_3 GE	F_3 non-GE
RM84	**0.78 : 0.22**	**0.66 : 0.34**	0.63 : 0.37	0.60 : 0.40
RM575	**0.58 : 0.42**	**0.49 : 0.51**	0.53 : 0.47	0.52 : 0.48
RM572	**0.58 : 0.42**	**0.49 : 0.51**	0.51 : 0.49	0.51 : 0.49
RM9	**0.65 : 0.35**	**0.57 : 0.43**	0.52 : 0.48	0.49 : 0.51
RM5	**0.58 : 0.42**	**0.66 : 0.34**	0.59 : 0.41	0.55 : 0.45
RM212	0.46 : 0.54	0.40 : 0.60	0.46 : 0.54	0.41 : 0.59
RM6895	0.79 : 0.21	0.75 : 0.25	**0.69 : 0.31**	**0.54 : 0.46**
RM7245	**0.52 : 0.48**	**0.60 : 0.40**	**0.60 : 0.40**	**0.70 : 0.30**
RM523	0.54 : 0.46	0.54 : 0.46	0.64 : 0.36	0.61 : 0.39
RM282	0.40 : 0.60	0.41 : 0.59	0.28 : 0.72	0.25 : 0.75
RM16	0.60 : 0.40	0.61 : 0.39	**0.63 : 0.37**	**0.49 : 0.51**
RM186	0.53 : 0.47	0.50 : 0.50	**0.55 : 0.45**	**0.49 : 0.51**
RM468	**0.47 : 0.53**	**0.53 : 0.47**	0.55 : 0.45	0.53 : 0.47
RM6395	**0.76 : 0.24**	**0.53 : 0.47**	0.66 : 0.34	0.65 : 0.35
RM6089	**0.38 : 0.62**	**0.49 : 0.51**	0.44 : 0.56	0.44 : 0.56
RM3558	0.61 : 0.39	0.60 : 0.40	0.52 : 0.48	0.49 : 0.51
RM6748	**0.56 : 0.44**	**0.50 : 0.50**	0.65 : 0.35	0.62 : 0.38
RM3419	**0.56 : 0.44**	**0.64 : 0.36**	**0.54 : 0.46**	**0.68 : 0.32**
RM6841	0.87 : 0.13	0.85 : 0.15	**0.69 : 0.31**	**0.83 : 0.17**
RM190	0.37 : 0.63	0.31 : 0.69	**0.59 : 0.41**	**0.53 : 0.47**
RM276	**0.75 : 0.25**	**0.47 : 0.53**	**0.72 : 0.28**	**0.49 : 0.51**
RM214	**0.54 : 0.46**	**0.47 : 0.53**	**0.55 : 0.45**	**0.47 : 0.53**
RM11	0.53 : 0.47	0.59 : 0.41	0.65 : 0.35	0.66 : 0.34
RM505	0.64 : 0.36	0.59 : 0.41	0.68 : 0.32	0.65 : 0.35
RM172	**0.50 : 0.50**	**0.44 : 0.56**	**0.81 : 0.19**	**0.72 : 0.68**
RM408	0.59 : 0.41	0.64 : 0.36	0.49 : 0.51	0.55 : 0.45
RM5068	0.19 : 0.81	0.18 : 0.82	**0.28 : 0.72**	**0.37 : 0.63**
RM331	0.70 : 0.26	0.75 : 0.25	**0.50 : 0.50**	**0.67 : 0.33**
RM296	**0.68 : 0.32**	**0.81 : 0.19**	**0.56 : 0.44**	**0.67 : 0.33**
RM4455	**0.35 : 0.65**	**0.52 : 0.48**	0.58 : 0.42	0.58 : 0.42
RM7557	**0.66 : 0.34**	**0.55 : 0.45**	**0.59 : 0.41**	**0.47 : 0.53**
RM332	0.57 : 0.43	0.57 : 0.43	0.60 : 0.40	0.54 : 0.46
RM4862	**0.83 : 0.17**	**0.76 : 0.24**	**0.82 : 0.18**	**0.77 : 0.23**
RM224	0.51 : 0.49	0.57 : 0.43	**0.58 : 0.42**	**0.64 : 0.36**
RM8216	**0.57 : 0.43**	**0.51 : 0.49**	0.70 : 0.30	0.70 : 0.30
RM3483	0.75 : 0.25	0.75 : 0.25	0.73 : 0.27	0.72 : 0.28
RM6296	0.58 : 0.42	0.57 : 0.43	0.62 : 0.38	0.59 : 0.41

RM277	0.53 : 0.47	0.48 : 0.52	**0.53 : 0.47**	**0.43 : 0.57**
RM6732	**0.35 : 0.65**	**0.25 : 0.75**	0.53 : 0.47	0.51 : 0.49
RM17	0.68 : 0.32	0.70 : 0.30	0.64 : 0.36	0.63 : 0.37
RM111	0.69 : 0.31	0.68 : 0.32	**0.68 : 0.32**	**0.53 : 0.47**
RM143	0.47 : 0.53	0.49 : 0.51	0.53 : 0.47	0.54 : 0.46
RM333	**0.84 : 0.16**	**0.61 : 0.39**	**0.72 : 0.28**	**0.61 : 0.39**
RM4128	**0.63 : 0.37**	**0.43 : 0.57**	**0.82 : 0.18**	**0.64 : 0.36**
RM168	**0.62 : 0.43**	**0.52 : 0.48**	**0.66 : 0.34**	**0.53 : 0.47**
RM565	**0.41 : 0.59**	**0.32 : 0.68**	**0.41 : 0.59**	**0.34 : 0.66**
RM16074	0.52 : 0.48	0.51 : 0.49	0.51 : 0.49	0.49 : 0.51
RM20468	**0.59 : 0.41**	**0.39 : 0.61**	**0.62 : 0.38**	**0.51 : 0.49**
RM130	0.59 : 0.41	0.60 : 0.40	0.53 : 0.47	0.57 : 0.43
RM20460	**0.60 : 0.40**	**0.38 : 0.62**	0.51 : 0.49	0.52 : 0.48
RM5371	**0.76 : 0.24**	**0.50 : 0.50**	**0.70 : 0.30**	**0.59 : 0.41**
RM16071	0.67 : 0.33	0.66 : 0.34	0.58 : 0.42	0.54 : 0.46
Average	0.58 : 0.42	0.55 : 0.45	0.59 : 0.41	0.56 : 0.44

Primer name	F_2 ideal-1	F_2 ideal-2	F_3 ideal-1	F_3 ideal-2
RM84	0.79 : 0.21	0.78 : 0.22	0.61 : 0.39	0.59 : 0.41
RM575	0.58 : 0.42	0.55 : 0.45	0.50 : 0.50	0.51 : 0.49
RM572	0.53 : 0.47	0.52 : 0.48	0.49 : 0.51	0.49 : 0.51
RM9	0.61 : 0.39	0.63 : 0.37	0.51 : 0.49	0.52 : 0.48
RM5	0.57 : 0.43	0.58 : 0.42	0.57 : 0.43	0.59 : 0.41
RM212	0.39 : 0.61	0.39 : 0.61	0.44 : 0.56	0.43 : 0.57
RM6895	0.81 : 0.19	0.81 : 0.19	0.59 : 0.41	0.58 : 0.42
RM7245	0.52 : 0.48	0.53 : 0.47	0.65 : 0.35	0.65 : 0.35
RM523	0.47 : 0.53	0.50 : 0.50	0.62 : 0.38	0.62 : 0.38
RM282	0.44 : 0.56	0.42 : 0.58	0.26 : 0.74	0.27 : 0.73
RM16	0.59 : 0.41	0.58 : 0.42	0.58 : 0.42	0.57 : 0.43
RM186	0.52 : 0.48	0.53 : 0.47	0.52 : 0.48	0.52 : 0.48
RM468	0.47 : 0.53	0.48 : 0.52	0.56 : 0.44	0.54 : 0.46
RM6395	0.72 : 0.28	0.71 : 0.29	0.66 : 0.34	0.65 : 0.35
RM6089	0.37 : 0.63	0.38 : 0.62	0.44 : 0.56	0.43 : 0.57
RM3558	0.62 : 0.38	0.62 : 0.38	0.51 : 0.49	0.50 : 0.50
RM6748	0.52 : 0.48	0.52 : 0.48	0.63 : 0.37	0.64 : 0.36
RM3419	0.56 : 0.44	0.55 : 0.45	0.58 : 0.42	0.59 : 0.41
RM6841	0.87 : 0.13	0.85 : 0.15	0.74 : 0.26	0.73 : 0.27
RM190	0.33 : 0.67	0.30 : 0.70	0.56 : 0.44	0.56 : 0.44
RM276	0.69 : 0.31	0.70 : 0.30	0.64 : 0.36	0.65 : 0.35
RM214	0.51 : 0.49	0.51 : 0.49	0.51 : 0.49	0.50 : 0.50
RM11	0.55 : 0.45	0.55 : 0.45	0.66 : 0.34	0.65 : 0.35
RM505	0.62 : 0.38	0.62 : 0.38	0.72 : 0.28	0.72 : 0.28
RM172	0.47 : 0.53	0.48 : 0.52	0.65 : 0.35	0.66 : 0.34
RM408	0.58 : 0.42	0.58 : 0.42	0.50 : 0.50	0.50 : 0.50

RM5068	0.20 : 0.80	0.21 : 0.79	0.30 : 0.70	0.30 : 0.70
RM331	0.78 : 0.22	0.80 : 0.20	0.57 : 0.43	0.55 : 0.45
RM296	0.80 : 0.20	0.79 : 0.21	0.60 : 0.40	0.60 : 0.40
RM4455	0.34 : 0.66	0.36 : 0.64	0.58 : 0.42	0.59 : 0.41
RM7557	0.64 : 0.36	0.65 : 0.35	0.52 : 0.48	0.51 : 0.49
RM332	0.56 : 0.44	0.55 : 0.45	0.57 : 0.43	0.57 : 0.43
RM4862	0.79 : 0.21	0.79 : 0.21	0.80 : 0.20	0.79 : 0.21
RM224	0.50 : 0.50	0.50 : 0.50	0.60 : 0.40	0.62 : 0.38
RM8216	0.61 : 0.39	0.59 : 0.41	0.70 : 0.30	0.71 : 0.29
RM3483	0.76 : 0.24	0.75 : 0.25	0.73 : 0.27	0.72 : 0.28
RM6296	0.57 : 0.43	0.56 : 0.44	0.62 : 0.38	0.62 : 0.38
RM277	0.54 : 0.46	0.55 : 0.45	0.50 : 0.50	0.49 : 0.51
RM6732	0.34 : 0.66	0.32 : 0.68	0.52 : 0.48	0.53 : 0.47
RM17	0.66 : 0.34	0.67 : 0.33	0.63 : 0.37	0.63 : 0.37
RM111	0.67 : 0.33	0.63 : 0.37	0.62 : 0.38	0.64 : 0.36
RM143	0.47 : 0.53	0.49 : 0.51	0.55 : 0.45	0.54 : 0.46
RM333	0.79 : 0.21	0.78 : 0.22	0.67 : 0.33	0.67 : 0.33
RM4128	0.54 : 0.46	0.55 : 0.45	0.76 : 0.24	0.76 : 0.24
RM168	0.55 : 0.45	0.56 : 0.44	0.61 : 0.39	0.60 : 0.40
RM565	0.42 : 0.58	0.43 : 0.57	0.38 : 0.62	0.36 : 0.64
RM16074	0.51 : 0.49	0.51 : 0.49	0.50 : 0.50	0.49 : 0.51
RM20468	0.54 : 0.46	0.52 : 0.48	0.52 : 0.48	0.51 : 0.49
RM130	0.58 : 0.42	0.57 : 0.43	0.55 : 0.45	0.53 : 0.47
RM20460	0.55 : 0.45	0.54 : 0.46	0.53 : 0.47	0.51 : 0.49
RM5371	0.68 : 0.32	0.67 : 0.33	0.60 : 0.40	0.60 : 0.40
RM16071	0.65 : 0.35	0.67 : 0.33	0.55 : 0.45	0.54 : 0.46
Average	0.56 : 0.44	0.56 : 0.44	0.57 : 0.43	0.57 : 0.43

Supplementary Table 2 Parent-allele frequencies (crop : weed) in hybrid lineages with (GE) or without the transgene (non-GE) in F_2 and F_3 crop-weed populations, compared with the ideal groups. Frequencies in bold indicate significant differences (*P* <0.05) between lineages with (GE) or without a transgene (non-GE) grouped from an F_2/F_3 hybrid population.

Primer name	F_2 GE	F_2 non-GE	F_3 GE	F_3 non-GE
RM84	**0.66 : 0.34**	**0.45 : 0.55**	**0.68 : 0.32**	**0.52 : 0.48**
RM283	0.48 : 0.52	0.47 : 0.53	**0.57 : 0.43**	**0.13 : 0.87**
RM23	**0.68 : 0.32**	**0.47 : 0.53**	**0.54 : 0.46**	**0.41 : 0.59**
RM5	**0.94 : 0.06**	**0.85 : 0.15**	**0.68 : 0.32**	**0.60 : 0.40**
RM128	**0.61 : 0.39**	**0.54 : 0.46**	0.56 : 0.44	0.53 : 0.47
RM5356	**0.59 : 0.41**	**0.47 : 0.53**	0.55 : 0.45	0.54 : 0.46
RM5390	0.45 : 0.55	0.51 : 0.49	0.42 : 0.58	0.44 : 0.56
RM530	**0.46 : 0.54**	**0.62 : 0.38**	0.39 : 0.61	0.37 : 0.63
RM231	0.60 : 0.40	0.62 : 0.38	0.57 : 0.43	0.59 : 0.41
RM16	0.57 : 0.43	0.62 : 0.38	0.54 : 0.46	0.57 : 0.43
RM8277	0.55 : 0.45	0.56 : 0.44	0.54 : 0.46	0.57 : 0.43
RM168	**0.49 : 0.51**	**0.43 : 0.57**	**0.63 : 0.37**	**0.57 : 0.43**
RM186	0.53 : 0.47	0.53 : 0.47	0.50 : 0.50	0.51 : 0.49
RM520	**0.64 : 0.36**	**0.56 : 0.44**	0.66 : 0.34	0.63 : 0.37
RM5320	0.30 : 0.70	0.32 : 0.68	0.41 : 0.59	0.44 : 0.56
RM211	**0.56 : 0.44**	**0.47 : 0.53**	0.54 : 0.46	0.57 : 0.43
RM190	**0.48 : 0.52**	**0.41 : 0.59**	0.44 : 0.56	0.46 : 0.54
RM587	0.55 : 0.45	0.50 : 0.50	0.60 : 0.40	0.58 : 0.42
RM4128	**0.70 : 0.30**	**0.78 : 0.22**	**0.64 : 0.36**	**0.74 : 0.26**
RM527	0.57 : 0.43	0.52 : 0.48	0.57 : 0.43	0.56 : 0.44
RM340	**0.51 : 0.49**	**0.60 : 0.40**	**0.50 : 0.50**	**0.63 : 0.37**
RM295	0.85 : 0.15	0.83 : 0.17	**0.55 : 0.45**	**0.63 : 0.37**
RM214	**0.33 : 0.67**	**0.45 : 0.55**	0.41 : 0.59	0.44 : 0.56
RM172	**0.54 : 0.46**	**0.48 : 0.52**	0.51 : 0.49	0.48 : 0.52
RM8243	**0.58 : 0.42**	**0.45 : 0.55**	**0.62 : 0.38**	**0.53 : 0.47**
RM447	**0.54 : 0.46**	**0.60 : 0.40**	0.50 : 0.50	0.48 : 0.52
RM8206	0.38 : 0.62	0.42 : 0.58	0.62 : 0.38	0.60 : 0.40
RM296	**0.35 : 0.65**	**0.43 : 0.57**	0.42 : 0.58	0.38 : 0.62
RM4862	**0.59 : 0.41**	**0.37 : 0.63**	0.44 : 0.56	0.41 : 0.59
RM3117	0.40 : 0.60	0.39 : 0.61	0.90 : 0.10	0.90 : 0.10
RM7102	**0.54 : 0.46**	**0.04 : 0.96**	0.48 : 0.52	0.45 : 0.55
RM3533	0.54 : 0.46	0.60 : 0.40	0.49 : 0.51	0.44 : 0.56
Average	0.55 : 0.45	0.51 : 0.49	0.55 : 0.45	0.52 : 0.49

Primer name	F_2 ideal-1	F_2 ideal-2	F_3 ideal-1	F_3 ideal-2
RM84	0.51 : 0.49	0.52 : 0.48	0.47 : 0.53	0.46 : 0.54
RM283	0.43 : 0.57	0.43 : 0.57	0.44 : 0.56	0.43 : 0.57
RM23	0.52 : 0.48	0.54 : 0.46	0.51 : 0.49	0.50 : 0.50

RM5	0.92 : 0.08	0.92 : 0.08	0.66 : 0.34	0.66 : 0.34
RM128	0.59 : 0.41	0.61 : 0.39	0.56 : 0.44	0.55 : 0.45
RM5356	0.48 : 0.52	0.49 : 0.51	0.54 : 0.46	0.55 : 0.45
RM5390	0.39 : 0.61	0.41 : 0.59	0.43 : 0.57	0.42 : 0.58
RM530	0.40 : 0.60	0.39 : 0.61	0.39 : 0.61	0.37 : 0.63
RM231	0.60 : 0.40	0.61 : 0.39	0.58 : 0.42	0.58 : 0.42
RM16	0.57 : 0.43	0.60 : 0.40	0.55 : 0.45	0.54 : 0.46
RM8277	0.54 : 0.46	0.57 : 0.43	0.55 : 0.45	0.54 : 0.46
RM168	0.44 : 0.56	0.46 : 0.54	0.60 : 0.40	0.62 : 0.38
RM186	0.52 : 0.48	0.54 : 0.46	0.51 : 0.49	0.50 : 0.50
RM520	0.62 : 0.38	0.63 : 0.37	0.65 : 0.35	0.65 : 0.35
RM5320	0.30 : 0.70	0.30 : 0.70	0.43 : 0.57	0.41 : 0.59
RM211	0.54 : 0.46	0.54 : 0.46	0.56 : 0.44	0.55 : 0.45
RM190	0.46 : 0.54	0.48 : 0.52	0.45 : 0.55	0.45 : 0.55
RM587	0.55 : 0.45	0.52 : 0.48	0.59 : 0.41	0.59 : 0.41
RM4128	0.72 : 0.28	0.72 : 0.28	0.67 : 0.33	0.67 : 0.33
RM527	0.56 : 0.44	0.55 : 0.45	0.57 : 0.43	0.57 : 0.43
RM340	0.53 : 0.47	0.53 : 0.47	0.54 : 0.46	0.55 : 0.45
RM295	0.54 : 0.46	0.56 : 0.44	0.58 : 0.42	0.57 : 0.43
RM214	0.36 : 0.64	0.38 : 0.62	0.41 : 0.59	0.42 : 0.58
RM172	0.53 : 0.47	0.54 : 0.46	0.50 : 0.50	0.50 : 0.50
RM8243	0.56 : 0.44	0.54 : 0.46	0.58 : 0.42	0.60 : 0.40
RM447	0.56 : 0.44	0.55 : 0.45	0.49 : 0.51	0.50 : 0.50
RM8206	0.39 : 0.61	0.39 : 0.61	0.61 : 0.39	0.61 : 0.39
RM296	0.37 : 0.63	0.38 : 0.62	0.41 : 0.59	0.41 : 0.59
RM4862	0.54 : 0.46	0.54 : 0.46	0.43 : 0.57	0.43 : 0.57
RM3117	0.84 : 0.16	0.85 : 0.15	0.89 : 0.11	0.91 : 0.09
RM7102	0.47 : 0.53	0.47 : 0.53	0.47 : 0.53	0.47 : 0.53
RM3533	0.55 : 0.45	0.55 : 0.45	0.47 : 0.53	0.48 : 0.52
Average	0.53 : 0.47	0.53 : 0.47	0.53 : 0.47	0.53 : 0.47

Supplementary Table 3 Results of the neutrality test, showing loci (in bold) whose parental alleles are significantly deviated (*P* <0.05) from the theoretical values in F_2/F_3 crop-wild hybrid lineages with (GE) or without (non-GE) the *epsps* transgene; with (CM) or without (non-CM) the crop-parent markers; and with (WM) or without (non-WM) the wild-parent markers, using the ideal groups as a reference.

Primer	F_2 GE							F_2 non-GE						
name	Obs.F	Min F	Max F	Mean*	SE*	L95*	U95*	Obs. F	Min F	Max F	Mean*	SE*	L95*	U95*
RM84	0.66	0.50	0.99	0.84	0.03	0.50	0.99	0.55	0.50	0.99	0.83	0.03	0.50	0.99
RM575	0.51	0.50	0.99	0.84	0.03	0.50	0.99	**0.50**	**0.50**	**0.99**	**0.83**	**0.03**	**0.50**	**0.99**
RM572	**0.50**	**0.50**	**0.99**	**0.84**	**0.03**	**0.50**	**0.99**	0.51	0.50	0.99	0.82	0.03	0.50	0.99
RM9	0.54	0.50	0.99	0.83	0.03	0.50	0.99	0.51	0.50	0.98	0.81	0.03	0.50	0.98
RM5	0.51	0.50	0.99	0.85	0.03	0.50	0.99	0.55	0.50	0.99	0.82	0.03	0.50	0.99
RM212	**0.50**	**0.50**	**0.99**	**0.86**	**0.03**	**0.51**	**0.99**	0.52	0.50	0.99	0.83	0.03	0.50	0.99
RM6895	0.67	0.50	0.99	0.85	0.03	0.51	0.99	0.62	0.50	0.99	0.84	0.03	0.50	0.99
RM7245	**0.50**	**0.50**	**0.99**	**0.85**	**0.03**	**0.50**	**0.99**	0.52	0.50	0.99	0.82	0.03	0.50	0.99
RM523	**0.50**	**0.50**	**0.99**	**0.84**	**0.03**	**0.50**	**0.99**	0.50	0.50	0.99	0.82	0.03	0.50	0.99
RM282	0.52	0.50	0.99	0.83	0.03	0.50	0.99	0.52	0.50	0.99	0.83	0.03	0.50	0.99
RM16	0.52	0.50	0.99	0.85	0.03	0.50	0.99	0.52	0.50	0.99	0.83	0.03	0.50	0.99
RM186	**0.50**	**0.50**	**0.99**	**0.84**	**0.03**	**0.50**	**0.99**	**0.50**	**0.50**	**0.99**	**0.82**	**0.03**	**0.50**	**0.99**
RM468	**0.50**	**0.50**	**0.99**	**0.84**	**0.03**	**0.50**	**0.99**	**0.50**	**0.50**	**0.99**	**0.83**	**0.03**	**0.50**	**0.99**
RM6395	0.63	0.50	0.99	0.84	0.03	0.50	0.99	0.50	0.50	0.99	0.82	0.03	0.50	0.99
RM6089	0.53	0.50	0.99	0.85	0.03	0.50	0.99	**0.50**	**0.50**	**0.99**	**0.84**	**0.03**	**0.50**	**0.99**
RM3558	0.53	0.50	0.99	0.84	0.03	0.50	0.99	0.52	0.50	0.99	0.84	0.03	0.50	0.99
RM6748	0.51	0.50	0.99	0.85	0.03	0.50	0.99	**0.50**	**0.50**	**0.99**	**0.84**	**0.03**	**0.50**	**0.99**
RM3419	0.51	0.50	0.99	0.84	0.03	0.50	0.99	0.54	0.50	0.99	0.83	0.03	0.50	0.99
RM6841	0.78	0.50	0.99	0.85	0.03	0.50	0.99	0.75	0.50	0.99	0.84	0.03	0.50	0.99
RM190	0.54	0.50	0.99	0.84	0.03	0.50	0.99	0.57	0.50	0.99	0.83	0.03	0.50	0.99

RM276	0.63	0.50	0.99	0.85	0.03	0.50	0.99	**0.50**	**0.50**	**0.99**	**0.83**	**0.03**	**0.50**	**0.99**
RM214	**0.50**	**0.50**	**0.99**	**0.85**	**0.03**	**0.50**	**0.99**	**0.50**	**0.50**	**0.99**	**0.84**	**0.03**	**0.50**	**0.99**
RM11	**0.50**	**0.50**	**0.99**	**0.84**	**0.03**	**0.50**	**0.99**	0.52	0.50	0.99	0.83	0.03	0.50	0.99
RM505	0.52	0.50	0.99	0.85	0.03	0.50	0.99	0.54	0.50	0.99	0.83	0.03	0.50	0.99
RM172	**0.50**	**0.50**	**0.99**	**0.85**	**0.03**	**0.50**	**0.99**	0.51	0.50	0.99	0.83	0.03	0.50	0.99
RM408	0.52	0.50	0.99	0.84	0.03	0.50	0.99	0.54	0.50	0.99	0.84	0.03	0.50	0.99
RM5068	0.69	0.50	0.99	0.85	0.03	0.50	0.99	0.71	0.50	0.99	0.84	0.03	0.50	0.99
RM331	0.65	0.50	0.99	0.84	0.03	0.50	0.99	0.63	0.50	0.99	0.84	0.03	0.50	0.99
RM296	0.63	0.50	0.99	0.85	0.03	0.51	0.99	0.61	0.50	0.99	0.82	0.03	0.50	0.99
RM4455	0.54	0.50	0.99	0.83	0.03	0.50	0.99	**0.50**	**0.50**	**0.99**	**0.82**	**0.03**	**0.50**	**0.99**
RM7557	0.54	0.50	0.99	0.83	0.03	0.50	0.99	0.52	0.50	0.99	0.81	0.03	0.50	0.99
RM332	0.51	0.50	0.99	0.85	0.03	0.50	0.99	0.51	0.50	0.99	0.83	0.03	0.50	0.99
RM4862	0.71	0.50	0.99	0.85	0.03	0.50	0.99	0.64	0.50	0.99	0.83	0.03	0.50	0.99
RM224	**0.50**	**0.50**	**0.99**	**0.84**	**0.03**	**0.50**	**0.99**	0.51	0.50	0.99	0.82	0.03	0.50	0.99
RM8216	0.51	0.50	0.99	0.84	0.03	0.50	0.99	**0.50**	**0.50**	**0.99**	**0.82**	**0.03**	**0.50**	**0.99**
RM3483	0.62	0.50	0.99	0.84	0.03	0.51	0.99	0.63	0.50	0.99	0.83	0.03	0.50	0.99
RM6296	0.51	0.50	0.99	0.85	0.03	0.51	0.99	0.51	0.50	0.99	0.84	0.03	0.51	0.99
RM277	**0.50**	**0.50**	**0.99**	**0.83**	**0.03**	**0.50**	**0.99**	**0.50**	**0.50**	**0.98**	**0.82**	**0.03**	**0.50**	**0.98**
RM6732	0.54	0.50	0.99	0.85	0.03	0.50	0.99	0.63	0.50	0.99	0.83	0.03	0.50	0.99
RM17	0.57	0.50	0.99	0.84	0.03	0.50	0.99	0.58	0.50	0.99	0.83	0.03	0.50	0.99
RM111	0.58	0.50	0.99	0.84	0.03	0.50	0.99	0.57	0.50	0.98	0.81	0.03	0.50	0.98
RM143	**0.50**	**0.50**	**0.99**	**0.85**	**0.03**	**0.51**	**0.99**	**0.50**	**0.50**	**0.99**	**0.83**	**0.03**	**0.50**	**0.99**
RM333	0.73	0.50	0.99	0.84	0.03	0.50	0.99	0.53	0.50	0.99	0.83	0.03	0.50	0.99
RM4128	0.53	0.50	0.99	0.84	0.03	0.50	0.99	0.51	0.50	0.99	0.83	0.03	0.50	0.99
RM168	0.51	0.50	0.99	0.84	0.03	0.50	0.99	0.51	0.50	0.99	0.83	0.03	0.50	0.99
RM565	0.52	0.50	0.99	0.85	0.03	0.50	0.99	0.57	0.50	0.99	0.83	0.03	0.50	0.99

RM16074	**0.50**	**0.50**	**0.99**	**0.85**	**0.03**	**0.50**	**0.99**	**0.50**	**0.50**	**0.99**	**0.83**	**0.03**	**0.50**	**0.99**
RM20468	0.52	0.50	0.99	0.84	0.03	0.51	0.99	0.52	0.50	0.99	0.83	0.03	0.50	0.99
RM130	0.52	0.50	0.99	0.85	0.03	0.51	0.99	0.52	0.50	0.99	0.83	0.03	0.50	0.99
RM20460	0.52	0.50	0.99	0.85	0.03	0.50	0.99	0.53	0.50	0.99	0.83	0.03	0.50	0.99
RM5371	0.63	0.50	0.99	0.84	0.03	0.50	0.99	**0.50**	**0.50**	**0.99**	**0.83**	**0.03**	**0.50**	**0.99**
RM16071	0.56	0.50	0.99	0.84	0.03	0.51	0.99	0.55	0.50	0.98	0.82	0.03	0.50	0.98

	F_2 CM-RM572							F_2 non-CM-RM572						
	Obs.F	Min F	Max F	Mean*	SE*	L95*	U95*	Obs. F	Min F	Max F	Mean*	SE*	L95*	U95*
RM84	0.52	0.50	1.00	0.85	0.03	0.50	1.00	0.58	0.50	1.00	0.85	0.03	0.50	1.00
RM575	0.51	0.50	1.00	0.86	0.03	0.50	1.00	0.56	0.50	1.00	0.84	0.03	0.50	1.00
RM572	-	-	-	-	-	-	-	-	-	-	-	-	-	-
RM9	**0.50**	**0.50**	**1.00**	**0.86**	**0.03**	**0.50**	**1.00**	0.53	0.50	1.00	0.85	0.03	0.50	1.00
RM5	**0.50**	**0.50**	**1.00**	**0.87**	**0.03**	**0.51**	**1.00**	0.51	0.50	0.99	0.84	0.03	0.50	0.99
RM212	0.51	0.50	1.00	0.85	0.03	0.50	1.00	0.53	0.50	0.99	0.84	0.03	0.50	0.99
RM6895	0.56	0.50	1.00	0.86	0.03	0.50	1.00	0.53	0.50	1.00	0.84	0.03	0.50	1.00
RM7245	0.51	0.50	1.00	0.87	0.03	0.50	1.00	0.51	0.50	1.00	0.85	0.03	0.50	1.00
RM523	0.53	0.50	1.00	0.87	0.03	0.51	1.00	0.53	0.50	1.00	0.84	0.03	0.50	1.00
RM282	0.59	0.50	1.00	0.86	0.03	0.50	1.00	0.61	0.50	1.00	0.84	0.03	0.50	1.00
RM16	0.53	0.50	1.00	0.86	0.03	0.50	1.00	0.55	0.50	1.00	0.85	0.03	0.50	1.00
RM186	0.58	0.50	1.00	0.85	0.03	0.50	1.00	0.59	0.50	1.00	0.85	0.03	0.50	1.00
RM468	0.56	0.50	1.00	0.86	0.03	0.50	1.00	0.56	0.50	1.00	0.85	0.03	0.50	1.00
RM6395	0.56	0.50	1.00	0.86	0.03	0.51	1.00	0.51	0.50	1.00	0.85	0.03	0.50	1.00
RM6089	0.51	0.50	1.00	0.85	0.03	0.50	1.00	**0.50**	**0.50**	**1.00**	**0.84**	**0.03**	**0.50**	**1.00**
RM3558	0.51	0.50	1.00	0.86	0.03	0.50	1.00	0.51	0.50	1.00	0.84	0.03	0.51	1.00
RM6748	**0.50**	**0.50**	**1.00**	**0.86**	**0.03**	**0.51**	**1.00**	0.50	0.50	1.00	0.85	0.03	0.50	1.00

RM3419	**0.50**	**0.50**	**1.00**	**0.86**	**0.03**	**0.50**	**1.00**	0.51	0.50	1.00	0.85	0.03	0.51	1.00
RM6841	0.59	0.50	1.00	0.86	0.03	0.50	1.00	0.60	0.50	1.00	0.85	0.03	0.50	1.00
RM190	0.61	0.50	1.00	0.87	0.03	0.51	1.00	0.59	0.50	1.00	0.84	0.03	0.50	1.00
RM276	**0.50**	**0.50**	**1.00**	**0.86**	**0.03**	**0.50**	**1.00**	**0.50**	**0.50**	**0.99**	**0.84**	**0.03**	**0.50**	**0.99**
RM214	0.58	0.50	1.00	0.86	0.03	0.50	1.00	0.60	0.50	1.00	0.86	0.03	0.50	1.00
RM11	**0.51**	**0.50**	**1.00**	**0.86**	**0.03**	**0.51**	**1.00**	**0.50**	**0.50**	**0.99**	**0.84**	**0.03**	**0.50**	**0.99**
RM505	0.56	0.50	1.00	0.86	0.03	0.51	1.00	0.53	0.50	0.99	0.84	0.03	0.50	0.99
RM172	**0.50**	**0.50**	**1.00**	**0.86**	**0.03**	**0.50**	**1.00**	**0.50**	**0.50**	**1.00**	**0.85**	**0.03**	**0.51**	**1.00**
RM408	**0.50**	**0.50**	**1.00**	**0.86**	**0.03**	**0.50**	**1.00**	**0.50**	**0.50**	**1.00**	**0.85**	**0.03**	**0.50**	**1.00**
RM5068	0.63	0.50	1.00	0.86	0.03	0.50	1.00	0.71	0.50	1.00	0.85	0.03	0.50	1.00
RM331	0.54	0.50	1.00	0.86	0.03	0.50	1.00	0.51	0.50	1.00	0.86	0.03	0.50	1.00
RM296	0.52	0.50	1.00	0.87	0.03	0.51	1.00	0.51	0.50	1.00	0.86	0.03	0.50	1.00
RM4455	0.52	0.50	1.00	0.87	0.03	0.50	1.00	0.54	0.50	1.00	0.85	0.03	0.50	1.00
RM7557	0.51	0.50	1.00	0.86	0.03	0.50	1.00	0.50	0.50	0.99	0.85	0.03	0.50	0.99
RM332	0.52	0.50	1.00	0.86	0.03	0.50	1.00	0.51	0.50	0.99	0.85	0.03	0.50	0.99
RM4862	0.68	0.50	1.00	0.87	0.03	0.51	1.00	0.65	0.50	1.00	0.85	0.03	0.51	1.00
RM224	0.52	0.50	1.00	0.86	0.03	0.51	1.00	0.50	0.50	1.00	0.85	0.03	0.50	1.00
RM8216	0.55	0.50	1.00	0.85	0.03	0.50	1.00	0.55	0.50	1.00	0.84	0.03	0.50	1.00
RM3483	0.51	0.50	1.00	0.86	0.03	0.50	1.00	0.52	0.50	0.99	0.85	0.03	0.50	0.99
RM6296	0.55	0.50	1.00	0.87	0.03	0.51	1.00	0.58	0.50	1.00	0.84	0.03	0.50	1.00
RM277	**0.51**	**0.50**	**1.00**	**0.86**	**0.03**	**0.51**	**1.00**	**0.50**	**0.50**	**0.99**	**0.85**	**0.03**	**0.50**	**0.99**
RM6732	0.55	0.50	1.00	0.86	0.03	0.50	1.00	0.55	0.50	1.00	0.85	0.03	0.50	1.00
RM17	0.50	0.50	1.00	0.87	0.03	0.50	1.00	0.50	0.50	0.99	0.84	0.03	0.50	0.99
RM111	0.52	0.50	1.00	0.86	0.03	0.50	1.00	0.55	0.50	0.99	0.84	0.03	0.50	0.99
RM143	**0.50**	**0.50**	**1.00**	**0.85**	**0.03**	**0.50**	**1.00**	0.50	0.33	0.99	0.73	0.04	0.40	0.99
RM333	0.50	0.50	1.00	0.86	0.03	0.50	1.00	0.51	0.50	0.99	0.85	0.03	0.50	0.99

RM4128	0.51	0.50	1.00	0.87	0.03	0.51	1.00	**0.50**	**0.50**	**0.99**	**0.85**	**0.03**	**0.50**	**0.99**
RM168	0.55	0.50	1.00	0.87	0.03	0.51	1.00	0.57	0.50	1.00	0.85	0.03	0.51	1.00
RM565	0.53	0.50	1.00	0.87	0.03	0.50	1.00	0.53	0.50	1.00	0.86	0.03	0.51	1.00
RM16074	**0.50**	**0.50**	**1.00**	**0.86**	**0.03**	**0.51**	**1.00**	**0.50**	**0.50**	**1.00**	**0.85**	**0.03**	**0.51**	**1.00**
RM20468	0.51	0.50	1.00	0.87	0.03	0.50	1.00	0.52	0.50	1.00	0.84	0.03	0.50	1.00
RM130	0.51	0.50	1.00	0.86	0.03	0.50	1.00	**0.50**	**0.50**	**1.00**	**0.86**	**0.03**	**0.51**	**1.00**
RM20460	0.51	0.50	1.00	0.86	0.03	0.50	1.00	0.52	0.50	1.00	0.85	0.03	0.50	1.00
RM5371	0.52	0.50	1.00	0.86	0.03	0.50	1.00	0.54	0.50	1.00	0.84	0.03	0.50	1.00
RM16071	0.51	0.50	1.00	0.86	0.03	0.50	1.00	0.50	0.50	0.99	0.84	0.03	0.50	0.99

	F_2 WM-RM572							F_2 non-WM-RM572						
	Obs.F	Min F	Max F	Mean*	SE*	L95*	U95*	Obs. F	Min F	Max F	Mean*	SE*	L95*	U95*
RM84	0.54	0.50	1.00	0.86	0.03	0.51	1.00	0.53	0.50	1.00	0.86	0.03	0.51	1.00
RM575	0.52	0.50	1.00	0.87	0.03	0.51	1.00	0.51	0.50	1.00	0.85	0.03	0.50	1.00
RM572	-	-	-	-	-	-	-	-	-	-	-	-	-	-
RM9	0.51	0.50	1.00	0.86	0.03	0.51	1.00	0.51	0.50	1.00	0.84	0.03	0.50	1.00
RM5	**0.50**	**0.50**	**1.00**	**0.86**	**0.03**	**0.51**	**1.00**	**0.50**	**0.50**	**1.00**	**0.85**	**0.03**	**0.51**	**1.00**
RM212	0.52	0.50	1.00	0.85	0.03	0.50	1.00	0.51	0.50	1.00	0.85	0.03	0.50	1.00
RM6895	0.53	0.50	1.00	0.86	0.03	0.51	1.00	0.57	0.50	1.00	0.86	0.03	0.50	1.00
RM7245	0.51	0.50	1.00	0.86	0.03	0.50	1.00	0.51	0.50	1.00	0.85	0.03	0.51	1.00
RM523	0.54	0.50	1.00	0.85	0.03	0.50	1.00	0.52	0.50	1.00	0.85	0.03	0.50	1.00
RM282	0.61	0.50	1.00	0.86	0.03	0.51	1.00	0.58	0.50	1.00	0.86	0.03	0.50	1.00
RM16	0.55	0.50	1.00	0.86	0.03	0.50	1.00	0.51	0.50	1.00	0.85	0.03	0.50	1.00
RM186	0.59	0.50	1.00	0.86	0.03	0.51	1.00	0.58	0.50	1.00	0.85	0.03	0.50	1.00
RM468	0.57	0.50	1.00	0.85	0.03	0.50	1.00	0.55	0.50	1.00	0.86	0.03	0.50	1.00
RM6395	0.53	0.50	1.00	0.86	0.03	0.50	1.00	0.55	0.50	1.00	0.85	0.03	0.50	1.00

RM6089	**0.50**	**0.50**	**1.00**	**0.86**	**0.03**	**0.50**	**1.00**	0.51	0.50	1.00	0.85	0.03	0.50	1.00
RM3558	0.51	0.50	1.00	0.86	0.03	0.50	1.00	0.51	0.50	1.00	0.86	0.03	0.50	1.00
RM6748	**0.50**	**0.50**	**1.00**	**0.87**	**0.03**	**0.51**	**1.00**	**0.50**	**0.50**	**1.00**	**0.86**	**0.03**	**0.51**	**1.00**
RM3419	0.51	0.50	1.00	0.86	0.03	0.50	1.00	**0.50**	**0.50**	**1.00**	**0.85**	**0.03**	**0.50**	**1.00**
RM6841	0.61	0.50	1.00	0.86	0.03	0.50	1.00	0.58	0.50	1.00	0.86	0.03	0.51	1.00
RM190	0.59	0.50	1.00	0.87	0.03	0.51	1.00	0.61	0.50	1.00	0.86	0.03	0.50	1.00
RM276	**0.50**	**0.50**	**1.00**	**0.86**	**0.03**	**0.50**	**1.00**	**0.50**	**0.50**	**1.00**	**0.84**	**0.03**	**0.50**	**1.00**
RM214	0.61	0.50	1.00	0.86	0.03	0.50	1.00	0.55	0.50	1.00	0.85	0.03	0.50	1.00
RM11	0.51	0.50	1.00	0.85	0.03	0.50	1.00	0.50	0.50	1.00	0.85	0.03	0.50	1.00
RM505	0.54	0.50	1.00	0.86	0.03	0.51	1.00	0.57	0.50	1.00	0.84	0.03	0.50	1.00
RM172	**0.50**	**0.50**	**1.00**	**0.86**	**0.03**	**0.51**	**1.00**	**0.50**	**0.50**	**1.00**	**0.85**	**0.03**	**0.51**	**1.00**
RM408	**0.50**	**0.50**	**1.00**	**0.86**	**0.03**	**0.50**	**1.00**	**0.50**	**0.50**	**1.00**	**0.85**	**0.03**	**0.50**	**1.00**
RM5068	0.69	0.50	1.00	0.86	0.03	0.51	1.00	0.62	0.50	1.00	0.86	0.03	0.50	1.00
RM331	0.52	0.50	1.00	0.85	0.03	0.50	1.00	0.54	0.50	1.00	0.85	0.03	0.50	1.00
RM296	0.51	0.50	1.00	0.86	0.03	0.50	1.00	0.52	0.50	1.00	0.85	0.03	0.50	1.00
RM4455	0.54	0.50	1.00	0.86	0.03	0.50	1.00	0.51	0.50	1.00	0.85	0.03	0.50	1.00
RM7557	**0.50**	**0.50**	**1.00**	**0.85**	**0.03**	**0.50**	**1.00**	0.51	0.50	1.00	0.86	0.03	0.51	1.00
RM332	0.51	0.50	1.00	0.86	0.03	0.51	1.00	0.53	0.50	1.00	0.85	0.03	0.50	1.00
RM4862	0.67	0.50	1.00	0.87	0.03	0.51	1.00	0.66	0.50	1.00	0.86	0.03	0.51	1.00
RM224	0.51	0.50	1.00	0.87	0.03	0.50	1.00	0.53	0.50	1.00	0.85	0.03	0.50	1.00
RM8216	0.55	0.50	1.00	0.86	0.03	0.50	1.00	0.55	0.50	1.00	0.86	0.03	0.51	1.00
RM3483	0.52	0.50	1.00	0.86	0.03	0.50	1.00	0.51	0.50	1.00	0.85	0.03	0.50	1.00
RM6296	0.57	0.50	1.00	0.86	0.03	0.50	1.00	0.55	0.50	1.00	0.85	0.03	0.50	1.00
RM277	**0.50**	**0.50**	**1.00**	**0.87**	**0.03**	**0.50**	**1.00**	0.50	0.50	1.00	0.85	0.03	0.50	1.00
RM6732	0.55	0.50	1.00	0.86	0.03	0.50	1.00	0.55	0.50	1.00	0.85	0.03	0.50	1.00
RM17	0.50	0.50	1.00	0.85	0.03	0.50	1.00	**0.50**	**0.50**	**1.00**	**0.85**	**0.03**	**0.51**	**1.00**

RM111	0.54	0.50	1.00	0.85	0.03	0.50	1.00	0.51	0.50	1.00	0.85	0.03	0.50	1.00
RM143	0.50	0.50	0.99	0.75	0.04	0.41	0.99	0.50	0.50	1.00	0.86	0.03	0.50	1.00
RM333	**0.50**	**0.50**	**1.00**	**0.86**	**0.03**	**0.51**	**1.00**	**0.50**	**0.50**	**1.00**	**0.85**	**0.03**	**0.51**	**1.00**
RM4128	**0.51**	**0.50**	**1.00**	**0.86**	**0.03**	**0.51**	**1.00**	0.52	0.50	1.00	0.85	0.03	0.50	1.00
RM168	0.57	0.50	1.00	0.86	0.03	0.50	1.00	0.54	0.50	1.00	0.86	0.03	0.50	1.00
RM565	0.53	0.50	1.00	0.86	0.03	0.51	1.00	0.53	0.50	1.00	0.85	0.03	0.51	1.00
RM16074	**0.50**	**0.50**	**1.00**	**0.86**	**0.03**	**0.50**	**1.00**	**0.50**	**0.50**	**1.00**	**0.85**	**0.03**	**0.50**	**1.00**
RM20468	0.51	0.50	1.00	0.85	0.03	0.50	1.00	0.51	0.50	1.00	0.85	0.03	0.50	1.00
RM130	0.50	0.50	1.00	0.86	0.03	0.50	1.00	**0.50**	**0.50**	**1.00**	**0.86**	**0.03**	**0.51**	**1.00**
RM20460	0.51	0.50	1.00	0.85	0.03	0.50	1.00	0.51	0.50	1.00	0.85	0.03	0.50	1.00
RM5371	0.53	0.50	1.00	0.86	0.03	0.51	1.00	0.52	0.50	1.00	0.85	0.03	0.51	1.00
RM16071	**0.50**	**0.50**	**1.00**	**0.87**	**0.02**	**0.51**	**1.00**	**0.50**	**0.50**	**1.00**	**0.85**	**0.03**	**0.51**	**1.00**

	F_2 CM-RM408							F_2 non-CM-RM408						
	Obs.F	Min F	Max F	Mean*	SE*	L95*	U95*	Obs. F	Min F	Max F	Mean*	SE*	L95*	U95*
RM84	0.53	0.50	1.00	0.87	0.03	0.50	1.00	0.57	0.50	0.99	0.85	0.03	0.50	0.99
RM575	**0.50**	**0.50**	**1.00**	**0.87**	**0.03**	**0.51**	**1.00**	0.51	0.50	0.99	0.84	0.03	0.50	0.99
RM572	**0.50**	**0.50**	**1.00**	**0.86**	**0.03**	**0.51**	**1.00**	**0.50**	**0.50**	**0.99**	**0.85**	**0.03**	**0.51**	**0.99**
RM9	0.50	0.50	1.00	0.86	0.03	0.50	1.00	0.52	0.50	0.99	0.84	0.03	0.50	0.99
RM5	**0.50**	**0.50**	**1.00**	**0.87**	**0.03**	**0.51**	**1.00**	0.51	0.50	0.99	0.84	0.03	0.50	0.99
RM212	0.51	0.50	1.00	0.86	0.03	0.50	1.00	0.53	0.50	0.99	0.85	0.03	0.50	0.99
RM6895	0.54	0.50	1.00	0.86	0.03	0.50	1.00	0.55	0.50	0.99	0.84	0.03	0.50	0.99
RM7245	0.51	0.50	1.00	0.86	0.03	0.50	1.00	0.51	0.50	0.99	0.85	0.03	0.50	0.99
RM523	0.53	0.50	1.00	0.85	0.03	0.50	1.00	0.54	0.50	0.99	0.84	0.03	0.50	0.99
RM282	0.58	0.50	1.00	0.86	0.03	0.50	1.00	0.63	0.50	0.99	0.85	0.03	0.51	0.99
RM16	0.53	0.50	1.00	0.86	0.03	0.50	1.00	0.54	0.50	0.99	0.85	0.03	0.50	0.99

RM186	0.57	0.50	1.00	0.86	0.03	0.50	1.00	0.61	0.50	0.99	0.85	0.03	0.50	0.99
RM468	0.56	0.50	1.00	0.87	0.03	0.51	1.00	0.57	0.50	0.99	0.84	0.03	0.50	0.99
RM6395	0.53	0.50	1.00	0.86	0.03	0.50	1.00	0.54	0.50	0.99	0.84	0.03	0.50	0.99
RM6089	**0.50**	**0.50**	**1.00**	**0.86**	**0.03**	**0.50**	**1.00**	**0.50**	**0.50**	**0.99**	**0.85**	**0.03**	**0.50**	**0.99**
RM3558	0.51	0.50	1.00	0.87	0.03	0.51	1.00	0.53	0.50	0.99	0.85	0.03	0.50	0.99
RM6748	**0.50**	**0.50**	**1.00**	**0.87**	**0.02**	**0.51**	**1.00**	0.50	0.50	0.99	0.84	0.03	0.50	0.99
RM3419	0.51	0.50	1.00	0.86	0.03	0.50	1.00	**0.50**	**0.50**	**0.99**	**0.84**	**0.03**	**0.50**	**0.99**
RM6841	0.58	0.50	1.00	0.86	0.03	0.50	1.00	0.63	0.50	0.99	0.84	0.03	0.50	0.99
RM190	0.58	0.50	1.00	0.86	0.03	0.51	1.00	0.65	0.50	0.99	0.85	0.03	0.50	0.99
RM276	**0.50**	**0.50**	**1.00**	**0.86**	**0.03**	**0.50**	**1.00**	0.52	0.50	0.99	0.85	0.03	0.50	0.99
RM214	0.57	0.50	1.00	0.86	0.03	0.51	1.00	0.61	0.50	0.99	0.84	0.03	0.50	0.99
RM11	0.51	0.50	1.00	0.86	0.03	0.50	1.00	**0.50**	**0.50**	**0.99**	**0.85**	**0.03**	**0.50**	**0.99**
RM505	0.55	0.50	1.00	0.85	0.03	0.50	1.00	0.54	0.50	0.99	0.85	0.03	0.50	0.99
RM172	**0.50**	**0.50**	**1.00**	**0.86**	**0.03**	**0.51**	**1.00**	0.51	0.50	0.99	0.84	0.03	0.50	0.99
RM408	-	-	-	-	-	-	-	-	-	-	-	-	-	-
RM5068	0.63	0.50	1.00	0.86	0.03	0.50	1.00	0.74	0.50	0.99	0.85	0.03	0.50	0.99
RM331	0.52	0.50	1.00	0.86	0.03	0.50	1.00	0.53	0.50	0.99	0.85	0.03	0.51	0.99
RM296	0.52	0.50	1.00	0.86	0.03	0.50	1.00	0.52	0.50	0.99	0.85	0.03	0.51	0.99
RM4455	0.53	0.50	1.00	0.86	0.03	0.50	1.00	0.51	0.50	0.99	0.84	0.03	0.50	0.99
RM7557	**0.50**	**0.50**	**1.00**	**0.86**	**0.03**	**0.50**	**1.00**	0.52	0.50	0.99	0.85	0.03	0.51	0.99
RM332	0.53	0.50	1.00	0.86	0.03	0.50	1.00	0.51	0.50	0.99	0.85	0.03	0.50	0.99
RM4862	0.69	0.50	1.00	0.87	0.02	0.51	1.00	0.62	0.50	0.99	0.84	0.03	0.50	0.99
RM224	0.51	0.50	1.00	0.85	0.03	0.51	1.00	0.52	0.50	0.99	0.84	0.03	0.50	0.99
RM8216	0.58	0.50	1.00	0.87	0.03	0.51	1.00	0.51	0.50	0.99	0.84	0.03	0.50	0.99
RM3483	0.52	0.50	1.00	0.87	0.03	0.51	1.00	**0.50**	**0.50**	**0.99**	**0.84**	**0.03**	**0.50**	**0.99**
RM6296	0.57	0.50	1.00	0.86	0.03	0.50	1.00	0.54	0.50	0.99	0.84	0.03	0.50	0.99

RM277	0.51	0.50	1.00	0.87	0.03	0.51	1.00	**0.50**	**0.50**	**0.99**	**0.85**	**0.03**	**0.50**	**0.99**
RM6732	0.54	0.50	1.00	0.86	0.03	0.51	1.00	0.57	0.50	0.99	0.85	0.03	0.50	0.99
RM17	0.51	0.50	1.00	0.86	0.03	0.50	1.00	**0.50**	**0.50**	**0.99**	**0.85**	**0.03**	**0.50**	**0.99**
RM111	0.54	0.50	1.00	0.86	0.03	0.51	1.00	**0.50**	**0.50**	**0.99**	**0.84**	**0.03**	**0.50**	**0.99**
RM143	0.50	0.33	0.99	0.75	0.04	0.40	0.99	**0.50**	**0.50**	**0.99**	**0.84**	**0.03**	**0.50**	**0.99**
RM333	**0.50**	**0.50**	**1.00**	**0.86**	**0.03**	**0.50**	**1.00**	**0.50**	**0.50**	**0.99**	**0.84**	**0.03**	**0.50**	**0.99**
RM4128	0.51	0.50	1.00	0.87	0.03	0.50	1.00	0.52	0.50	0.99	0.85	0.03	0.50	0.99
RM168	0.54	0.50	1.00	0.86	0.03	0.50	1.00	0.61	0.50	0.99	0.85	0.03	0.51	0.99
RM565	0.53	0.50	1.00	0.86	0.03	0.51	1.00	0.53	0.50	0.99	0.84	0.03	0.50	0.99
RM16074	**0.50**	**0.50**	**1.00**	**0.86**	**0.03**	**0.50**	**1.00**	**0.50**	**0.50**	**0.99**	**0.84**	**0.03**	**0.50**	**0.99**
RM20468	0.51	0.50	1.00	0.85	0.03	0.50	1.00	0.51	0.50	0.99	0.84	0.03	0.50	0.99
RM130	**0.50**	**0.50**	**1.00**	**0.86**	**0.03**	**0.51**	**1.00**	0.51	0.50	0.99	0.85	0.03	0.50	0.99
RM20460	0.51	0.50	1.00	0.86	0.03	0.50	1.00	0.51	0.50	0.99	0.85	0.03	0.50	0.99
RM5371	0.52	0.50	1.00	0.86	0.03	0.51	1.00	0.52	0.50	0.99	0.84	0.03	0.50	0.99
RM16071	0.50	0.50	1.00	0.87	0.03	0.50	1.00	0.50	0.50	0.99	0.85	0.03	0.50	0.99

	F_2 WM-RM408							F_2 non-WM-RM408						
	Obs.F	Min F	Max F	Mean*	SE*	L95*	U95*	Obs. F	Min F	Max F	Mean*	SE*	L95*	U95*
RM84	0.55	0.50	1.00	0.86	0.03	0.50	1.00	0.52	0.50	0.99	0.84	0.03	0.50	0.99
RM575	**0.50**	**0.50**	**1.00**	**0.86**	**0.03**	**0.50**	**1.00**	**0.50**	**0.50**	**0.99**	**0.84**	**0.03**	**0.50**	**0.99**
RM572	**0.50**	**0.50**	**1.00**	**0.87**	**0.03**	**0.51**	**1.00**	**0.50**	**0.50**	**0.99**	**0.85**	**0.03**	**0.50**	**0.99**
RM9	0.52	0.50	1.00	0.87	0.03	0.51	1.00	**0.50**	**0.50**	**0.99**	**0.85**	**0.03**	**0.50**	**0.99**
RM5	**0.50**	**0.50**	**1.00**	**0.86**	**0.03**	**0.51**	**1.00**	**0.50**	**0.50**	**0.99**	**0.85**	**0.03**	**0.50**	**0.99**
RM212	0.52	0.50	1.00	0.87	0.02	0.51	1.00	0.51	0.50	0.99	0.84	0.03	0.50	0.99
RM6895	0.55	0.50	1.00	0.86	0.03	0.50	1.00	0.54	0.50	0.99	0.85	0.03	0.50	0.99
RM7245	0.51	0.50	1.00	0.87	0.03	0.50	1.00	0.51	0.50	0.99	0.85	0.03	0.50	0.99

RM523	0.53	0.50	1.00	0.85	0.03	0.50	1.00	0.52	0.50	0.99	0.85	0.03	0.51	0.99
RM282	0.59	0.50	1.00	0.86	0.03	0.50	1.00	0.60	0.50	0.99	0.85	0.03	0.50	0.99
RM16	0.53	0.50	1.00	0.86	0.03	0.51	1.00	0.54	0.50	0.99	0.86	0.03	0.51	0.99
RM186	0.60	0.50	1.00	0.86	0.03	0.51	1.00	0.55	0.50	0.99	0.85	0.03	0.50	0.99
RM468	0.57	0.50	1.00	0.86	0.03	0.50	1.00	0.54	0.50	0.99	0.84	0.03	0.50	0.99
RM6395	0.54	0.50	1.00	0.86	0.03	0.50	1.00	0.53	0.50	0.99	0.84	0.03	0.50	0.99
RM6089	**0.50**	**0.50**	**1.00**	**0.87**	**0.03**	**0.50**	**1.00**	0.50	0.50	0.99	0.84	0.03	0.50	0.99
RM3558	0.51	0.50	1.00	0.86	0.03	0.50	1.00	0.51	0.50	0.99	0.84	0.03	0.50	0.99
RM6748	**0.50**	**0.50**	**1.00**	**0.86**	**0.03**	**0.50**	**1.00**	**0.50**	**0.50**	**0.99**	**0.86**	**0.03**	**0.50**	**0.99**
RM3419	**0.50**	**0.50**	**1.00**	**0.86**	**0.03**	**0.51**	**1.00**	0.52	0.50	0.99	0.85	0.03	0.50	0.99
RM6841	0.61	0.50	1.00	0.86	0.03	0.50	1.00	0.57	0.50	0.99	0.85	0.03	0.50	0.99
RM190	0.61	0.50	1.00	0.86	0.03	0.50	1.00	0.59	0.50	0.99	0.84	0.03	0.50	0.99
RM276	**0.50**	**0.50**	**1.00**	**0.87**	**0.03**	**0.50**	**1.00**	**0.50**	**0.50**	**0.99**	**0.84**	**0.03**	**0.50**	**0.99**
RM214	0.60	0.50	1.00	0.87	0.03	0.50	1.00	0.55	0.50	0.99	0.84	0.03	0.50	0.99
RM11	**0.50**	**0.50**	**1.00**	**0.85**	**0.03**	**0.50**	**1.00**	0.53	0.50	0.99	0.85	0.03	0.51	0.99
RM505	0.54	0.50	1.00	0.86	0.03	0.50	1.00	0.57	0.50	0.99	0.84	0.03	0.50	0.99
RM172	**0.50**	**0.50**	**1.00**	**0.85**	**0.03**	**0.51**	**1.00**	**0.50**	**0.50**	**0.99**	**0.85**	**0.03**	**0.50**	**0.99**
RM408	-	-	-	-	-	-	-	-	-	-	-	-	-	-
RM5068	0.74	0.50	1.00	0.87	0.03	0.50	1.00	0.53	0.50	0.99	0.83	0.03	0.50	0.99
RM331	0.53	0.50	1.00	0.86	0.03	0.50	1.00	0.52	0.50	0.99	0.84	0.03	0.50	0.99
RM296	0.51	0.50	1.00	0.86	0.03	0.50	1.00	0.54	0.50	0.99	0.83	0.03	0.50	0.99
RM4455	0.53	0.50	1.00	0.85	0.03	0.51	1.00	0.52	0.50	0.99	0.84	0.03	0.50	0.99
RM7557	**0.51**	**0.50**	**1.00**	**0.86**	**0.03**	**0.51**	**1.00**	0.50	0.50	0.99	0.85	0.03	0.50	0.99
RM332	0.51	0.50	1.00	0.86	0.03	0.50	1.00	0.53	0.50	0.99	0.84	0.03	0.50	0.99
RM4862	0.66	0.50	1.00	0.85	0.03	0.50	1.00	0.68	0.50	0.99	0.84	0.03	0.50	0.99
RM224	0.52	0.50	1.00	0.87	0.03	0.50	1.00	0.51	0.50	0.99	0.85	0.03	0.50	0.99

RM8216	0.55	0.50	1.00	0.86	0.03	0.51	1.00	0.57	0.50	0.99	0.85	0.03	0.51	0.99
RM3483	0.51	0.50	1.00	0.86	0.03	0.51	1.00	0.53	0.50	0.99	0.85	0.03	0.50	0.99
RM6296	0.55	0.50	1.00	0.86	0.03	0.51	1.00	0.59	0.50	0.99	0.85	0.03	0.51	0.99
RM277	**0.50**	**0.50**	**1.00**	**0.86**	**0.03**	**0.50**	**1.00**	0.54	0.50	0.99	0.84	0.03	0.50	0.99
RM6732	0.55	0.50	1.00	0.86	0.03	0.50	1.00	0.54	0.50	0.99	0.84	0.03	0.50	0.99
RM17	**0.50**	**0.50**	**1.00**	**0.87**	**0.03**	**0.51**	**1.00**	0.52	0.50	0.99	0.86	0.03	0.51	0.99
RM111	0.52	0.50	1.00	0.86	0.03	0.51	1.00	0.55	0.50	0.99	0.85	0.03	0.50	0.99
RM143	**0.50**	**0.50**	**1.00**	**0.86**	**0.03**	**0.51**	**1.00**	0.50	0.33	0.99	0.73	0.04	0.38	0.98
RM333	**0.50**	**0.50**	**1.00**	**0.87**	**0.03**	**0.50**	**1.00**	**0.50**	**0.50**	**0.99**	**0.84**	**0.03**	**0.50**	**0.99**
RM4128	0.51	0.50	1.00	0.86	0.03	0.51	1.00	0.50	0.50	0.99	0.85	0.03	0.50	0.99
RM168	0.57	0.50	1.00	0.86	0.03	0.51	1.00	0.53	0.50	0.99	0.84	0.03	0.50	0.99
RM565	0.54	0.50	1.00	0.86	0.03	0.50	1.00	0.52	0.50	0.99	0.84	0.03	0.50	0.99
RM16074	**0.50**	**0.50**	**1.00**	**0.86**	**0.03**	**0.51**	**1.00**	**0.50**	**0.50**	**0.99**	**0.85**	**0.03**	**0.50**	**0.99**
RM20468	0.51	0.50	1.00	0.87	0.03	0.51	1.00	0.51	0.50	0.99	0.84	0.03	0.50	0.99
RM130	0.51	0.50	1.00	0.87	0.03	0.50	1.00	**0.50**	**0.50**	**0.99**	**0.84**	**0.03**	**0.50**	**0.99**
RM20460	0.51	0.50	1.00	0.85	0.03	0.50	1.00	0.51	0.50	0.99	0.85	0.03	0.50	0.99
RM5371	0.53	0.50	1.00	0.86	0.03	0.50	1.00	0.51	0.50	0.99	0.84	0.03	0.50	0.99
RM16071	0.51	0.50	1.00	0.87	0.03	0.50	1.00	**0.50**	**0.50**	**0.99**	**0.85**	**0.03**	**0.50**	**0.99**

	F_2 CM-RM277							F_2 non-CM-RM277						
	Obs.F	Min F	Max F	Mean*	SE*	L95*	U95*	Obs. F	Min F	Max F	Mean*	SE*	L95*	U95*
RM84	0.53	0.50	1.00	0.86	0.03	0.51	1.00	0.56	0.50	0.99	0.84	0.03	0.50	0.99
RM575	**0.50**	**0.50**	**1.00**	**0.86**	**0.03**	**0.51**	**1.00**	0.51	0.50	0.99	0.84	0.03	0.50	0.99
RM572	**0.50**	**0.50**	**1.00**	**0.86**	**0.03**	**0.51**	**1.00**	**0.50**	**0.50**	**0.99**	**0.84**	**0.03**	**0.50**	**0.99**
RM9	0.50	0.50	1.00	0.87	0.03	0.50	1.00	0.52	0.50	0.99	0.84	0.03	0.50	0.99
RM5	**0.50**	**0.50**	**1.00**	**0.86**	**0.03**	**0.50**	**1.00**	0.52	0.50	0.99	0.84	0.03	0.51	0.99

RM212	0.52	0.50	1.00	0.87	0.03	0.51	1.00	0.51	0.50	0.99	0.84	0.03	0.50	0.99
RM6895	0.56	0.50	1.00	0.86	0.03	0.50	1.00	0.51	0.50	0.99	0.83	0.03	0.50	0.99
RM7245	0.51	0.50	1.00	0.86	0.03	0.51	1.00	0.51	0.50	0.99	0.85	0.03	0.50	0.99
RM523	0.54	0.50	1.00	0.86	0.03	0.50	1.00	0.52	0.50	0.99	0.84	0.03	0.50	0.99
RM282	0.60	0.50	1.00	0.87	0.03	0.50	1.00	0.59	0.50	0.99	0.83	0.03	0.50	0.99
RM16	0.53	0.50	1.00	0.86	0.03	0.50	1.00	0.54	0.50	0.99	0.83	0.03	0.50	0.99
RM186	0.58	0.50	1.00	0.86	0.03	0.50	1.00	0.59	0.50	0.99	0.83	0.03	0.50	0.99
RM468	0.56	0.50	1.00	0.86	0.03	0.51	1.00	0.56	0.50	0.99	0.83	0.03	0.50	0.99
RM6395	0.53	0.50	1.00	0.87	0.03	0.50	1.00	0.55	0.50	0.99	0.83	0.03	0.50	0.99
RM6089	**0.50**	**0.50**	**1.00**	**0.87**	**0.03**	**0.50**	**1.00**	0.51	0.50	0.99	0.84	0.03	0.50	0.99
RM3558	0.51	0.50	1.00	0.87	0.03	0.51	1.00	0.52	0.50	0.99	0.84	0.03	0.50	0.99
RM6748	**0.50**	**0.50**	**1.00**	**0.86**	**0.03**	**0.50**	**1.00**	**0.50**	**0.50**	**0.99**	**0.84**	**0.03**	**0.50**	**0.99**
RM3419	0.51	0.50	1.00	0.87	0.03	0.51	1.00	0.50	0.50	0.99	0.84	0.03	0.50	0.99
RM6841	0.61	0.50	1.00	0.86	0.03	0.51	1.00	0.56	0.50	0.99	0.83	0.03	0.50	0.99
RM190	0.59	0.50	1.00	0.86	0.03	0.51	1.00	0.63	0.50	0.99	0.84	0.03	0.50	0.99
RM276	**0.50**	**0.50**	**1.00**	**0.87**	**0.03**	**0.51**	**1.00**	0.52	0.50	0.99	0.83	0.03	0.50	0.99
RM214	0.58	0.50	1.00	0.88	0.03	0.50	1.00	0.60	0.50	0.99	0.84	0.03	0.50	0.99
RM11	0.51	0.50	1.00	0.87	0.03	0.50	1.00	**0.50**	**0.50**	**0.99**	**0.84**	**0.03**	**0.50**	**0.99**
RM505	0.55	0.50	1.00	0.86	0.03	0.50	1.00	0.54	0.50	0.99	0.85	0.03	0.50	0.99
RM172	**0.50**	**0.50**	**1.00**	**0.86**	**0.03**	**0.50**	**1.00**	0.51	0.50	0.99	0.83	0.03	0.50	0.99
RM408	**0.50**	**0.50**	**1.00**	**0.86**	**0.03**	**0.50**	**1.00**	0.52	0.50	0.99	0.84	0.03	0.50	0.99
RM5068	0.62	0.50	1.00	0.86	0.03	0.50	1.00	0.79	0.50	0.99	0.83	0.03	0.50	0.99
RM331	0.53	0.50	1.00	0.87	0.03	0.50	1.00	0.51	0.50	0.99	0.84	0.03	0.50	0.99
RM296	0.52	0.50	1.00	0.86	0.03	0.50	1.00	0.51	0.50	0.99	0.83	0.03	0.50	0.99
RM4455	0.52	0.50	1.00	0.87	0.02	0.51	1.00	0.53	0.50	0.99	0.84	0.03	0.50	0.99
RM7557	**0.50**	**0.50**	**1.00**	**0.87**	**0.03**	**0.50**	**1.00**	0.52	0.50	0.99	0.83	0.03	0.50	0.99

RM332	0.52	0.50	1.00	0.87	0.03	0.51	1.00	0.51	0.50	0.99	0.83	0.03	0.50	0.99
RM4862	0.68	0.50	1.00	0.87	0.03	0.51	1.00	0.64	0.50	0.99	0.84	0.03	0.51	0.99
RM224	0.51	0.50	1.00	0.86	0.03	0.50	1.00	0.53	0.50	0.99	0.83	0.03	0.50	0.99
RM8216	0.57	0.50	1.00	0.87	0.03	0.51	1.00	0.52	0.50	0.99	0.83	0.03	0.50	0.99
RM3483	0.52	0.50	1.00	0.87	0.03	0.50	1.00	**0.50**	**0.50**	**0.99**	**0.84**	**0.03**	**0.50**	**0.99**
RM6296	0.60	0.50	1.00	0.86	0.03	0.51	1.00	**0.50**	**0.50**	**0.99**	**0.84**	**0.03**	**0.50**	**0.99**
RM277	-	-	-	-	-	-	-	-	-	-	-	-	-	-
RM6732	0.56	0.50	1.00	0.87	0.03	0.51	1.00	0.52	0.50	0.99	0.84	0.03	0.50	0.99
RM17	0.50	0.50	1.00	0.86	0.03	0.50	1.00	0.51	0.50	0.99	0.83	0.03	0.51	0.99
RM111	0.53	0.50	1.00	0.87	0.03	0.50	1.00	0.51	0.50	0.99	0.84	0.03	0.50	0.99
RM143	**0.50**	**0.50**	**1.00**	**0.86**	**0.03**	**0.50**	**1.00**	0.49	0.33	0.98	0.73	0.04	0.39	0.98
RM333	**0.50**	**0.50**	**1.00**	**0.86**	**0.03**	**0.50**	**1.00**	**0.50**	**0.50**	**0.99**	**0.83**	**0.03**	**0.50**	**0.99**
RM4128	0.52	0.50	1.00	0.86	0.03	0.50	1.00	**0.50**	**0.50**	**0.99**	**0.84**	**0.03**	**0.51**	**0.99**
RM168	0.55	0.50	1.00	0.86	0.03	0.51	1.00	0.56	0.50	0.99	0.84	0.03	0.50	0.99
RM565	0.53	0.50	1.00	0.85	0.03	0.51	1.00	0.53	0.50	0.99	0.84	0.03	0.50	0.99
RM16074	**0.50**	**0.50**	**1.00**	**0.87**	**0.03**	**0.50**	**1.00**	**0.50**	**0.50**	**0.99**	**0.84**	**0.03**	**0.51**	**0.99**
RM20468	0.51	0.50	1.00	0.86	0.03	0.50	1.00	0.51	0.50	0.99	0.83	0.03	0.50	0.99
RM130	0.51	0.50	1.00	0.87	0.03	0.50	1.00	**0.50**	**0.50**	**0.99**	**0.84**	**0.03**	**0.50**	**0.99**
RM20460	0.51	0.50	1.00	0.85	0.03	0.50	1.00	0.50	0.50	0.99	0.84	0.03	0.50	0.99
RM5371	0.52	0.50	1.00	0.86	0.03	0.50	1.00	0.51	0.50	0.99	0.84	0.03	0.50	0.99
RM16071	0.51	0.50	1.00	0.87	0.03	0.50	1.00	**0.50**	**0.50**	**0.99**	**0.83**	**0.03**	**0.50**	**0.99**
	F_2 WM-RM277							F_2 non-WM-RM277						
	Obs.F	Min F	Max F	Mean*	SE*	L95*	U95*	Obs. F	Min F	Max F	Mean*	SE*	L95*	U95*
RM84	0.54	0.50	1.00	0.87	0.03	0.50	1.00	0.55	0.50	0.99	0.84	0.03	0.50	0.99
RM575	**0.50**	**0.50**	**1.00**	**0.86**	**0.03**	**0.50**	**1.00**	**0.50**	**0.50**	**0.99**	**0.84**	**0.03**	**0.50**	**0.99**

RM572	**0.50**	**0.50**	**1.00**	**0.86**	**0.03**	**0.50**	**1.00**	0.50	0.50	0.99	0.84	0.03	0.50	0.99
RM9	0.51	0.50	1.00	0.86	0.03	0.50	1.00	**0.50**	**0.50**	**0.99**	**0.84**	**0.03**	**0.50**	**0.99**
RM5	**0.51**	**0.50**	**1.00**	**0.87**	**0.03**	**0.51**	**1.00**	0.50	0.50	0.99	0.83	0.03	0.50	0.99
RM212	0.52	0.50	1.00	0.87	0.03	0.51	1.00	0.51	0.50	0.99	0.84	0.03	0.50	0.99
RM6895	0.55	0.50	1.00	0.86	0.03	0.51	1.00	0.53	0.50	0.99	0.85	0.03	0.50	0.99
RM7245	0.51	0.50	1.00	0.87	0.02	0.51	1.00	0.51	0.50	0.99	0.83	0.03	0.50	0.99
RM523	0.53	0.50	1.00	0.87	0.03	0.50	1.00	0.53	0.50	0.99	0.85	0.03	0.50	0.99
RM282	0.59	0.50	1.00	0.85	0.03	0.50	1.00	0.61	0.50	0.99	0.84	0.03	0.50	0.99
RM16	0.53	0.50	1.00	0.85	0.03	0.50	1.00	0.55	0.50	0.99	0.85	0.03	0.50	0.99
RM186	0.60	0.50	1.00	0.86	0.03	0.50	1.00	0.55	0.50	0.99	0.84	0.03	0.50	0.99
RM468	0.57	0.50	1.00	0.86	0.03	0.51	1.00	0.53	0.50	0.99	0.84	0.03	0.50	0.99
RM6395	0.55	0.50	1.00	0.86	0.03	0.51	1.00	0.51	0.50	0.99	0.83	0.03	0.50	0.99
RM6089	0.50	0.50	1.00	0.87	0.03	0.50	1.00	**0.50**	**0.50**	**0.99**	**0.84**	**0.03**	**0.50**	**0.99**
RM3558	0.51	0.50	1.00	0.86	0.03	0.50	1.00	0.53	0.50	0.99	0.84	0.03	0.50	0.99
RM6748	**0.50**	**0.50**	**1.00**	**0.87**	**0.03**	**0.50**	**1.00**	**0.50**	**0.50**	**0.99**	**0.84**	**0.03**	**0.50**	**0.99**
RM3419	**0.50**	**0.50**	**1.00**	**0.86**	**0.03**	**0.50**	**1.00**	0.51	0.50	0.99	0.84	0.03	0.50	0.99
RM6841	0.60	0.50	1.00	0.86	0.03	0.50	1.00	0.59	0.50	0.99	0.84	0.03	0.50	0.99
RM190	0.59	0.50	1.00	0.86	0.03	0.51	1.00	0.61	0.50	0.99	0.85	0.03	0.50	0.99
RM276	**0.50**	**0.50**	**1.00**	**0.86**	**0.03**	**0.50**	**1.00**	**0.50**	**0.50**	**0.99**	**0.84**	**0.03**	**0.50**	**0.99**
RM214	0.59	0.50	1.00	0.86	0.03	0.50	1.00	0.58	0.50	0.99	0.85	0.03	0.50	0.99
RM11	0.51	0.50	1.00	0.87	0.03	0.50	1.00	**0.50**	**0.50**	**0.99**	**0.85**	**0.03**	**0.50**	**0.99**
RM505	0.54	0.50	1.00	0.86	0.03	0.50	1.00	0.57	0.50	0.99	0.84	0.03	0.50	0.99
RM172	**0.50**	**0.50**	**1.00**	**0.86**	**0.03**	**0.50**	**1.00**	**0.50**	**0.50**	**0.99**	**0.85**	**0.03**	**0.51**	**0.99**
RM408	**0.50**	**0.50**	**1.00**	**0.86**	**0.03**	**0.50**	**1.00**	0.51	0.50	0.99	0.85	0.03	0.51	0.99
RM5068	0.71	0.50	1.00	0.85	0.03	0.51	1.00	0.56	0.50	0.99	0.84	0.03	0.50	0.99
RM331	0.52	0.50	1.00	0.87	0.03	0.50	1.00	0.55	0.50	0.99	0.84	0.03	0.50	0.99

RM296	0.51	0.50	1.00	0.86	0.03	0.50	1.00	0.53	0.50	0.99	0.85	0.03	0.50	0.99
RM4455	0.53	0.50	1.00	0.86	0.03	0.50	1.00	0.51	0.50	0.99	0.84	0.03	0.50	0.99
RM7557	0.51	0.50	1.00	0.86	0.03	0.50	1.00	**0.50**	**0.50**	**0.99**	**0.85**	**0.03**	**0.51**	**0.99**
RM332	0.52	0.50	1.00	0.85	0.03	0.51	1.00	0.52	0.50	0.99	0.85	0.03	0.50	0.99
RM4862	0.66	0.50	1.00	0.86	0.03	0.51	1.00	0.70	0.50	0.99	0.84	0.03	0.50	0.99
RM224	0.52	0.50	1.00	0.86	0.03	0.50	1.00	0.51	0.50	0.99	0.84	0.03	0.50	0.99
RM8216	0.54	0.50	1.00	0.85	0.03	0.50	1.00	0.58	0.50	0.99	0.85	0.03	0.51	0.99
RM3483	**0.50**	**0.50**	**1.00**	**0.87**	**0.03**	**0.50**	**1.00**	0.57	0.50	0.99	0.84	0.03	0.50	0.99
RM6296	0.52	0.50	1.00	0.86	0.03	0.50	1.00	0.75	0.50	0.99	0.83	0.03	0.50	0.99
RM277	-	-	-	-	-	-	-	-	-	-	-	-	-	-
RM6732	0.54	0.50	1.00	0.86	0.03	0.51	1.00	0.58	0.50	0.99	0.84	0.03	0.50	0.99
RM17	**0.50**	**0.50**	**1.00**	**0.86**	**0.03**	**0.50**	**1.00**	0.50	0.50	0.99	0.85	0.03	0.50	0.99
RM111	0.53	0.50	1.00	0.86	0.03	0.50	1.00	0.53	0.50	0.99	0.85	0.03	0.50	0.99
RM143	0.50	0.33	0.99	0.76	0.03	0.41	0.99	0.52	0.50	0.99	0.85	0.03	0.50	0.99
RM333	**0.50**	**0.50**	**1.00**	**0.86**	**0.03**	**0.51**	**1.00**	**0.50**	**0.50**	**0.99**	**0.84**	**0.03**	**0.50**	**0.99**
RM4128	0.51	0.50	1.00	0.86	0.03	0.51	1.00	0.52	0.50	0.99	0.85	0.03	0.50	0.99
RM168	0.56	0.50	1.00	0.86	0.03	0.51	1.00	0.54	0.50	0.99	0.84	0.03	0.50	0.99
RM565	0.55	0.50	1.00	0.86	0.03	0.51	1.00	0.50	0.50	0.99	0.84	0.03	0.50	0.99
RM16074	**0.50**	**0.50**	**1.00**	**0.86**	**0.03**	**0.50**	**1.00**	**0.50**	**0.50**	**0.99**	**0.86**	**0.03**	**0.51**	**0.99**
RM20468	0.52	0.50	1.00	0.87	0.03	0.50	1.00	0.50	0.50	0.99	0.85	0.03	0.50	0.99
RM130	**0.50**	**0.50**	**1.00**	**0.87**	**0.03**	**0.51**	**1.00**	0.51	0.50	0.99	0.85	0.03	0.50	0.99
RM20460	0.52	0.50	1.00	0.87	0.03	0.50	1.00	**0.50**	**0.50**	**0.99**	**0.84**	**0.03**	**0.50**	**0.99**
RM5371	0.53	0.50	1.00	0.85	0.03	0.50	1.00	0.51	0.50	0.99	0.85	0.03	0.50	0.99
RM16071	**0.50**	**0.50**	**1.00**	**0.86**	**0.03**	**0.50**	**1.00**	0.51	0.50	0.99	0.84	0.03	0.50	0.99

F_2 Ideal-1 F_2 Ideal-2

	Obs. F	Min F	Max F	Mean*	SE*	L95*	U95*	Obs. F	Min F	Max F	Mean*	SE*	L95*	U95*
RM84	0.53	0.50	1.00	0.89	0.03	0.51	1.00	0.52	0.50	1.00	0.89	0.02	0.50	1.00
RM575	0.52	0.50	1.00	0.88	0.03	0.51	1.00	0.52	0.50	1.00	0.89	0.02	0.51	1.00
RM572	**0.50**	**0.50**	**1.00**	**0.88**	**0.03**	**0.51**	**1.00**	**0.50**	**0.50**	**1.00**	**0.88**	**0.03**	**0.51**	**1.00**
RM9	**0.50**	**0.50**	**1.00**	**0.88**	**0.03**	**0.51**	**1.00**	**0.50**	**0.50**	**1.00**	**0.88**	**0.03**	**0.50**	**1.00**
RM5	**0.50**	**0.50**	**1.00**	**0.90**	**0.02**	**0.51**	**1.00**	**0.50**	**0.50**	**1.00**	**0.89**	**0.03**	**0.51**	**1.00**
RM212	0.53	0.50	1.00	0.89	0.02	0.51	1.00	0.52	0.50	1.00	0.88	0.03	0.50	1.00
RM6895	**0.50**	**0.50**	**1.00**	**0.89**	**0.03**	**0.51**	**1.00**	**0.50**	**0.50**	**1.00**	**0.88**	**0.03**	**0.50**	**1.00**
RM7245	**0.50**	**0.50**	**1.00**	**0.89**	**0.03**	**0.50**	**1.00**	**0.50**	**0.50**	**1.00**	**0.88**	**0.03**	**0.51**	**1.00**
RM523	**0.50**	**0.50**	**1.00**	**0.88**	**0.03**	**0.51**	**1.00**	**0.50**	**0.50**	**1.00**	**0.87**	**0.03**	**0.51**	**1.00**
RM282	0.53	0.50	1.00	0.88	0.03	0.51	1.00	0.53	0.50	1.00	0.86	0.03	0.50	1.00
RM16	0.51	0.50	1.00	0.89	0.02	0.51	1.00	0.52	0.50	1.00	0.88	0.03	0.50	1.00
RM186	0.58	0.50	1.00	0.88	0.03	0.51	1.00	0.57	0.50	1.00	0.87	0.03	0.50	1.00
RM468	0.57	0.50	1.00	0.89	0.03	0.50	1.00	0.56	0.50	1.00	0.88	0.03	0.51	1.00
RM565	**0.50**	**0.50**	**1.00**	**0.89**	**0.02**	**0.50**	**1.00**	**0.50**	**0.50**	**1.00**	**0.89**	**0.02**	**0.51**	**1.00**
RM6089	**0.50**	**0.50**	**1.00**	**0.88**	**0.03**	**0.50**	**1.00**	**0.50**	**0.50**	**1.00**	**0.88**	**0.03**	**0.50**	**1.00**
RM3558	**0.50**	**0.50**	**1.00**	**0.88**	**0.03**	**0.50**	**1.00**	**0.50**	**0.50**	**1.00**	**0.88**	**0.03**	**0.50**	**1.00**
RM6748	**0.50**	**0.50**	**1.00**	**0.89**	**0.02**	**0.51**	**1.00**	**0.50**	**0.50**	**1.00**	**0.89**	**0.02**	**0.51**	**1.00**
RM3419	0.51	0.50	1.00	0.88	0.03	0.51	1.00	**0.50**	**0.50**	**1.00**	**0.89**	**0.02**	**0.51**	**1.00**
RM6841	0.59	0.50	1.00	0.89	0.02	0.51	1.00	0.59	0.50	1.00	0.88	0.03	0.51	1.00
RM190	0.67	0.50	1.00	0.89	0.02	0.50	1.00	0.66	0.50	1.00	0.88	0.02	0.51	1.00
RM276	**0.50**	**0.50**	**1.00**	**0.89**	**0.03**	**0.50**	**1.00**	**0.50**	**0.50**	**1.00**	**0.88**	**0.03**	**0.51**	**1.00**
RM214	0.56	0.50	1.00	0.89	0.02	0.51	1.00	0.57	0.50	1.00	0.88	0.03	0.51	1.00
RM11	**0.50**	**0.50**	**1.00**	**0.89**	**0.02**	**0.51**	**1.00**	**0.51**	**0.50**	**1.00**	**0.87**	**0.03**	**0.51**	**1.00**
RM505	0.51	0.50	1.00	0.90	0.02	0.51	1.00	0.51	0.50	1.00	0.88	0.02	0.51	1.00
RM172	**0.50**	**0.50**	**1.00**	**0.89**	**0.02**	**0.51**	**1.00**	**0.50**	**0.50**	**1.00**	**0.88**	**0.03**	**0.51**	**1.00**

RM408	0.52	0.50	1.00	0.90	0.02	0.51	1.00	0.51	0.50	1.00	0.88	0.03	0.51	1.00
RM5068	0.72	0.50	1.00	0.89	0.02	0.51	1.00	0.73	0.50	1.00	0.88	0.02	0.51	1.00
RM331	0.52	0.50	1.00	0.88	0.03	0.51	1.00	0.52	0.50	1.00	0.88	0.03	0.50	1.00
RM296	0.51	0.50	1.00	0.88	0.03	0.51	1.00	**0.51**	**0.50**	**1.00**	**0.88**	**0.03**	**0.51**	**1.00**
RM4455	0.52	0.50	1.00	0.89	0.03	0.51	1.00	0.53	0.50	1.00	0.88	0.03	0.50	1.00
RM7557	0.51	0.50	1.00	0.89	0.02	0.51	1.00	0.51	0.50	1.00	0.88	0.03	0.50	1.00
RM332	**0.50**	**0.50**	**1.00**	**0.89**	**0.03**	**0.51**	**1.00**	**0.50**	**0.50**	**1.00**	**0.88**	**0.03**	**0.51**	**1.00**
RM4862	0.71	0.50	1.00	0.89	0.02	0.50	1.00	0.70	0.50	1.00	0.88	0.03	0.51	1.00
RM224	0.53	0.50	1.00	0.89	0.02	0.51	1.00	0.54	0.50	1.00	0.89	0.02	0.51	1.00
RM8216	**0.50**	**0.50**	**1.00**	**0.89**	**0.02**	**0.51**	**1.00**	**0.50**	**0.50**	**1.00**	**0.87**	**0.03**	**0.51**	**1.00**
RM3483	0.51	0.50	1.00	0.89	0.03	0.51	1.00	**0.50**	**0.50**	**1.00**	**0.89**	**0.02**	**0.51**	**1.00**
RM6296	**0.50**	**0.50**	**1.00**	**0.88**	**0.03**	**0.51**	**1.00**	**0.50**	**0.50**	**1.00**	**0.89**	**0.02**	**0.51**	**1.00**
RM277	**0.50**	**0.50**	**1.00**	**0.89**	**0.03**	**0.51**	**1.00**	**0.50**	**0.50**	**1.00**	**0.88**	**0.03**	**0.51**	**1.00**
RM6732	0.58	0.50	1.00	0.89	0.02	0.51	1.00	0.59	0.50	1.00	0.89	0.02	0.51	1.00
RM17	0.56	0.50	1.00	0.89	0.02	0.51	1.00	0.56	0.50	1.00	0.88	0.03	0.51	1.00
RM111	0.52	0.50	1.00	0.87	0.03	0.50	1.00	0.53	0.50	1.00	0.85	0.03	0.50	1.00
RM143	0.51	0.50	1.00	0.90	0.02	0.51	1.00	0.51	0.50	1.00	0.88	0.03	0.51	1.00
RM333	**0.50**	**0.50**	**1.00**	**0.88**	**0.03**	**0.51**	**1.00**	**0.50**	**0.50**	**1.00**	**0.89**	**0.02**	**0.50**	**1.00**
RM4128	**0.50**	**0.50**	**1.00**	**0.89**	**0.03**	**0.51**	**1.00**	**0.50**	**0.50**	**1.00**	**0.88**	**0.03**	**0.50**	**1.00**
RM168	0.57	0.50	1.00	0.88	0.03	0.51	1.00	0.56	0.50	1.00	0.88	0.03	0.51	1.00
RM6395	0.55	0.50	1.00	0.89	0.03	0.51	1.00	0.53	0.50	1.00	0.88	0.03	0.50	1.00
RM16074	**0.50**	**0.50**	**1.00**	**0.89**	**0.03**	**0.51**	**1.00**	**0.50**	**0.50**	**1.00**	**0.88**	**0.03**	**0.50**	**1.00**
RM20468	**0.50**	**0.50**	**1.00**	**0.89**	**0.02**	**0.51**	**1.00**	**0.50**	**0.50**	**1.00**	**0.88**	**0.03**	**0.51**	**1.00**
RM130	**0.50**	**0.50**	**1.00**	**0.89**	**0.03**	**0.51**	**1.00**	**0.50**	**0.50**	**1.00**	**0.88**	**0.02**	**0.51**	**1.00**
RM20460	**0.50**	**0.50**	**1.00**	**0.88**	**0.03**	**0.50**	**1.00**	**0.50**	**0.50**	**1.00**	**0.88**	**0.02**	**0.51**	**1.00**
RM5371	**0.51**	**0.50**	**1.00**	**0.88**	**0.03**	**0.51**	**1.00**	**0.50**	**0.50**	**1.00**	**0.87**	**0.03**	**0.51**	**1.00**

RM16071	**0.51**	**0.50**	**1.00**	**0.89**	**0.02**	**0.51**	**1.00**	0.51	0.50	1.00	0.89	0.02	0.51	1.00

	F_3 GE							F_3 non-GE						
	Obs.F	Min F	Max F	Mean*	SE*	L95*	U95*	Obs. F	Min F	Max F	Mean*	SE*	L95*	U95*
RM84	0.52	0.50	1.00	0.86	0.03	0.50	1.00	0.53	0.50	0.99	0.84	0.03	0.50	0.99
RM575	**0.50**	**0.50**	**1.00**	**0.85**	**0.03**	**0.50**	**1.00**	**0.50**	**0.50**	**0.99**	**0.85**	**0.03**	**0.51**	**0.99**
RM572	**0.50**	**0.50**	**1.00**	**0.86**	**0.03**	**0.50**	**1.00**	**0.50**	**0.50**	**0.99**	**0.85**	**0.03**	**0.50**	**0.99**
RM9	**0.50**	**0.50**	**1.00**	**0.87**	**0.03**	**0.50**	**1.00**	**0.50**	**0.50**	**0.99**	**0.84**	**0.03**	**0.50**	**0.99**
RM5	0.52	0.50	1.00	0.86	0.03	0.50	1.00	0.51	0.50	0.99	0.84	0.03	0.50	0.99
RM212	**0.50**	**0.50**	**1.00**	**0.87**	**0.03**	**0.50**	**1.00**	0.51	0.50	0.99	0.84	0.03	0.50	0.99
RM6895	0.50	0.50	1.00	0.85	0.03	0.50	1.00	0.57	0.50	0.99	0.84	0.03	0.50	0.99
RM7245	0.52	0.50	1.00	0.86	0.03	0.50	1.00	0.58	0.50	0.99	0.85	0.03	0.50	0.99
RM523	0.52	0.50	1.00	0.86	0.03	0.50	1.00	0.54	0.50	0.99	0.85	0.03	0.50	0.99
RM282	0.60	0.50	1.00	0.86	0.03	0.51	1.00	0.62	0.50	0.99	0.86	0.03	0.50	0.99
RM16	0.53	0.50	1.00	0.86	0.03	0.51	1.00	**0.50**	**0.50**	**0.99**	**0.85**	**0.03**	**0.50**	**0.99**
RM186	0.51	0.50	1.00	0.86	0.03	0.50	1.00	**0.50**	**0.50**	**0.99**	**0.85**	**0.03**	**0.50**	**0.99**
RM468	0.50	0.50	1.00	0.85	0.03	0.50	1.00	**0.50**	**0.50**	**0.99**	**0.85**	**0.03**	**0.50**	**0.99**
RM6395	0.55	0.50	1.00	0.86	0.02	0.51	1.00	0.54	0.50	0.99	0.86	0.03	0.50	0.99
RM6089	0.51	0.50	1.00	0.85	0.03	0.50	1.00	0.51	0.50	0.99	0.84	0.03	0.50	0.99
RM3558	**0.50**	**0.50**	**1.00**	**0.85**	**0.03**	**0.50**	**1.00**	**0.50**	**0.50**	**0.99**	**0.84**	**0.03**	**0.50**	**0.99**
RM6748	0.55	0.50	1.00	0.86	0.03	0.50	1.00	0.53	0.50	0.99	0.84	0.03	0.50	0.99
RM3419	0.50	0.50	1.00	0.86	0.03	0.50	1.00	0.56	0.50	0.99	0.84	0.03	0.50	0.99
RM6841	0.57	0.50	1.00	0.85	0.03	0.50	1.00	0.72	0.50	0.99	0.86	0.03	0.51	0.99
RM190	0.52	0.50	1.00	0.85	0.03	0.50	1.00	**0.50**	**0.50**	**0.99**	**0.84**	**0.03**	**0.51**	**0.99**
RM276	0.60	0.50	1.00	0.86	0.03	0.51	1.00	**0.50**	**0.50**	**0.99**	**0.85**	**0.03**	**0.50**	**0.99**
RM214	**0.50**	**0.50**	**1.00**	**0.86**	**0.03**	**0.51**	**1.00**	0.51	0.50	0.99	0.84	0.03	0.51	0.99

RM11	0.55	0.50	1.00	0.86	0.03	0.50	1.00	0.55	0.50	0.99	0.84	0.03	0.50	0.99
RM505	0.56	0.50	1.00	0.86	0.03	0.51	1.00	0.69	0.50	0.99	0.85	0.03	0.50	0.99
RM172	0.54	0.50	1.00	0.86	0.03	0.50	1.00	0.56	0.50	0.99	0.84	0.03	0.50	0.99
RM408	**0.50**	**0.50**	**1.00**	**0.86**	**0.03**	**0.51**	**1.00**	0.51	0.50	0.99	0.84	0.03	0.50	0.99
RM5068	0.62	0.50	1.00	0.87	0.03	0.51	1.00	0.53	0.50	0.99	0.85	0.03	0.50	0.99
RM331	**0.50**	**0.50**	**1.00**	**0.86**	**0.03**	**0.50**	**1.00**	0.56	0.50	0.99	0.84	0.03	0.50	0.99
RM296	**0.51**	**0.50**	**1.00**	**0.87**	**0.03**	**0.51**	**1.00**	0.56	0.50	0.99	0.85	0.03	0.50	0.99
RM4455	0.51	0.50	1.00	0.86	0.03	0.51	1.00	0.51	0.50	0.99	0.85	0.03	0.50	0.99
RM7557	**0.50**	**0.50**	**1.00**	**0.85**	**0.03**	**0.50**	**1.00**	0.52	0.50	0.99	0.83	0.03	0.50	0.99
RM332	**0.50**	**0.50**	**1.00**	**0.86**	**0.03**	**0.51**	**1.00**	0.53	0.50	0.99	0.85	0.03	0.50	0.99
RM4862	0.65	0.50	1.00	0.86	0.03	0.51	1.00	0.71	0.50	0.99	0.84	0.03	0.50	0.99
RM224	0.51	0.50	1.00	0.86	0.03	0.51	1.00	0.54	0.50	0.99	0.85	0.03	0.51	0.99
RM8216	0.58	0.50	1.00	0.85	0.03	0.50	1.00	0.58	0.50	0.99	0.86	0.03	0.51	0.99
RM3483	0.61	0.50	1.00	0.86	0.03	0.51	1.00	0.60	0.50	0.99	0.84	0.03	0.50	0.99
RM6296	0.53	0.50	1.00	0.87	0.02	0.50	1.00	0.52	0.50	0.99	0.85	0.03	0.50	0.99
RM277	**0.50**	**0.50**	**1.00**	**0.86**	**0.03**	**0.50**	**1.00**	0.51	0.50	0.99	0.86	0.03	0.50	0.99
RM6732	**0.50**	**0.50**	**1.00**	**0.86**	**0.03**	**0.50**	**1.00**	**0.50**	**0.50**	**0.99**	**0.84**	**0.03**	**0.50**	**0.99**
RM17	0.54	0.50	1.00	0.86	0.03	0.50	1.00	0.53	0.50	0.99	0.83	0.03	0.50	0.99
RM111	0.57	0.50	1.00	0.87	0.02	0.51	1.00	**0.50**	**0.50**	**0.99**	**0.85**	**0.03**	**0.51**	**0.99**
RM143	**0.50**	**0.50**	**1.00**	**0.86**	**0.03**	**0.50**	**1.00**	**0.50**	**0.50**	**0.99**	**0.85**	**0.03**	**0.51**	**0.99**
RM333	0.58	0.50	1.00	0.85	0.03	0.50	1.00	0.53	0.50	0.99	0.83	0.03	0.50	0.99
RM4128	0.71	0.50	1.00	0.86	0.03	0.51	1.00	0.54	0.50	0.99	0.85	0.03	0.50	0.99
RM168	0.55	0.50	1.00	0.85	0.03	0.50	1.00	**0.50**	**0.50**	**0.99**	**0.84**	**0.03**	**0.50**	**0.99**
RM565	0.55	0.50	1.00	0.87	0.03	0.51	1.00	0.52	0.50	0.99	0.84	0.03	0.50	0.99
RM16074	**0.50**	**0.50**	**1.00**	**0.86**	**0.03**	**0.50**	**1.00**	**0.50**	**0.50**	**0.99**	**0.84**	**0.03**	**0.50**	**0.99**
RM20468	**0.50**	**0.50**	**1.00**	**0.85**	**0.03**	**0.50**	**1.00**	**0.50**	**0.50**	**0.99**	**0.84**	**0.03**	**0.50**	**0.99**

RM130	**0.50**	**0.50**	**1.00**	**0.85**	**0.03**	**0.51**	**1.00**	0.51	0.50	0.99	0.85	0.03	0.51	0.99
RM20460	**0.50**	**0.50**	**1.00**	**0.86**	**0.03**	**0.50**	**1.00**	**0.50**	**0.50**	**0.99**	**0.84**	**0.03**	**0.50**	**0.99**
RM5371	0.52	0.50	1.00	0.86	0.03	0.50	1.00	0.52	0.50	0.99	0.85	0.03	0.51	0.99
RM16071	0.50	0.50	1.00	0.86	0.03	0.50	1.00	0.51	0.50	0.99	0.85	0.03	0.50	0.99

	F_3 CM-RM572							F_3 non-CM-RM572						
	Obs.F	Min F	Max F	Mean*	SE*	L95*	U95*	Obs. F	Min F	Max F	Mean*	SE*	L95*	U95*
RM84	0.56	0.50	1.00	0.85	0.03	0.51	1.00	**0.50**	**0.50**	**0.99**	**0.85**	**0.03**	**0.50**	**0.99**
RM575	**0.50**	**0.50**	**1.00**	**0.86**	**0.03**	**0.51**	**1.00**	0.70	0.50	0.99	0.83	0.03	0.50	0.99
RM572	-	-	-	-	-	-	-	-	-	-	-	-	-	-
RM9	**0.50**	**0.50**	**1.00**	**0.85**	**0.03**	**0.50**	**1.00**	0.51	0.50	0.99	0.82	0.03	0.50	0.99
RM5	**0.50**	**0.50**	**1.00**	**0.85**	**0.03**	**0.50**	**1.00**	0.51	0.50	0.99	0.84	0.03	0.51	0.99
RM212	0.52	0.50	1.00	0.85	0.03	0.51	1.00	0.55	0.50	0.99	0.84	0.03	0.51	0.99
RM6895	**0.50**	**0.50**	**1.00**	**0.85**	**0.03**	**0.50**	**1.00**	0.51	0.50	0.99	0.83	0.03	0.50	0.99
RM7245	**0.50**	**0.50**	**1.00**	**0.85**	**0.03**	**0.50**	**1.00**	0.51	0.50	0.99	0.84	0.03	0.50	0.99
RM523	0.50	0.50	0.99	0.85	0.03	0.50	0.99	**0.50**	**0.50**	**0.99**	**0.83**	**0.03**	**0.50**	**0.99**
RM282	0.53	0.50	0.99	0.84	0.03	0.50	0.99	0.54	0.50	0.99	0.82	0.03	0.50	0.99
RM16	0.52	0.50	1.00	0.86	0.03	0.50	1.00	0.51	0.50	0.99	0.84	0.03	0.50	0.99
RM186	0.56	0.50	0.99	0.85	0.03	0.50	0.99	0.61	0.50	0.99	0.82	0.03	0.50	0.99
RM468	0.55	0.50	1.00	0.85	0.03	0.51	1.00	0.58	0.50	0.99	0.84	0.03	0.51	0.99
RM6395	**0.50**	**0.50**	**1.00**	**0.85**	**0.03**	**0.51**	**1.00**	**0.50**	**0.50**	**0.99**	**0.83**	**0.03**	**0.50**	**0.99**
RM6089	**0.50**	**0.50**	**1.00**	**0.85**	**0.03**	**0.50**	**1.00**	**0.50**	**0.50**	**0.99**	**0.84**	**0.03**	**0.51**	**0.99**
RM3558	**0.50**	**0.50**	**1.00**	**0.85**	**0.03**	**0.51**	**1.00**	0.51	0.50	0.99	0.84	0.03	0.50	0.99
RM6748	**0.50**	**0.50**	**1.00**	**0.86**	**0.03**	**0.51**	**1.00**	**0.50**	**0.50**	**0.99**	**0.83**	**0.03**	**0.50**	**0.99**
RM3419	0.51	0.50	0.99	0.84	0.03	0.50	0.99	**0.50**	**0.50**	**0.99**	**0.83**	**0.03**	**0.50**	**0.99**
RM6841	0.60	0.50	1.00	0.85	0.03	0.50	1.00	0.56	0.50	0.99	0.85	0.03	0.50	0.99

RM190	0.65	0.50	1.00	0.85	0.03	0.50	1.00	0.71	0.50	0.99	0.84	0.03	0.50	0.99
RM276	0.50	0.50	1.00	0.85	0.03	0.50	1.00	0.51	0.50	0.99	0.84	0.03	0.50	0.99
RM214	0.55	0.50	1.00	0.85	0.03	0.50	1.00	0.57	0.50	0.99	0.84	0.03	0.50	0.99
RM11	0.50	0.50	1.00	0.84	0.03	0.50	1.00	0.51	0.50	0.99	0.82	0.03	0.50	0.99
RM505	0.52	0.50	1.00	0.84	0.03	0.50	1.00	0.51	0.50	0.99	0.83	0.03	0.50	0.99
RM172	**0.50**	**0.50**	**1.00**	**0.85**	**0.03**	**0.50**	**1.00**	0.50	0.50	0.99	0.84	0.03	0.50	0.99
RM408	0.53	0.50	1.00	0.85	0.03	0.50	1.00	0.51	0.50	0.99	0.83	0.03	0.50	0.99
RM5068	0.72	0.50	1.00	0.85	0.03	0.50	1.00	0.72	0.50	0.99	0.82	0.03	0.50	0.99
RM331	0.52	0.50	1.00	0.84	0.03	0.50	1.00	0.52	0.50	0.99	0.85	0.03	0.50	0.99
RM296	0.51	0.50	1.00	0.85	0.03	0.50	1.00	0.51	0.50	0.99	0.83	0.03	0.50	0.99
RM4455	0.51	0.50	1.00	0.86	0.03	0.50	1.00	0.55	0.50	0.99	0.84	0.03	0.50	0.99
RM7557	0.51	0.50	1.00	0.84	0.03	0.50	1.00	0.52	0.50	0.99	0.82	0.03	0.50	0.99
RM332	**0.50**	**0.50**	**1.00**	**0.86**	**0.03**	**0.50**	**1.00**	**0.50**	**0.50**	**0.99**	**0.83**	**0.03**	**0.50**	**0.99**
RM4862	0.71	0.50	1.00	0.85	0.03	0.50	1.00	0.71	0.50	0.99	0.85	0.03	0.50	0.99
RM224	0.54	0.50	1.00	0.85	0.03	0.50	1.00	0.53	0.50	0.99	0.84	0.03	0.50	0.99
RM8216	**0.50**	**0.50**	**1.00**	**0.84**	**0.03**	**0.50**	**1.00**	0.52	0.50	0.99	0.84	0.03	0.50	0.99
RM3483	0.51	0.50	1.00	0.85	0.03	0.50	1.00	0.50	0.50	0.99	0.83	0.03	0.50	0.99
RM6296	**0.50**	**0.50**	**1.00**	**0.85**	**0.03**	**0.50**	**1.00**	0.51	0.50	0.99	0.83	0.03	0.50	0.99
RM277	**0.50**	**0.50**	**1.00**	**0.85**	**0.03**	**0.50**	**1.00**	**0.50**	**0.50**	**0.99**	**0.83**	**0.03**	**0.50**	**0.99**
RM6732	0.55	0.50	1.00	0.85	0.03	0.50	1.00	0.67	0.50	0.99	0.83	0.03	0.50	0.99
RM17	0.55	0.50	1.00	0.85	0.03	0.50	1.00	0.58	0.50	0.99	0.84	0.03	0.50	0.99
RM111	0.52	0.50	0.98	0.81	0.03	0.50	0.98	0.53	0.50	0.96	0.77	0.03	0.50	0.96
RM143	0.52	0.50	1.00	0.85	0.03	0.50	1.00	0.50	0.50	0.99	0.83	0.03	0.50	0.99
RM333	**0.50**	**0.50**	**1.00**	**0.86**	**0.03**	**0.51**	**1.00**	**0.50**	**0.50**	**0.99**	**0.83**	**0.03**	**0.50**	**0.99**
RM4128	0.51	0.50	1.00	0.85	0.03	0.51	1.00	**0.50**	**0.50**	**0.99**	**0.83**	**0.03**	**0.50**	**0.99**
RM168	0.54	0.50	1.00	0.86	0.03	0.51	1.00	0.62	0.50	0.99	0.83	0.03	0.50	0.99

RM565	0.54	0.50	1.00	0.85	0.03	0.50	1.00	0.56	0.50	0.99	0.83	0.03	0.50	0.99
RM16074	**0.50**	**0.50**	**1.00**	**0.85**	**0.03**	**0.50**	**1.00**	**0.50**	**0.50**	**0.99**	**0.83**	**0.03**	**0.50**	**0.99**
RM20468	**0.50**	**0.50**	**1.00**	**0.86**	**0.03**	**0.50**	**1.00**	**0.50**	**0.50**	**0.99**	**0.84**	**0.03**	**0.51**	**0.99**
RM130	**0.50**	**0.50**	**1.00**	**0.85**	**0.03**	**0.50**	**1.00**	**0.50**	**0.50**	**0.99**	**0.84**	**0.03**	**0.50**	**0.99**
RM20460	**0.50**	**0.50**	**1.00**	**0.86**	**0.03**	**0.51**	**1.00**	**0.50**	**0.50**	**0.99**	**0.84**	**0.03**	**0.50**	**0.99**
RM5371	0.51	0.50	1.00	0.86	0.03	0.50	1.00	**0.50**	**0.50**	**0.99**	**0.84**	**0.03**	**0.51**	**0.99**
RM16071	0.51	0.50	1.00	0.85	0.03	0.50	1.00	**0.50**	**0.50**	**0.99**	**0.83**	**0.03**	**0.50**	**0.99**

	F_3 WM-RM572							F_3 non-WM-RM572						
	Obs.F	Min F	Max F	Mean*	SE*	L95*	U95*	Obs. F	Min F	Max F	Mean*	SE*	L95*	U95*
RM84	0.51	0.50	1.00	0.86	0.03	0.51	1.00	0.59	0.50	0.99	0.83	0.03	0.50	0.99
RM575	0.56	0.50	1.00	0.85	0.03	0.51	1.00	0.54	0.50	0.99	0.84	0.03	0.50	0.99
RM572	-	-	-	-	-	-	-	-	-	-	-	-	-	-
RM9	0.51	0.50	1.00	0.85	0.03	0.50	1.00	0.53	0.50	0.99	0.83	0.03	0.50	0.99
RM5	0.51	0.50	1.00	0.86	0.03	0.50	1.00	0.52	0.50	0.99	0.83	0.03	0.50	0.99
RM212	0.54	0.50	1.00	0.86	0.03	0.51	1.00	0.51	0.50	0.99	0.84	0.03	0.50	0.99
RM6895	**0.50**	**0.50**	**1.00**	**0.86**	**0.03**	**0.50**	**1.00**	**0.50**	**0.50**	**0.99**	**0.83**	**0.03**	**0.50**	**0.99**
RM7245	**0.50**	**0.50**	**1.00**	**0.86**	**0.03**	**0.50**	**1.00**	**0.50**	**0.50**	**0.99**	**0.84**	**0.03**	**0.50**	**0.99**
RM523	**0.50**	**0.50**	**0.99**	**0.85**	**0.03**	**0.50**	**0.99**	0.51	0.50	0.99	0.83	0.03	0.50	0.99
RM282	0.54	0.50	0.99	0.84	0.03	0.50	0.99	0.53	0.50	0.99	0.81	0.03	0.50	0.99
RM16	0.51	0.50	1.00	0.86	0.03	0.50	1.00	0.54	0.50	0.99	0.83	0.03	0.51	0.99
RM186	0.60	0.50	0.99	0.84	0.03	0.50	0.99	0.53	0.50	0.99	0.82	0.03	0.50	0.99
RM468	0.57	0.50	1.00	0.85	0.03	0.50	1.00	0.54	0.50	0.99	0.83	0.03	0.50	0.99
RM6395	**0.50**	**0.50**	**1.00**	**0.85**	**0.03**	**0.50**	**1.00**	0.50	0.50	0.99	0.83	0.03	0.50	0.99
RM6089	**0.50**	**0.50**	**1.00**	**0.86**	**0.03**	**0.50**	**1.00**	**0.50**	**0.50**	**0.99**	**0.83**	**0.03**	**0.50**	**0.99**
RM3558	**0.50**	**0.50**	**1.00**	**0.85**	**0.03**	**0.50**	**1.00**	0.50	0.50	0.99	0.83	0.03	0.50	0.99

RM6748	**0.50**	**0.50**	**1.00**	**0.85**	**0.03**	**0.50**	**1.00**	**0.50**	**0.50**	**0.99**	**0.83**	**0.03**	**0.50**	**0.99**
RM3419	**0.50**	**0.50**	**1.00**	**0.86**	**0.03**	**0.51**	**1.00**	0.51	0.50	0.99	0.83	0.03	0.50	0.99
RM6841	0.58	0.50	1.00	0.86	0.03	0.51	1.00	0.60	0.50	0.99	0.83	0.03	0.50	0.99
RM190	0.69	0.50	1.00	0.86	0.03	0.50	1.00	0.61	0.50	0.99	0.83	0.03	0.50	0.99
RM276	**0.50**	**0.50**	**1.00**	**0.86**	**0.03**	**0.50**	**1.00**	0.51	0.50	0.99	0.83	0.03	0.50	0.99
RM214	0.56	0.50	1.00	0.85	0.03	0.50	1.00	0.55	0.50	0.99	0.84	0.03	0.50	0.99
RM11	0.51	0.50	1.00	0.86	0.03	0.50	1.00	**0.50**	**0.50**	**0.99**	**0.82**	**0.03**	**0.50**	**0.99**
RM505	0.52	0.50	1.00	0.85	0.03	0.50	1.00	0.51	0.50	0.99	0.84	0.03	0.50	0.99
RM172	**0.50**	**0.50**	**1.00**	**0.85**	**0.03**	**0.50**	**1.00**	**0.50**	**0.50**	**0.99**	**0.82**	**0.03**	**0.50**	**0.99**
RM408	0.52	0.50	1.00	0.85	0.03	0.50	1.00	0.52	0.50	0.99	0.83	0.03	0.50	0.99
RM5068	0.73	0.50	1.00	0.85	0.03	0.50	1.00	0.71	0.50	0.99	0.83	0.03	0.50	0.99
RM331	0.51	0.50	1.00	0.86	0.03	0.50	1.00	0.54	0.50	0.99	0.83	0.03	0.50	0.99
RM296	0.50	0.50	1.00	0.85	0.03	0.50	1.00	0.52	0.50	0.99	0.84	0.03	0.50	0.99
RM4455	0.53	0.50	1.00	0.85	0.03	0.50	1.00	0.51	0.50	0.99	0.83	0.03	0.50	0.99
RM7557	0.52	0.50	1.00	0.84	0.03	0.50	1.00	**0.50**	**0.50**	**0.99**	**0.83**	**0.03**	**0.50**	**0.99**
RM332	**0.50**	**0.50**	**1.00**	**0.86**	**0.03**	**0.51**	**1.00**	**0.50**	**0.50**	**0.99**	**0.83**	**0.03**	**0.50**	**0.99**
RM4862	0.72	0.50	1.00	0.85	0.03	0.50	1.00	0.67	0.50	0.99	0.83	0.03	0.50	0.99
RM224	0.54	0.50	1.00	0.85	0.03	0.50	1.00	0.53	0.50	0.99	0.83	0.03	0.50	0.99
RM8216	0.50	0.50	1.00	0.85	0.03	0.50	1.00	0.50	0.50	0.99	0.83	0.03	0.50	0.99
RM3483	**0.50**	**0.50**	**1.00**	**0.86**	**0.03**	**0.50**	**1.00**	0.55	0.50	0.99	0.83	0.03	0.50	0.99
RM6296	0.51	0.50	1.00	0.85	0.03	0.50	1.00	**0.50**	**0.50**	**0.99**	**0.82**	**0.03**	**0.50**	**0.99**
RM277	**0.50**	**0.50**	**1.00**	**0.86**	**0.03**	**0.50**	**1.00**	0.50	0.50	0.99	0.83	0.03	0.50	0.99
RM6732	0.58	0.50	1.00	0.85	0.03	0.50	1.00	0.58	0.50	0.99	0.83	0.03	0.50	0.99
RM17	0.56	0.50	1.00	0.85	0.03	0.51	1.00	0.56	0.50	0.99	0.83	0.03	0.50	0.99
RM111	0.53	0.50	0.98	0.79	0.03	0.50	0.98	0.51	0.50	0.95	0.76	0.03	0.50	0.95
RM143	0.51	0.50	1.00	0.85	0.03	0.50	1.00	0.52	0.50	0.99	0.82	0.03	0.50	0.99

RM333	**0.50**	**0.50**	**1.00**	**0.86**	**0.03**	**0.50**	**1.00**	**0.50**	**0.50**	**0.99**	**0.84**	**0.03**	**0.50**	**0.99**
RM4128	**0.50**	**0.50**	**1.00**	**0.86**	**0.03**	**0.50**	**1.00**	0.51	0.50	0.99	0.82	0.03	0.50	0.99
RM168	0.58	0.50	1.00	0.85	0.03	0.50	1.00	0.53	0.50	0.99	0.83	0.03	0.50	0.99
RM565	0.55	0.50	1.00	0.86	0.03	0.50	1.00	0.53	0.50	0.99	0.84	0.03	0.50	0.99
RM16074	**0.50**	**0.50**	**1.00**	**0.85**	**0.03**	**0.51**	**1.00**	**0.50**	**0.50**	**0.99**	**0.83**	**0.03**	**0.50**	**0.99**
RM20468	**0.50**	**0.50**	**1.00**	**0.85**	**0.03**	**0.50**	**1.00**	**0.50**	**0.50**	**0.99**	**0.83**	**0.03**	**0.50**	**0.99**
RM130	**0.50**	**0.50**	**1.00**	**0.86**	**0.03**	**0.50**	**1.00**	0.50	0.50	0.99	0.83	0.03	0.50	0.99
RM20460	**0.50**	**0.50**	**1.00**	**0.85**	**0.03**	**0.50**	**1.00**	**0.50**	**0.50**	**0.99**	**0.83**	**0.03**	**0.50**	**0.99**
RM5371	**0.50**	**0.50**	**1.00**	**0.86**	**0.03**	**0.50**	**1.00**	0.51	0.50	0.99	0.83	0.03	0.50	0.99
RM16071	**0.50**	**0.50**	**1.00**	**0.86**	**0.03**	**0.51**	**1.00**	0.53	0.50	0.99	0.82	0.03	0.50	0.99

	F_3 CM-RM408							F_3 non-CM-RM408						
	Obs.F	Min F	Max F	Mean*	SE*	L95*	U95*	Obs. F	Min F	Max F	Mean*	SE*	L95*	U95*
RM84	0.53	0.50	1.00	0.86	0.03	0.50	1.00	0.51	0.50	0.99	0.83	0.03	0.50	0.99
RM575	0.51	0.50	1.00	0.85	0.03	0.51	1.00	0.56	0.50	0.99	0.82	0.03	0.50	0.99
RM572	**0.50**	**0.50**	**1.00**	**0.85**	**0.03**	**0.51**	**1.00**	0.53	0.50	0.99	0.82	0.03	0.50	0.99
RM9	**0.50**	**0.50**	**1.00**	**0.86**	**0.03**	**0.50**	**1.00**	0.51	0.50	0.99	0.83	0.03	0.50	0.99
RM5	**0.50**	**0.50**	**1.00**	**0.85**	**0.03**	**0.51**	**1.00**	**0.50**	**0.50**	**0.99**	**0.83**	**0.03**	**0.50**	**0.99**
RM212	0.53	0.50	1.00	0.86	0.03	0.50	1.00	0.52	0.50	0.99	0.83	0.03	0.50	0.99
RM6895	**0.50**	**0.50**	**1.00**	**0.86**	**0.03**	**0.50**	**1.00**	**0.50**	**0.50**	**0.99**	**0.83**	**0.03**	**0.50**	**0.99**
RM7245	0.50	0.50	1.00	0.85	0.03	0.50	1.00	**0.50**	**0.50**	**0.99**	**0.83**	**0.03**	**0.50**	**0.99**
RM523	**0.50**	**0.50**	**1.00**	**0.84**	**0.03**	**0.50**	**1.00**	**0.50**	**0.50**	**0.99**	**0.84**	**0.03**	**0.50**	**0.99**
RM282	0.53	0.50	1.00	0.86	0.03	0.50	1.00	0.55	0.50	0.99	0.82	0.03	0.50	0.99
RM16	0.52	0.50	1.00	0.85	0.03	0.50	1.00	0.51	0.50	0.99	0.83	0.03	0.50	0.99
RM186	0.58	0.50	1.00	0.86	0.03	0.50	1.00	0.54	0.50	0.99	0.81	0.03	0.50	0.99
RM468	0.56	0.50	1.00	0.86	0.03	0.50	1.00	0.57	0.50	0.99	0.83	0.03	0.50	0.99

RM6395	**0.50**	**0.50**	**1.00**	**0.86**	**0.03**	**0.50**	**1.00**	**0.50**	**0.50**	**0.99**	**0.83**	**0.03**	**0.50**	**0.99**
RM6089	**0.50**	**0.50**	**1.00**	**0.86**	**0.03**	**0.50**	**1.00**	0.50	0.50	0.99	0.83	0.03	0.50	0.99
RM3558	**0.50**	**0.50**	**1.00**	**0.86**	**0.03**	**0.51**	**1.00**	0.51	0.50	0.99	0.83	0.03	0.50	0.99
RM6748	**0.50**	**0.50**	**1.00**	**0.86**	**0.03**	**0.51**	**1.00**	**0.50**	**0.50**	**0.99**	**0.83**	**0.03**	**0.50**	**0.99**
RM3419	0.51	0.50	1.00	0.85	0.03	0.50	1.00	0.51	0.50	0.99	0.83	0.03	0.50	0.99
RM6841	0.59	0.50	1.00	0.87	0.02	0.51	1.00	0.58	0.50	0.99	0.84	0.03	0.50	0.99
RM190	0.67	0.50	1.00	0.86	0.03	0.50	1.00	0.62	0.50	0.99	0.83	0.03	0.51	0.99
RM276	**0.50**	**0.50**	**1.00**	**0.86**	**0.03**	**0.50**	**1.00**	**0.50**	**0.50**	**0.99**	**0.83**	**0.03**	**0.50**	**0.99**
RM214	0.54	0.50	1.00	0.85	0.03	0.50	1.00	0.75	0.50	0.99	0.83	0.03	0.50	0.99
RM11	**0.50**	**0.50**	**1.00**	**0.84**	**0.03**	**0.50**	**1.00**	0.58	0.50	0.99	0.82	0.03	0.50	0.99
RM505	0.52	0.50	1.00	0.85	0.03	0.50	1.00	**0.50**	**0.50**	**0.99**	**0.84**	**0.03**	**0.51**	**0.99**
RM172	**0.50**	**0.50**	**1.00**	**0.85**	**0.03**	**0.51**	**1.00**	0.51	0.50	0.99	0.83	0.03	0.50	0.99
RM408	-	-	-	-	-	-	-	-	-	-	-	-	-	-
RM5068	0.70	0.50	1.00	0.86	0.03	0.50	1.00	0.86	0.50	0.99	0.83	0.03	0.50	0.99
RM331	0.52	0.50	1.00	0.86	0.03	0.50	1.00	0.53	0.50	0.99	0.83	0.03	0.50	0.99
RM296	0.51	0.50	1.00	0.86	0.03	0.50	1.00	0.52	0.50	0.99	0.83	0.03	0.50	0.99
RM4455	0.53	0.50	1.00	0.85	0.03	0.50	1.00	0.51	0.50	0.99	0.83	0.03	0.50	0.99
RM7557	0.51	0.50	1.00	0.86	0.03	0.50	1.00	0.53	0.50	0.99	0.82	0.03	0.50	0.99
RM332	**0.50**	**0.50**	**1.00**	**0.86**	**0.03**	**0.50**	**1.00**	0.52	0.50	0.99	0.83	0.03	0.50	0.99
RM4862	0.72	0.50	1.00	0.86	0.03	0.50	1.00	0.65	0.50	0.99	0.83	0.03	0.50	0.99
RM224	0.54	0.50	1.00	0.86	0.03	0.50	1.00	0.53	0.50	0.99	0.83	0.03	0.50	0.99
RM8216	0.50	0.50	1.00	0.86	0.03	0.50	1.00	**0.50**	**0.50**	**0.99**	**0.84**	**0.03**	**0.50**	**0.99**
RM3483	0.51	0.50	1.00	0.85	0.03	0.50	1.00	0.50	0.50	0.99	0.82	0.03	0.50	0.99
RM6296	**0.50**	**0.50**	**1.00**	**0.86**	**0.03**	**0.50**	**1.00**	0.51	0.50	0.99	0.83	0.03	0.50	0.99
RM277	**0.50**	**0.50**	**1.00**	**0.85**	**0.03**	**0.51**	**1.00**	0.50	0.50	0.99	0.84	0.03	0.50	0.99
RM6732	0.57	0.50	1.00	0.85	0.03	0.51	1.00	0.66	0.50	0.99	0.82	0.03	0.50	0.99

RM17	0.56	0.50	1.00	0.86	0.03	0.50	1.00	0.55	0.50	0.99	0.84	0.03	0.50	0.99
RM111	0.52	0.50	0.98	0.81	0.03	0.50	0.98	0.51	0.50	0.92	0.74	0.02	0.50	0.92
RM143	0.51	0.50	1.00	0.86	0.03	0.50	1.00	0.51	0.50	0.99	0.82	0.03	0.50	0.99
RM333	**0.50**	**0.50**	**1.00**	**0.85**	**0.03**	**0.50**	**1.00**	**0.50**	**0.50**	**0.99**	**0.82**	**0.03**	**0.50**	**0.99**
RM4128	**0.50**	**0.50**	**1.00**	**0.85**	**0.03**	**0.50**	**1.00**	**0.50**	**0.50**	**0.99**	**0.83**	**0.03**	**0.50**	**0.99**
RM168	0.56	0.50	1.00	0.85	0.03	0.50	1.00	0.58	0.50	0.99	0.83	0.03	0.50	0.99
RM565	0.55	0.50	1.00	0.86	0.03	0.50	1.00	0.54	0.50	0.99	0.83	0.03	0.50	0.99
RM16074	**0.50**	**0.50**	**1.00**	**0.86**	**0.03**	**0.50**	**1.00**	**0.50**	**0.50**	**0.99**	**0.83**	**0.03**	**0.50**	**0.99**
RM20468	**0.50**	**0.50**	**1.00**	**0.85**	**0.03**	**0.51**	**1.00**	0.50	0.50	0.99	0.83	0.03	0.50	0.99
RM130	**0.50**	**0.50**	**1.00**	**0.86**	**0.03**	**0.50**	**1.00**	**0.50**	**0.50**	**0.99**	**0.84**	**0.03**	**0.50**	**0.99**
RM20460	**0.50**	**0.50**	**1.00**	**0.86**	**0.03**	**0.50**	**1.00**	0.50	0.50	0.99	0.84	0.03	0.50	0.99
RM5371	0.51	0.50	1.00	0.87	0.03	0.51	1.00	**0.50**	**0.50**	**0.99**	**0.83**	**0.03**	**0.50**	**0.99**
RM16071	0.51	0.50	1.00	0.86	0.03	0.51	1.00	0.51	0.50	0.99	0.84	0.03	0.51	0.99
				F_3 WM-RM408							F_3 non-WM-RM408			
	Obs.F	Min F	Max F	Mean*	SE*	L95*	U95*	Obs. F	Min F	Max F	Mean*	SE*	L95*	U95*
RM84	0.54	0.50	1.00	0.85	0.03	0.50	1.00	0.50	0.50	0.99	0.84	0.03	0.50	0.99
RM575	0.52	0.50	1.00	0.85	0.03	0.51	1.00	0.51	0.50	0.99	0.83	0.03	0.50	0.99
RM572	**0.50**	**0.50**	**1.00**	**0.86**	**0.03**	**0.50**	**1.00**	**0.50**	**0.50**	**0.99**	**0.84**	**0.03**	**0.51**	**0.99**
RM9	**0.50**	**0.50**	**1.00**	**0.85**	**0.03**	**0.51**	**1.00**	**0.50**	**0.50**	**0.99**	**0.83**	**0.03**	**0.50**	**0.99**
RM5	**0.50**	**0.50**	**1.00**	**0.85**	**0.03**	**0.50**	**1.00**	**0.50**	**0.50**	**0.99**	**0.84**	**0.03**	**0.50**	**0.99**
RM212	0.54	0.50	1.00	0.85	0.03	0.51	1.00	0.52	0.50	0.99	0.83	0.03	0.50	0.99
RM6895	**0.50**	**0.50**	**1.00**	**0.86**	**0.03**	**0.50**	**1.00**	**0.50**	**0.50**	**0.99**	**0.85**	**0.03**	**0.50**	**0.99**
RM7245	**0.50**	**0.50**	**1.00**	**0.85**	**0.03**	**0.51**	**1.00**	**0.50**	**0.50**	**0.99**	**0.84**	**0.03**	**0.51**	**0.99**
RM523	**0.50**	**0.50**	**0.99**	**0.85**	**0.03**	**0.51**	**0.99**	**0.50**	**0.50**	**0.99**	**0.83**	**0.03**	**0.50**	**0.99**
RM282	0.53	0.50	0.99	0.84	0.03	0.50	0.99	0.55	0.50	0.99	0.82	0.03	0.50	0.99
RM16	0.52	0.50	1.00	0.85	0.03	0.51	1.00	0.51	0.50	0.99	0.83	0.03	0.50	0.99

RM186	0.57	0.50	0.99	0.85	0.03	0.50	0.99	0.59	0.50	0.99	0.83	0.03	0.50	0.99
RM468	0.55	0.50	1.00	0.86	0.03	0.51	1.00	0.58	0.50	0.99	0.84	0.03	0.50	0.99
RM6395	**0.50**	**0.50**	**1.00**	**0.86**	**0.03**	**0.50**	**1.00**	**0.50**	**0.50**	**0.99**	**0.83**	**0.03**	**0.51**	**0.99**
RM6089	**0.50**	**0.50**	**1.00**	**0.84**	**0.03**	**0.50**	**1.00**	**0.50**	**0.50**	**0.99**	**0.84**	**0.03**	**0.50**	**0.99**
RM3558	**0.50**	**0.50**	**1.00**	**0.85**	**0.03**	**0.50**	**1.00**	**0.50**	**0.50**	**0.99**	**0.84**	**0.03**	**0.50**	**0.99**
RM6748	**0.50**	**0.50**	**1.00**	**0.86**	**0.03**	**0.50**	**1.00**	0.51	0.50	0.99	0.83	0.03	0.50	0.99
RM3419	0.51	0.50	0.99	0.84	0.03	0.50	0.99	**0.50**	**0.50**	**0.99**	**0.83**	**0.03**	**0.50**	**0.99**
RM6841	0.60	0.50	1.00	0.84	0.03	0.50	1.00	0.56	0.50	0.99	0.84	0.03	0.50	0.99
RM190	0.65	0.50	1.00	0.86	0.03	0.51	1.00	0.69	0.50	0.99	0.83	0.03	0.50	0.99
RM276	0.50	0.50	1.00	0.86	0.03	0.50	1.00	0.51	0.50	0.99	0.83	0.03	0.50	0.99
RM214	0.56	0.50	1.00	0.85	0.03	0.50	1.00	0.56	0.50	0.99	0.84	0.03	0.50	0.99
RM11	**0.50**	**0.50**	**1.00**	**0.85**	**0.03**	**0.50**	**1.00**	0.52	0.50	0.99	0.82	0.03	0.50	0.99
RM505	0.51	0.50	1.00	0.85	0.03	0.50	1.00	0.51	0.50	0.99	0.84	0.03	0.50	0.99
RM172	**0.50**	**0.50**	**1.00**	**0.85**	**0.03**	**0.50**	**1.00**	**0.50**	**0.50**	**0.99**	**0.84**	**0.03**	**0.51**	**0.99**
RM408	-	-	-	-	-	-	-	-	-	-	-	-	-	-
RM5068	0.77	0.50	1.00	0.84	0.03	0.50	1.00	0.63	0.50	0.99	0.83	0.03	0.50	0.99
RM331	0.52	0.50	1.00	0.86	0.03	0.50	1.00	0.51	0.50	0.99	0.84	0.03	0.50	0.99
RM296	0.51	0.50	1.00	0.85	0.03	0.50	1.00	0.51	0.50	0.99	0.83	0.03	0.50	0.99
RM4455	0.52	0.50	1.00	0.85	0.03	0.50	1.00	0.53	0.50	0.99	0.83	0.03	0.50	0.99
RM7557	0.51	0.50	1.00	0.85	0.03	0.50	1.00	0.51	0.50	0.99	0.83	0.03	0.50	0.99
RM332	**0.50**	**0.50**	**1.00**	**0.85**	**0.03**	**0.51**	**1.00**	**0.50**	**0.50**	**0.99**	**0.84**	**0.03**	**0.50**	**0.99**
RM4862	0.68	0.50	1.00	0.86	0.03	0.50	1.00	0.78	0.50	0.99	0.83	0.03	0.50	0.99
RM224	0.55	0.50	1.00	0.85	0.03	0.50	1.00	0.52	0.50	0.99	0.83	0.03	0.50	0.99
RM8216	**0.50**	**0.50**	**1.00**	**0.84**	**0.03**	**0.50**	**1.00**	0.51	0.50	0.99	0.83	0.03	0.50	0.99
RM3483	0.51	0.50	1.00	0.86	0.03	0.50	1.00	**0.50**	**0.50**	**0.99**	**0.83**	**0.03**	**0.50**	**0.99**
RM6296	**0.50**	**0.50**	**1.00**	**0.86**	**0.03**	**0.50**	**1.00**	0.51	0.50	0.99	0.84	0.03	0.50	0.99

RM277	0.50	0.50	1.00	0.86	0.03	0.50	1.00	**0.50**	**0.50**	**0.99**	**0.83**	**0.03**	**0.50**	**0.99**
RM6732	0.58	0.50	1.00	0.86	0.03	0.50	1.00	0.58	0.50	0.99	0.84	0.03	0.51	0.99
RM17	0.55	0.50	1.00	0.86	0.03	0.50	1.00	0.58	0.50	0.99	0.83	0.03	0.50	0.99
RM111	0.53	0.50	0.98	0.80	0.03	0.50	0.98	0.51	0.50	0.95	0.78	0.03	0.50	0.95
RM143	0.51	0.50	1.00	0.85	0.03	0.50	1.00	0.50	0.50	0.99	0.83	0.03	0.50	0.99
RM333	**0.50**	**0.50**	**1.00**	**0.86**	**0.03**	**0.51**	**1.00**	0.51	0.50	0.99	0.84	0.03	0.51	0.99
RM4128	0.51	0.50	1.00	0.85	0.03	0.50	1.00	0.52	0.50	0.99	0.83	0.03	0.50	0.99
RM168	0.55	0.50	1.00	0.86	0.03	0.50	1.00	0.59	0.50	0.99	0.84	0.03	0.50	0.99
RM565	0.54	0.50	1.00	0.86	0.03	0.51	1.00	0.55	0.50	0.99	0.85	0.03	0.50	0.99
RM16074	**0.50**	**0.50**	**1.00**	**0.86**	**0.03**	**0.50**	**1.00**	**0.50**	**0.50**	**0.99**	**0.83**	**0.03**	**0.50**	**0.99**
RM20468	**0.50**	**0.50**	**1.00**	**0.85**	**0.03**	**0.50**	**1.00**	**0.50**	**0.50**	**0.99**	**0.84**	**0.03**	**0.51**	**0.99**
RM130	**0.50**	**0.50**	**1.00**	**0.84**	**0.03**	**0.50**	**1.00**	**0.50**	**0.50**	**0.99**	**0.83**	**0.03**	**0.50**	**0.99**
RM20460	**0.50**	**0.50**	**1.00**	**0.85**	**0.03**	**0.50**	**1.00**	**0.50**	**0.50**	**0.99**	**0.84**	**0.03**	**0.51**	**0.99**
RM5371	**0.51**	**0.50**	**1.00**	**0.85**	**0.03**	**0.51**	**1.00**	0.51	0.50	0.99	0.84	0.03	0.50	0.99
RM16071	0.51	0.50	1.00	0.84	0.03	0.50	1.00	**0.50**	**0.50**	**0.99**	**0.84**	**0.03**	**0.50**	**0.99**

	F_3 CM-RM277							F_3 non-CM-RM277						
	Obs.F	Min F	Max F	Mean*	SE*	L95*	U95*	Obs. F	Min F	Max F	Mean*	SE*	L95*	U95*
RM84	0.53	0.50	1.00	0.86	0.03	0.50	1.00	0.50	0.50	0.99	0.83	0.03	0.50	0.99
RM575	0.52	0.50	1.00	0.86	0.03	0.51	1.00	0.52	0.50	0.99	0.83	0.03	0.50	0.99
RM572	**0.50**	**0.50**	**1.00**	**0.86**	**0.03**	**0.50**	**1.00**	**0.50**	**0.50**	**0.99**	**0.82**	**0.03**	**0.50**	**0.99**
RM9	**0.50**	**0.50**	**1.00**	**0.85**	**0.03**	**0.50**	**1.00**	0.52	0.50	0.98	0.82	0.03	0.50	0.98
RM5	**0.50**	**0.50**	**1.00**	**0.86**	**0.03**	**0.51**	**1.00**	**0.50**	**0.50**	**0.99**	**0.83**	**0.03**	**0.50**	**0.99**
RM212	0.53	0.50	1.00	0.86	0.03	0.50	1.00	0.53	0.50	0.99	0.82	0.03	0.50	0.99
RM6895	**0.50**	**0.50**	**1.00**	**0.86**	**0.03**	**0.50**	**1.00**	0.51	0.50	0.99	0.82	0.03	0.50	0.99
RM7245	0.50	0.50	1.00	0.85	0.03	0.50	1.00	**0.50**	**0.50**	**0.99**	**0.82**	**0.03**	**0.50**	**0.99**
RM523	**0.50**	**0.50**	**1.00**	**0.85**	**0.03**	**0.50**	**1.00**	**0.50**	**0.50**	**0.98**	**0.81**	**0.03**	**0.50**	**0.98**

RM282	0.53	0.50	1.00	0.84	0.03	0.50	1.00	0.54	0.50	0.98	0.80	0.03	0.50	0.98
RM16	0.51	0.50	1.00	0.86	0.03	0.50	1.00	0.52	0.50	0.99	0.82	0.03	0.50	0.99
RM186	0.58	0.50	1.00	0.85	0.03	0.50	1.00	0.57	0.50	0.98	0.81	0.03	0.50	0.98
RM468	0.57	0.50	1.00	0.85	0.03	0.50	1.00	0.55	0.50	0.99	0.82	0.03	0.50	0.99
RM6395	**0.50**	**0.50**	**1.00**	**0.86**	**0.03**	**0.51**	**1.00**	**0.50**	**0.50**	**0.99**	**0.83**	**0.03**	**0.51**	**0.99**
RM6089	**0.50**	**0.50**	**1.00**	**0.86**	**0.03**	**0.50**	**1.00**	0.53	0.50	0.99	0.82	0.03	0.50	0.99
RM3558	**0.50**	**0.50**	**1.00**	**0.85**	**0.03**	**0.50**	**1.00**	0.52	0.50	0.99	0.82	0.03	0.50	0.99
RM6748	**0.50**	**0.50**	**1.00**	**0.86**	**0.03**	**0.50**	**1.00**	0.54	0.50	0.99	0.83	0.03	0.50	0.99
RM3419	0.51	0.50	1.00	0.86	0.03	0.51	1.00	**0.50**	**0.50**	**0.98**	**0.82**	**0.03**	**0.50**	**0.98**
RM6841	0.59	0.50	1.00	0.85	0.03	0.50	1.00	0.56	0.50	0.99	0.82	0.03	0.50	0.99
RM190	0.65	0.50	1.00	0.86	0.03	0.50	1.00	0.73	0.50	0.99	0.82	0.03	0.50	0.99
RM276	**0.50**	**0.50**	**1.00**	**0.85**	**0.03**	**0.50**	**1.00**	**0.50**	**0.50**	**0.99**	**0.82**	**0.03**	**0.50**	**0.99**
RM214	0.56	0.50	1.00	0.86	0.03	0.50	1.00	0.58	0.50	0.99	0.82	0.03	0.50	0.99
RM11	**0.50**	**0.50**	**1.00**	**0.85**	**0.03**	**0.51**	**1.00**	0.54	0.50	0.98	0.81	0.03	0.50	0.98
RM505	0.52	0.50	1.00	0.86	0.03	0.50	1.00	0.51	0.50	0.99	0.82	0.03	0.50	0.99
RM172	**0.50**	**0.50**	**1.00**	**0.85**	**0.03**	**0.50**	**1.00**	**0.50**	**0.50**	**0.99**	**0.81**	**0.03**	**0.50**	**0.99**
RM408	0.51	0.50	1.00	0.86	0.03	0.51	1.00	0.55	0.50	0.99	0.82	0.03	0.50	0.99
RM5068	0.72	0.50	1.00	0.86	0.03	0.51	1.00	0.71	0.50	0.99	0.82	0.03	0.51	0.99
RM331	0.52	0.50	1.00	0.86	0.03	0.50	1.00	0.50	0.50	0.99	0.82	0.03	0.50	0.99
RM296	0.51	0.50	1.00	0.86	0.03	0.50	1.00	0.51	0.50	0.99	0.82	0.03	0.50	0.99
RM4455	0.52	0.50	1.00	0.85	0.03	0.50	1.00	0.55	0.50	0.99	0.82	0.03	0.50	0.99
RM7557	0.51	0.50	1.00	0.85	0.03	0.50	1.00	0.53	0.50	0.98	0.81	0.03	0.50	0.98
RM332	**0.50**	**0.50**	**1.00**	**0.85**	**0.03**	**0.50**	**1.00**	**0.50**	**0.50**	**0.99**	**0.82**	**0.03**	**0.50**	**0.99**
RM4862	0.71	0.50	1.00	0.86	0.03	0.50	1.00	0.72	0.50	0.99	0.82	0.03	0.50	0.99
RM224	0.54	0.50	1.00	0.86	0.03	0.51	1.00	0.53	0.50	0.99	0.83	0.03	0.50	0.99
RM8216	**0.50**	**0.50**	**1.00**	**0.85**	**0.03**	**0.50**	**1.00**	0.51	0.50	0.99	0.81	0.03	0.50	0.99

RM3483	0.51	0.50	1.00	0.86	0.03	0.51	1.00	**0.50**	**0.50**	**0.99**	**0.83**	**0.03**	**0.51**	**0.99**
RM6296	**0.50**	**0.50**	**1.00**	**0.86**	**0.03**	**0.51**	**1.00**	0.60	0.50	0.99	0.82	0.03	0.50	0.99
RM277	-	-	-	-	-	-	-	-	-	-	-	-	-	-
RM6732	0.60	0.50	1.00	0.86	0.03	0.51	1.00	0.53	0.50	0.99	0.82	0.03	0.50	0.99
RM17	0.58	0.50	1.00	0.85	0.03	0.50	1.00	0.51	0.50	0.99	0.83	0.03	0.50	0.99
RM111	0.55	0.50	0.98	0.81	0.03	0.50	0.98	0.51	0.50	0.94	0.76	0.03	0.50	0.94
RM143	0.51	0.50	1.00	0.85	0.03	0.51	1.00	0.51	0.50	0.99	0.82	0.03	0.50	0.99
RM333	**0.50**	**0.50**	**1.00**	**0.86**	**0.03**	**0.50**	**1.00**	0.51	0.50	0.99	0.82	0.03	0.50	0.99
RM4128	**0.50**	**0.50**	**1.00**	**0.85**	**0.03**	**0.51**	**1.00**	**0.50**	**0.50**	**0.99**	**0.82**	**0.03**	**0.50**	**0.99**
RM168	0.56	0.50	1.00	0.86	0.03	0.50	1.00	0.61	0.50	0.99	0.83	0.03	0.50	0.99
RM565	0.55	0.50	1.00	0.86	0.03	0.50	1.00	0.54	0.50	0.99	0.83	0.03	0.50	0.99
RM16074	**0.50**	**0.50**	**1.00**	**0.86**	**0.03**	**0.50**	**1.00**	**0.50**	**0.50**	**0.99**	**0.82**	**0.03**	**0.50**	**0.99**
RM20468	**0.50**	**0.50**	**1.00**	**0.86**	**0.03**	**0.50**	**1.00**	0.50	0.50	0.99	0.82	0.03	0.50	0.99
RM130	**0.50**	**0.50**	**1.00**	**0.85**	**0.03**	**0.51**	**1.00**	**0.50**	**0.50**	**0.99**	**0.81**	**0.03**	**0.50**	**0.99**
RM20460	**0.50**	**0.50**	**1.00**	**0.87**	**0.03**	**0.50**	**1.00**	**0.50**	**0.50**	**0.99**	**0.83**	**0.03**	**0.50**	**0.99**
RM5371	0.51	0.50	1.00	0.85	0.03	0.50	1.00	0.50	0.50	0.99	0.82	0.03	0.50	0.99
RM16071	0.51	0.50	1.00	0.86	0.03	0.50	1.00	0.51	0.50	0.99	0.82	0.03	0.50	0.99
	F_3 WM-RM277							F_3 non-WM-RM277						
	Obs.F	Min F	Max F	Mean*	SE*	L95*	U95*	Obs. F	Min F	Max F	Mean*	SE*	L95*	U95*
RM84	0.52	0.50	1.00	0.85	0.03	0.50	1.00	0.52	0.50	0.99	0.83	0.03	0.50	0.99
RM575	0.51	0.50	1.00	0.84	0.03	0.50	1.00	0.52	0.50	0.99	0.83	0.03	0.50	0.99
RM572	**0.50**	**0.50**	**1.00**	**0.85**	**0.03**	**0.50**	**1.00**	**0.50**	**0.50**	**0.99**	**0.82**	**0.03**	**0.50**	**0.99**
RM9	0.50	0.50	1.00	0.85	0.03	0.50	1.00	**0.50**	**0.50**	**0.99**	**0.81**	**0.03**	**0.50**	**0.99**
RM5	**0.50**	**0.50**	**1.00**	**0.85**	**0.03**	**0.51**	**1.00**	**0.50**	**0.50**	**0.99**	**0.83**	**0.03**	**0.50**	**0.99**
RM212	0.53	0.50	1.00	0.86	0.03	0.50	1.00	0.54	0.50	0.99	0.82	0.03	0.50	0.99
RM6895	**0.50**	**0.50**	**1.00**	**0.85**	**0.03**	**0.51**	**1.00**	0.51	0.50	0.99	0.82	0.03	0.50	0.99

RM7245	**0.50**	**0.50**	**1.00**	**0.85**	**0.03**	**0.50**	**1.00**	0.51	0.50	0.99	0.83	0.03	0.50	0.99
RM523	**0.50**	**0.50**	**1.00**	**0.85**	**0.03**	**0.51**	**1.00**	0.50	0.50	0.99	0.81	0.03	0.50	0.99
RM282	0.53	0.50	1.00	0.85	0.03	0.50	1.00	0.55	0.50	0.98	0.81	0.03	0.50	0.98
RM16	0.51	0.50	1.00	0.85	0.03	0.50	1.00	0.53	0.50	0.99	0.83	0.03	0.50	0.99
RM186	0.59	0.50	1.00	0.85	0.03	0.51	1.00	0.53	0.50	0.98	0.80	0.03	0.50	0.98
RM468	0.57	0.50	1.00	0.86	0.03	0.50	1.00	0.54	0.50	0.99	0.83	0.03	0.50	0.99
RM6395	**0.50**	**0.50**	**1.00**	**0.85**	**0.03**	**0.50**	**1.00**	0.51	0.50	0.99	0.83	0.03	0.50	0.99
RM6089	**0.50**	**0.50**	**1.00**	**0.85**	**0.03**	**0.51**	**1.00**	0.50	0.50	0.99	0.83	0.03	0.50	0.99
RM3558	**0.50**	**0.50**	**1.00**	**0.86**	**0.03**	**0.50**	**1.00**	**0.50**	**0.50**	**0.99**	**0.83**	**0.03**	**0.50**	**0.99**
RM6748	**0.50**	**0.50**	**1.00**	**0.86**	**0.03**	**0.50**	**1.00**	**0.50**	**0.50**	**0.99**	**0.83**	**0.03**	**0.51**	**0.99**
RM3419	0.51	0.50	1.00	0.84	0.03	0.50	1.00	**0.50**	**0.50**	**0.99**	**0.82**	**0.03**	**0.50**	**0.99**
RM6841	0.59	0.50	1.00	0.85	0.03	0.50	1.00	0.57	0.50	0.99	0.82	0.03	0.50	0.99
RM190	0.68	0.50	1.00	0.85	0.03	0.51	1.00	0.62	0.50	0.99	0.84	0.03	0.50	0.99
RM276	**0.50**	**0.50**	**1.00**	**0.86**	**0.03**	**0.50**	**1.00**	**0.50**	**0.50**	**0.99**	**0.83**	**0.03**	**0.50**	**0.99**
RM214	0.56	0.50	1.00	0.86	0.03	0.50	1.00	0.56	0.50	0.99	0.83	0.03	0.50	0.99
RM11	0.51	0.50	1.00	0.85	0.03	0.51	1.00	0.50	0.50	0.99	0.82	0.03	0.50	0.99
RM505	0.51	0.50	1.00	0.85	0.03	0.50	1.00	0.52	0.50	0.99	0.82	0.03	0.50	0.99
RM172	**0.50**	**0.50**	**1.00**	**0.86**	**0.03**	**0.50**	**1.00**	**0.50**	**0.50**	**0.99**	**0.82**	**0.03**	**0.50**	**0.99**
RM408	0.52	0.50	1.00	0.85	0.03	0.51	1.00	0.51	0.50	0.99	0.82	0.03	0.50	0.99
RM5068	0.71	0.50	1.00	0.85	0.03	0.50	1.00	0.77	0.50	0.99	0.82	0.03	0.50	0.99
RM331	0.52	0.50	1.00	0.87	0.03	0.50	1.00	0.51	0.50	0.99	0.83	0.03	0.50	0.99
RM296	0.51	0.50	1.00	0.86	0.03	0.50	1.00	0.51	0.50	0.99	0.83	0.03	0.50	0.99
RM4455	0.53	0.50	1.00	0.86	0.03	0.50	1.00	0.51	0.50	0.99	0.83	0.03	0.50	0.99
RM7557	0.51	0.50	1.00	0.85	0.03	0.50	1.00	**0.50**	**0.50**	**0.99**	**0.81**	**0.03**	**0.50**	**0.99**
RM332	0.50	0.50	1.00	0.86	0.03	0.50	1.00	**0.50**	**0.50**	**0.99**	**0.83**	**0.03**	**0.50**	**0.99**
RM4862	0.71	0.50	1.00	0.85	0.03	0.50	1.00	0.72	0.50	0.99	0.83	0.03	0.50	0.99

RM224	0.54	0.50	1.00	0.85	0.03	0.51	1.00	0.52	0.50	0.99	0.82	0.03	0.50	0.99
RM8216	0.50	0.50	1.00	0.86	0.03	0.50	1.00	0.50	0.50	0.99	0.83	0.03	0.50	0.99
RM3483	**0.50**	**0.50**	**1.00**	**0.85**	**0.03**	**0.50**	**1.00**	0.54	0.50	0.99	0.83	0.03	0.50	0.99
RM6296	0.52	0.50	1.00	0.86	0.02	0.51	1.00	0.54	0.50	0.99	0.82	0.03	0.50	0.99
RM277	-	-	-	-	-	-	-	-	-	-	-	-	-	-
RM6732	0.57	0.50	1.00	0.86	0.03	0.51	1.00	0.63	0.50	0.99	0.83	0.03	0.50	0.99
RM17	0.57	0.50	1.00	0.86	0.03	0.51	1.00	0.54	0.50	0.99	0.82	0.03	0.50	0.99
RM111	0.52	0.50	0.98	0.80	0.03	0.50	0.98	0.56	0.50	0.94	0.75	0.03	0.50	0.94
RM143	0.51	0.50	1.00	0.85	0.03	0.51	1.00	0.51	0.50	0.99	0.83	0.03	0.50	0.99
RM333	**0.50**	**0.50**	**1.00**	**0.86**	**0.03**	**0.50**	**1.00**	**0.50**	**0.50**	**0.99**	**0.82**	**0.03**	**0.50**	**0.99**
RM4128	**0.50**	**0.50**	**1.00**	**0.86**	**0.03**	**0.50**	**1.00**	0.50	0.50	0.99	0.83	0.03	0.50	0.99
RM168	0.59	0.50	1.00	0.85	0.03	0.50	1.00	0.52	0.50	0.99	0.82	0.03	0.50	0.99
RM565	0.55	0.50	1.00	0.86	0.03	0.50	1.00	0.54	0.50	0.99	0.82	0.03	0.50	0.99
RM16074	**0.50**	**0.50**	**1.00**	**0.85**	**0.03**	**0.50**	**1.00**	**0.50**	**0.50**	**0.99**	**0.82**	**0.03**	**0.50**	**0.99**
RM20468	**0.50**	**0.50**	**1.00**	**0.85**	**0.03**	**0.51**	**1.00**	**0.50**	**0.50**	**0.99**	**0.82**	**0.03**	**0.50**	**0.99**
RM130	**0.50**	**0.50**	**1.00**	**0.85**	**0.03**	**0.50**	**1.00**	**0.50**	**0.50**	**0.99**	**0.83**	**0.03**	**0.50**	**0.99**
RM20460	**0.50**	**0.50**	**1.00**	**0.85**	**0.03**	**0.50**	**1.00**	**0.50**	**0.50**	**0.99**	**0.82**	**0.03**	**0.50**	**0.99**
RM5371	**0.50**	**0.50**	**1.00**	**0.86**	**0.03**	**0.51**	**1.00**	0.51	0.50	0.99	0.83	0.03	0.50	0.99
RM16071	**0.51**	**0.50**	**1.00**	**0.85**	**0.03**	**0.51**	**1.00**	0.51	0.50	0.99	0.82	0.03	0.50	0.99

	F_3 Ideal-1							F_3 Ideal-2						
	Obs. F	Min F	Max F	Mean*	SE*	L95*	U95*	Obs. F	Min F	Max F	Mean*	SE*	L95*	U95*
RM84	0.54	0.50	1.00	0.89	0.02	0.51	1.00	0.54	0.50	1.00	0.88	0.03	0.50	1.00
RM575	**0.50**	**0.50**	**1.00**	**0.90**	**0.02**	**0.51**	**1.00**	**0.50**	**0.50**	**1.00**	**0.89**	**0.03**	**0.51**	**1.00**
RM572	**0.50**	**0.50**	**1.00**	**0.89**	**0.02**	**0.51**	**1.00**	**0.50**	**0.50**	**1.00**	**0.89**	**0.02**	**0.51**	**1.00**
RM9	**0.51**	**0.50**	**1.00**	**0.90**	**0.02**	**0.51**	**1.00**	**0.51**	**0.50**	**1.00**	**0.89**	**0.02**	**0.51**	**1.00**
RM5	**0.50**	**0.50**	**1.00**	**0.90**	**0.02**	**0.51**	**1.00**	**0.50**	**0.50**	**1.00**	**0.89**	**0.02**	**0.51**	**1.00**

RM212	0.51	0.50	1.00	0.89	0.03	0.51	1.00	0.52	0.50	1.00	0.89	0.03	0.51	1.00
RM6895	0.55	0.50	1.00	0.90	0.02	0.51	1.00	0.54	0.50	1.00	0.89	0.03	0.51	1.00
RM7245	0.51	0.50	1.00	0.90	0.02	0.51	1.00	**0.51**	**0.50**	**1.00**	**0.89**	**0.02**	**0.51**	**1.00**
RM523	0.53	0.50	1.00	0.90	0.02	0.51	1.00	0.53	0.50	1.00	0.89	0.02	0.51	1.00
RM282	0.60	0.50	1.00	0.89	0.02	0.51	1.00	0.59	0.50	1.00	0.88	0.03	0.51	1.00
RM16	0.53	0.50	1.00	0.89	0.02	0.51	1.00	0.53	0.50	1.00	0.88	0.03	0.50	1.00
RM186	0.59	0.50	1.00	0.89	0.03	0.51	1.00	0.57	0.50	1.00	0.89	0.03	0.51	1.00
RM468	0.56	0.50	1.00	0.88	0.03	0.50	1.00	0.56	0.50	1.00	0.88	0.03	0.50	1.00
RM565	0.54	0.50	1.00	0.90	0.02	0.51	1.00	0.53	0.50	1.00	0.89	0.02	0.51	1.00
RM6089	**0.50**	**0.50**	**1.00**	**0.89**	**0.03**	**0.51**	**1.00**	**0.50**	**0.50**	**1.00**	**0.90**	**0.02**	**0.51**	**1.00**
RM3558	0.51	0.50	1.00	0.88	0.03	0.50	1.00	0.51	0.50	1.00	0.89	0.03	0.50	1.00
RM6748	**0.50**	**0.50**	**1.00**	**0.89**	**0.02**	**0.51**	**1.00**	**0.50**	**0.50**	**1.00**	**0.89**	**0.02**	**0.50**	**1.00**
RM3419	**0.50**	**0.50**	**1.00**	**0.90**	**0.02**	**0.51**	**1.00**	**0.50**	**0.50**	**1.00**	**0.89**	**0.02**	**0.51**	**1.00**
RM6841	0.60	0.50	1.00	0.89	0.02	0.51	1.00	0.59	0.50	1.00	0.89	0.02	0.51	1.00
RM190	0.60	0.50	1.00	0.89	0.03	0.51	1.00	0.60	0.50	1.00	0.88	0.03	0.51	1.00
RM276	**0.50**	**0.50**	**1.00**	**0.88**	**0.03**	**0.51**	**1.00**	**0.50**	**0.50**	**1.00**	**0.88**	**0.03**	**0.50**	**1.00**
RM214	0.58	0.50	1.00	0.89	0.02	0.51	1.00	0.59	0.50	1.00	0.89	0.02	0.50	1.00
RM11	**0.51**	**0.50**	**1.00**	**0.90**	**0.02**	**0.51**	**1.00**	**0.50**	**0.50**	**1.00**	**0.88**	**0.03**	**0.51**	**1.00**
RM505	0.55	0.50	1.00	0.90	0.02	0.51	1.00	0.56	0.50	1.00	0.88	0.02	0.51	1.00
RM172	**0.50**	**0.50**	**1.00**	**0.90**	**0.02**	**0.51**	**1.00**	**0.50**	**0.50**	**1.00**	**0.89**	**0.02**	**0.51**	**1.00**
RM408	**0.50**	**0.50**	**1.00**	**0.89**	**0.03**	**0.51**	**1.00**	**0.50**	**0.50**	**1.00**	**0.88**	**0.03**	**0.51**	**1.00**
RM5068	0.66	0.50	1.00	0.89	0.03	0.51	1.00	0.65	0.50	1.00	0.89	0.03	0.51	1.00
RM331	0.53	0.50	1.00	0.89	0.02	0.51	1.00	0.52	0.50	1.00	0.89	0.02	0.51	1.00
RM296	0.52	0.50	1.00	0.89	0.03	0.51	1.00	0.52	0.50	1.00	0.89	0.03	0.50	1.00
RM4455	0.53	0.50	1.00	0.89	0.02	0.51	1.00	0.52	0.50	1.00	0.89	0.02	0.51	1.00
RM7557	0.50	0.50	1.00	0.89	0.03	0.50	1.00	**0.51**	**0.50**	**1.00**	**0.88**	**0.03**	**0.51**	**1.00**

RM332	0.52	0.50	1.00	0.89	0.03	0.50	1.00	0.52	0.50	1.00	0.89	0.02	0.51	1.00
RM4862	0.67	0.50	1.00	0.89	0.03	0.51	1.00	0.66	0.50	1.00	0.89	0.03	0.51	1.00
RM224	0.51	0.50	1.00	0.89	0.03	0.51	1.00	0.51	0.50	1.00	0.89	0.03	0.51	1.00
RM8216	0.55	0.50	1.00	0.89	0.03	0.51	1.00	0.55	0.50	1.00	0.90	0.02	0.51	1.00
RM3483	0.51	0.50	1.00	0.89	0.03	0.50	1.00	0.52	0.50	1.00	0.88	0.03	0.51	1.00
RM6296	0.56	0.50	1.00	0.90	0.02	0.51	1.00	0.56	0.50	1.00	0.89	0.02	0.51	1.00
RM277	**0.50**	**0.50**	**1.00**	**0.90**	**0.02**	**0.51**	**1.00**	**0.50**	**0.50**	**1.00**	**0.89**	**0.02**	**0.51**	**1.00**
RM6732	0.55	0.50	1.00	0.90	0.02	0.51	1.00	0.55	0.50	1.00	0.88	0.03	0.50	1.00
RM17	**0.50**	**0.50**	**1.00**	**0.89**	**0.02**	**0.51**	**1.00**	**0.51**	**0.50**	**1.00**	**0.89**	**0.02**	**0.51**	**1.00**
RM111	0.53	0.50	1.00	0.90	0.02	0.51	1.00	0.53	0.50	1.00	0.89	0.02	0.50	1.00
RM143	0.50	0.50	1.00	0.80	0.04	0.41	1.00	0.50	0.50	1.00	0.80	0.03	0.44	1.00
RM333	**0.50**	**0.50**	**1.00**	**0.89**	**0.02**	**0.51**	**1.00**	**0.50**	**0.50**	**1.00**	**0.89**	**0.02**	**0.51**	**1.00**
RM4128	0.51	0.50	1.00	0.89	0.02	0.51	1.00	0.51	0.50	1.00	0.89	0.03	0.51	1.00
RM168	0.56	0.50	1.00	0.90	0.02	0.50	1.00	0.55	0.50	1.00	0.89	0.02	0.51	1.00
RM6395	0.53	0.50	1.00	0.89	0.02	0.51	1.00	0.53	0.50	1.00	0.88	0.03	0.51	1.00
RM16074	**0.50**	**0.50**	**1.00**	**0.90**	**0.02**	**0.51**	**1.00**	**0.50**	**0.50**	**1.00**	**0.89**	**0.02**	**0.51**	**1.00**
RM20468	0.51	0.50	1.00	0.89	0.03	0.51	1.00	0.51	0.50	1.00	0.89	0.03	0.51	1.00
RM130	**0.50**	**0.50**	**1.00**	**0.90**	**0.02**	**0.51**	**1.00**	**0.51**	**0.50**	**1.00**	**0.89**	**0.02**	**0.51**	**1.00**
RM20460	0.51	0.50	1.00	0.90	0.02	0.51	1.00	0.51	0.50	1.00	0.89	0.02	0.51	1.00
RM5371	0.52	0.50	1.00	0.89	0.02	0.51	1.00	0.52	0.50	1.00	0.89	0.02	0.51	1.00
RM16071	**0.50**	**0.50**	**1.00**	**0.90**	**0.02**	**0.51**	**1.00**	0.51	0.50	1.00	0.89	0.02	0.51	1.00

* Obs. F indicates observed allele frequencies; Nul. F indicates null allele frequencies; L95 and U95 indicate the lower and upper limits of the 95% confidence interval of Nul. F, respectively. If Obs. F is within this confidence interval, the locus is neutral; otherwise, it is not.

Supplementary Table 4 Results of the neutrality test, showing loci (in bold) whose parental alleles are significantly deviated (*P* <0.05) from the theoretical values in F_2/F_3 crop-weed hybrid lineages with (GE) or without (non-GE) the *epsps* transgene; with (CM) or without (non-CM) the crop-parent markers; and with (WM) or without (non-WM) the weedy-parent markers, using the ideal groups as a reference.

Primer name	F_2 GE							F_2 non-GE						
	Obs. F	Min F	Max F	Mean*	SE*	L95*	U95*	Obs. F	Min F	Max F	Mean*	SE*	L95*	U95*
RM527	0.51	0.50	1.00	0.86	0.03	0.50	1.00	**0.50**	**0.50**	**0.99**	**0.82**	**0.03**	**0.50**	**0.99**
RM23	**0.50**	**0.50**	**1.00**	**0.86**	**0.03**	**0.51**	**1.00**	0.57	0.50	0.99	0.83	0.03	0.50	0.99
RM231	0.52	0.50	1.00	0.85	0.03	0.50	1.00	0.53	0.50	0.99	0.82	0.03	0.50	0.99
RM4862	0.52	0.50	1.00	0.85	0.03	0.50	1.00	0.53	0.50	0.99	0.82	0.03	0.50	0.99
RM447	**0.50**	**0.50**	**1.00**	**0.86**	**0.03**	**0.51**	**1.00**	0.52	0.50	0.99	0.83	0.03	0.50	0.99
RM16	0.51	0.50	1.00	0.85	0.03	0.50	1.00	0.53	0.50	0.99	0.82	0.03	0.50	0.99
RM214	0.55	0.50	1.00	0.85	0.03	0.50	1.00	0.51	0.50	0.99	0.82	0.03	0.50	0.99
RM3533	**0.50**	**0.50**	**1.00**	**0.85**	**0.03**	**0.50**	**1.00**	0.52	0.50	0.99	0.82	0.03	0.50	0.99
RM211	0.51	0.50	1.00	0.85	0.03	0.50	1.00	**0.50**	**0.50**	**0.99**	**0.82**	**0.03**	**0.50**	**0.99**
RM296	0.54	0.50	1.00	0.84	0.03	0.50	1.00	0.51	0.50	0.99	0.81	0.03	0.50	0.99
RM84	0.55	0.50	1.00	0.85	0.03	0.50	1.00	0.50	0.50	0.99	0.83	0.03	0.50	0.99
RM4128	0.58	0.50	1.00	0.85	0.03	0.50	1.00	0.66	0.50	0.99	0.82	0.03	0.50	0.99
RM172	0.50	0.50	1.00	0.85	0.03	0.50	1.00	**0.50**	**0.50**	**0.99**	**0.82**	**0.03**	**0.50**	**0.99**
RM5	0.88	0.50	1.00	0.86	0.03	0.51	1.00	0.75	0.50	0.99	0.82	0.03	0.50	0.99
RM168	0.51	0.50	1.00	0.85	0.03	0.50	1.00	**0.50**	**0.50**	**0.99**	**0.83**	**0.03**	**0.51**	**0.99**
RM186	**0.50**	**0.50**	**1.00**	**0.85**	**0.03**	**0.50**	**1.00**	0.50	0.50	0.99	0.82	0.03	0.50	0.99
RM520	0.54	0.50	1.00	0.86	0.03	0.50	1.00	0.51	0.50	0.99	0.82	0.03	0.50	0.99
RM8277	**0.50**	**0.50**	**1.00**	**0.86**	**0.03**	**0.51**	**1.00**	0.51	0.50	0.99	0.83	0.03	0.50	0.99
RM8243	0.51	0.50	1.00	0.85	0.03	0.50	1.00	0.50	0.50	0.99	0.83	0.03	0.50	0.99
RM532	0.58	0.50	0.99	0.85	0.03	0.50	0.99	0.56	0.50	0.98	0.81	0.03	0.50	0.98

RM128	0.52	0.50	1.00	0.85	0.03	0.50	1.00	0.50	0.50	0.99	0.82	0.03	0.50	0.99
RM8206	0.53	0.50	1.00	0.86	0.03	0.50	1.00	0.51	0.50	0.99	0.82	0.03	0.50	0.99
RM340	**0.50**	**0.50**	**1.00**	**0.85**	**0.03**	**0.51**	**1.00**	0.52	0.50	0.99	0.82	0.03	0.50	0.99
RM587	0.51	0.50	1.00	0.86	0.03	0.50	1.00	**0.50**	**0.50**	**0.99**	**0.82**	**0.03**	**0.50**	**0.99**
RM530	0.52	0.50	1.00	0.85	0.03	0.50	1.00	0.53	0.50	0.99	0.82	0.03	0.50	0.99
RM3117	0.74	0.50	1.00	0.85	0.03	0.51	1.00	0.72	0.50	0.99	0.82	0.03	0.50	0.99
RM295	0.52	0.50	0.99	0.85	0.03	0.50	0.99	**0.50**	**0.50**	**0.98**	**0.81**	**0.03**	**0.50**	**0.98**
RM5356	**0.50**	**0.50**	**1.00**	**0.86**	**0.03**	**0.50**	**1.00**	**0.50**	**0.50**	**0.99**	**0.82**	**0.03**	**0.50**	**0.99**
RM283	**0.50**	**0.50**	**1.00**	**0.85**	**0.03**	**0.50**	**1.00**	0.92	0.50	0.99	0.82	0.03	0.50	0.99
RM7102	**0.50**	**0.50**	**1.00**	**0.85**	**0.03**	**0.50**	**1.00**	0.52	0.50	0.99	0.82	0.03	0.50	0.99
RM190	0.50	0.50	1.00	0.85	0.03	0.50	1.00	**0.50**	**0.50**	**0.99**	**0.82**	**0.03**	**0.50**	**0.99**
RM5390	0.53	0.50	1.00	0.85	0.03	0.50	1.00	0.51	0.50	0.99	0.82	0.03	0.50	0.99

	F_2 CM-RM172							F_2 non-CM-RM172						
	Obs. F	Min F	Max F	Mean*	SE*	L95*	U95*	Obs. F	Min F	Max F	Mean*	SE*	L95*	U95*
RM527	**0.50**	**0.50**	**1.00**	**0.85**	**0.03**	**0.50**	**1.00**	**0.50**	**0.50**	**0.98**	**0.82**	**0.03**	**0.50**	**0.98**
RM23	**0.50**	**0.50**	**1.00**	**0.86**	**0.03**	**0.51**	**1.00**	0.51	0.50	0.98	0.82	0.03	0.50	0.98
RM231	0.64	0.50	1.00	0.85	0.03	0.50	1.00	0.51	0.50	0.98	0.81	0.03	0.50	0.98
RM4862	0.58	0.50	1.00	0.85	0.03	0.50	1.00	0.54	0.50	0.98	0.82	0.03	0.50	0.98
RM447	**0.50**	**0.50**	**1.00**	**0.85**	**0.03**	**0.51**	**1.00**	0.50	0.50	0.98	0.82	0.03	0.50	0.98
RM16	0.53	0.50	1.00	0.86	0.03	0.50	1.00	0.53	0.50	0.98	0.81	0.03	0.50	0.98
RM214	0.51	0.50	1.00	0.86	0.03	0.50	1.00	0.51	0.50	0.98	0.81	0.03	0.50	0.98
RM3533	**0.50**	**0.50**	**1.00**	**0.85**	**0.03**	**0.50**	**1.00**	0.50	0.50	0.98	0.81	0.03	0.50	0.98
RM211	**0.50**	**0.50**	**1.00**	**0.85**	**0.03**	**0.50**	**1.00**	**0.50**	**0.50**	**0.98**	**0.81**	**0.03**	**0.50**	**0.98**
RM296	**0.50**	**0.50**	**1.00**	**0.85**	**0.03**	**0.51**	**1.00**	0.55	0.50	0.98	0.80	0.03	0.50	0.98
RM84	0.55	0.50	1.00	0.86	0.03	0.50	1.00	0.53	0.50	0.98	0.81	0.03	0.50	0.98

RM4128	0.60	0.50	1.00	0.86	0.03	0.50	1.00	0.55	0.50	0.98	0.81	0.03	0.50	0.98
RM172	-	-	-	-	-	-	-	-	-	-	-	-	-	-
RM5	0.54	0.50	1.00	0.85	0.03	0.50	1.00	0.51	0.50	0.98	0.81	0.03	0.50	0.98
RM168	0.51	0.50	1.00	0.86	0.03	0.50	1.00	**0.50**	**0.50**	**0.98**	**0.82**	**0.03**	**0.51**	**0.98**
RM186	0.51	0.50	1.00	0.85	0.03	0.51	1.00	0.51	0.50	0.98	0.81	0.03	0.50	0.98
RM520	0.70	0.50	1.00	0.85	0.03	0.50	1.00	0.89	0.50	0.98	0.81	0.03	0.50	0.98
RM8277	0.51	0.50	1.00	0.86	0.03	0.51	1.00	0.50	0.50	0.98	0.82	0.03	0.50	0.98
RM8243	0.51	0.50	1.00	0.87	0.03	0.50	1.00	0.52	0.50	0.98	0.82	0.03	0.50	0.98
RM532	0.59	0.50	0.99	0.84	0.03	0.50	0.99	0.73	0.50	0.98	0.80	0.03	0.50	0.98
RM128	0.56	0.50	1.00	0.86	0.03	0.50	1.00	0.50	0.50	0.98	0.81	0.03	0.50	0.98
RM8206	0.52	0.50	1.00	0.85	0.03	0.50	1.00	0.56	0.50	0.98	0.81	0.03	0.50	0.98
RM340	**0.50**	**0.50**	**1.00**	**0.85**	**0.03**	**0.50**	**1.00**	0.51	0.50	0.98	0.82	0.03	0.50	0.98
RM587	0.52	0.50	1.00	0.85	0.03	0.50	1.00	0.51	0.50	0.98	0.81	0.03	0.50	0.98
RM530	0.50	0.50	1.00	0.86	0.03	0.50	1.00	0.51	0.50	0.98	0.81	0.03	0.50	0.98
RM3117	0.68	0.50	1.00	0.85	0.03	0.50	1.00	0.78	0.50	0.98	0.82	0.03	0.50	0.98
RM295	**0.50**	**0.50**	**0.99**	**0.85**	**0.03**	**0.50**	**0.99**	0.52	0.50	0.97	0.80	0.03	0.50	0.97
RM5356	**0.50**	**0.50**	**1.00**	**0.86**	**0.03**	**0.50**	**1.00**	0.51	0.50	0.98	0.81	0.03	0.50	0.98
RM283	0.52	0.50	1.00	0.86	0.03	0.51	1.00	0.50	0.50	0.98	0.82	0.03	0.50	0.98
RM7102	**0.50**	**0.50**	**1.00**	**0.85**	**0.03**	**0.51**	**1.00**	**0.50**	**0.50**	**0.98**	**0.81**	**0.03**	**0.50**	**0.98**
RM190	0.50	0.50	1.00	0.85	0.03	0.50	1.00	0.50	0.50	0.98	0.81	0.03	0.50	0.98
RM5390	**0.50**	**0.50**	**1.00**	**0.86**	**0.03**	**0.51**	**1.00**	**0.50**	**0.50**	**0.98**	**0.81**	**0.03**	**0.50**	**0.98**

	F2 WM-RM172							F2 non-WM-RM172						
	Obs. F	Min F	Max F	Mean*	SE*	L95*	U95*	Obs. F	Min F	Max F	Mean*	SE*	L95*	U95*
RM527	**0.50**	**0.50**	**1.00**	**0.85**	**0.03**	**0.50**	**1.00**	0.51	0.50	0.99	0.83	0.03	0.50	0.99
RM23	**0.50**	**0.50**	**1.00**	**0.85**	**0.03**	**0.50**	**1.00**	0.50	0.50	0.99	0.83	0.03	0.50	0.99

RM231	0.59	0.50	1.00	0.85	0.03	0.50	1.00	0.68	0.50	0.99	0.82	0.03	0.50	0.99
RM4862	0.62	0.50	1.00	0.86	0.03	0.50	1.00	0.51	0.50	0.99	0.83	0.03	0.50	0.99
RM447	**0.50**	**0.50**	**1.00**	**0.85**	**0.03**	**0.50**	**1.00**	0.54	0.50	0.99	0.83	0.03	0.50	0.99
RM16	0.52	0.50	1.00	0.86	0.03	0.50	1.00	0.55	0.50	0.99	0.82	0.03	0.50	0.99
RM214	0.51	0.50	1.00	0.85	0.03	0.50	1.00	0.51	0.50	0.99	0.83	0.03	0.50	0.99
RM3533	**0.50**	**0.50**	**1.00**	**0.85**	**0.03**	**0.50**	**1.00**	0.53	0.50	0.99	0.83	0.03	0.50	0.99
RM211	**0.50**	**0.50**	**1.00**	**0.85**	**0.03**	**0.51**	**1.00**	**0.50**	**0.50**	**0.99**	**0.84**	**0.03**	**0.50**	**0.99**
RM296	0.51	0.50	1.00	0.85	0.03	0.50	1.00	**0.50**	**0.50**	**0.99**	**0.83**	**0.03**	**0.50**	**0.99**
RM84	0.54	0.50	1.00	0.85	0.03	0.50	1.00	0.57	0.50	0.99	0.83	0.03	0.50	0.99
RM4128	0.62	0.50	1.00	0.86	0.03	0.51	1.00	0.54	0.50	0.99	0.83	0.03	0.50	0.99
RM172	-	-	-	-	-	-	-	-	-	-	-	-	-	-
RM5	0.53	0.50	1.00	0.85	0.03	0.50	1.00	0.53	0.50	0.99	0.84	0.03	0.50	0.99
RM168	0.50	0.50	1.00	0.85	0.03	0.50	1.00	0.51	0.50	0.99	0.84	0.03	0.50	0.99
RM186	**0.50**	**0.50**	**1.00**	**0.85**	**0.03**	**0.51**	**1.00**	0.52	0.50	0.99	0.83	0.03	0.50	0.99
RM520	0.78	0.50	1.00	0.84	0.03	0.51	1.00	0.64	0.50	0.99	0.83	0.03	0.50	0.99
RM8277	**0.50**	**0.50**	**1.00**	**0.85**	**0.03**	**0.50**	**1.00**	0.52	0.50	0.99	0.83	0.03	0.50	0.99
RM8243	0.51	0.50	1.00	0.85	0.03	0.50	1.00	**0.50**	**0.50**	**0.99**	**0.83**	**0.03**	**0.51**	**0.99**
RM532	0.64	0.50	0.99	0.83	0.03	0.50	0.99	0.59	0.50	0.99	0.82	0.03	0.50	0.99
RM128	0.53	0.50	1.00	0.86	0.03	0.50	1.00	0.62	0.50	0.99	0.83	0.03	0.50	0.99
RM8206	0.50	0.50	1.00	0.84	0.03	0.50	1.00	0.53	0.50	0.99	0.84	0.03	0.50	0.99
RM340	**0.50**	**0.50**	**1.00**	**0.84**	**0.03**	**0.50**	**1.00**	**0.50**	**0.50**	**0.99**	**0.84**	**0.03**	**0.50**	**0.99**
RM587	0.53	0.50	1.00	0.86	0.03	0.50	1.00	0.51	0.50	0.99	0.83	0.03	0.50	0.99
RM530	0.51	0.50	1.00	0.86	0.03	0.50	1.00	**0.50**	**0.50**	**0.99**	**0.83**	**0.03**	**0.50**	**0.99**
RM3117	0.70	0.50	1.00	0.85	0.03	0.50	1.00	0.67	0.50	0.99	0.83	0.03	0.50	0.99
RM295	**0.50**	**0.50**	**0.99**	**0.84**	**0.03**	**0.50**	**0.99**	**0.50**	**0.50**	**0.99**	**0.82**	**0.03**	**0.50**	**0.99**
RM5356	0.50	0.50	1.00	0.85	0.03	0.50	1.00	0.52	0.50	0.99	0.84	0.03	0.50	0.99

RM283	**0.50**	**0.50**	**1.00**	**0.86**	**0.03**	**0.51**	**1.00**	0.56	0.50	0.99	0.82	0.03	0.50	0.99
RM7102	**0.50**	**0.50**	**0.99**	**0.85**	**0.03**	**0.51**	**0.99**	**0.50**	**0.50**	**0.99**	**0.84**	**0.03**	**0.50**	**0.99**
RM190	0.51	0.50	1.00	0.85	0.03	0.51	1.00	0.51	0.50	0.99	0.83	0.03	0.50	0.99
RM5390	**0.50**	**0.50**	**1.00**	**0.86**	**0.03**	**0.51**	**1.00**	0.51	0.50	0.99	0.83	0.03	0.51	0.99

	F_2 CM-RM186							F_2 non-CM-RM186						
	Obs. F	Min F	Max F	Mean*	SE*	L95*	U95*	Obs. F	Min F	Max F	Mean*	SE*	L95*	U95*
RM527	**0.50**	**0.50**	**1.00**	**0.86**	**0.03**	**0.50**	**1.00**	0.51	0.50	0.98	0.82	0.03	0.50	0.98
RM23	**0.50**	**0.50**	**1.00**	**0.86**	**0.03**	**0.50**	**1.00**	0.52	0.50	0.98	0.82	0.03	0.50	0.98
RM231	0.63	0.50	1.00	0.85	0.03	0.50	1.00	0.54	0.50	0.98	0.81	0.03	0.50	0.98
RM4862	0.60	0.50	1.00	0.84	0.03	0.50	1.00	0.50	0.50	0.98	0.82	0.03	0.50	0.98
RM447	**0.50**	**0.50**	**1.00**	**0.85**	**0.03**	**0.50**	**1.00**	0.54	0.50	0.98	0.82	0.03	0.50	0.98
RM16	0.58	0.50	1.00	0.85	0.03	0.51	1.00	0.61	0.50	0.98	0.82	0.03	0.50	0.98
RM214	0.51	0.50	1.00	0.85	0.03	0.51	1.00	0.53	0.50	0.98	0.81	0.03	0.50	0.98
RM3533	**0.50**	**0.50**	**1.00**	**0.86**	**0.03**	**0.50**	**1.00**	0.53	0.50	0.98	0.80	0.03	0.50	0.98
RM211	**0.50**	**0.50**	**1.00**	**0.86**	**0.03**	**0.50**	**1.00**	0.51	0.50	0.98	0.81	0.03	0.50	0.98
RM296	**0.50**	**0.50**	**1.00**	**0.86**	**0.03**	**0.50**	**1.00**	0.53	0.50	0.98	0.81	0.03	0.50	0.98
RM84	0.56	0.50	1.00	0.86	0.03	0.50	1.00	**0.50**	**0.50**	**0.98**	**0.81**	**0.03**	**0.50**	**0.98**
RM4128	0.63	0.50	1.00	0.85	0.03	0.50	1.00	**0.50**	**0.50**	**0.98**	**0.82**	**0.03**	**0.50**	**0.98**
RM172	0.51	0.50	1.00	0.86	0.03	0.50	1.00	0.50	0.50	0.98	0.82	0.03	0.50	0.98
RM5	0.54	0.50	1.00	0.85	0.03	0.50	1.00	0.51	0.50	0.98	0.82	0.03	0.50	0.98
RM168	0.54	0.50	1.00	0.86	0.03	0.50	1.00	0.83	0.50	0.98	0.81	0.03	0.50	0.98
RM186	-	-	-	-	-	-	-	-	-	-	-	-	-	-
RM520	0.74	0.50	1.00	0.86	0.03	0.50	1.00	0.70	0.50	0.98	0.81	0.03	0.50	0.98
RM8277	0.55	0.50	1.00	0.85	0.03	0.50	1.00	0.83	0.50	0.98	0.82	0.03	0.50	0.98
RM8243	0.51	0.50	1.00	0.86	0.03	0.50	1.00	0.50	0.50	0.98	0.81	0.03	0.50	0.98

RM532	0.56	0.50	0.99	0.83	0.03	0.50	0.99	0.95	0.50	0.98	0.80	0.03	0.50	0.98
RM128	0.55	0.50	1.00	0.85	0.03	0.51	1.00	0.54	0.50	0.98	0.82	0.03	0.50	0.98
RM8206	0.51	0.50	1.00	0.86	0.03	0.50	1.00	**0.50**	**0.50**	**0.98**	**0.82**	**0.03**	**0.50**	**0.98**
RM340	**0.50**	**0.50**	**1.00**	**0.85**	**0.03**	**0.50**	**1.00**	0.59	0.50	0.98	0.81	0.03	0.50	0.98
RM587	0.51	0.50	1.00	0.86	0.03	0.50	1.00	0.57	0.50	0.98	0.82	0.03	0.50	0.98
RM530	**0.50**	**0.50**	**1.00**	**0.85**	**0.03**	**0.50**	**1.00**	0.56	0.50	0.98	0.81	0.03	0.50	0.98
RM3117	0.69	0.50	1.00	0.85	0.03	0.51	1.00	0.73	0.50	0.98	0.82	0.03	0.50	0.98
RM295	**0.50**	**0.50**	**0.99**	**0.84**	**0.03**	**0.50**	**0.99**	0.51	0.50	0.97	0.80	0.03	0.50	0.97
RM5356	**0.50**	**0.50**	**1.00**	**0.85**	**0.03**	**0.50**	**1.00**	0.53	0.50	0.98	0.82	0.03	0.50	0.98
RM283	0.52	0.50	1.00	0.86	0.03	0.50	1.00	0.50	0.50	0.98	0.81	0.03	0.50	0.98
RM7102	**0.50**	**0.50**	**1.00**	**0.85**	**0.03**	**0.50**	**1.00**	0.50	0.50	0.98	0.80	0.03	0.50	0.98
RM190	**0.50**	**0.50**	**1.00**	**0.86**	**0.03**	**0.50**	**1.00**	0.51	0.50	0.98	0.81	0.03	0.50	0.98
RM5390	**0.50**	**0.50**	**1.00**	**0.85**	**0.03**	**0.51**	**1.00**	0.54	0.50	0.98	0.81	0.03	0.50	0.98

	F_2 WM-RM186							F_2 non-WM-RM186						
	Obs. F	Min F	Max F	Mean*	SE*	L95*	U95*	Obs. F	Min F	Max F	Mean*	SE*	L95*	U95*
RM527	**0.50**	**0.50**	**1.00**	**0.85**	**0.03**	**0.50**	**1.00**	**0.50**	**0.50**	**0.99**	**0.83**	**0.03**	**0.50**	**0.99**
RM23	**0.50**	**0.50**	**1.00**	**0.85**	**0.03**	**0.50**	**1.00**	0.51	0.50	0.99	0.83	0.03	0.50	0.99
RM231	0.60	0.50	1.00	0.86	0.03	0.51	1.00	0.65	0.50	0.99	0.83	0.03	0.50	0.99
RM4862	0.56	0.50	1.00	0.85	0.03	0.50	1.00	0.61	0.50	0.99	0.83	0.03	0.50	0.99
RM447	**0.50**	**0.50**	**1.00**	**0.85**	**0.03**	**0.51**	**1.00**	0.51	0.50	0.99	0.82	0.03	0.50	0.99
RM16	0.50	0.50	1.00	0.85	0.03	0.50	1.00	0.70	0.50	0.99	0.83	0.03	0.50	0.99
RM214	0.51	0.50	1.00	0.85	0.03	0.51	1.00	0.51	0.50	0.99	0.84	0.03	0.50	0.99
RM3533	**0.50**	**0.50**	**1.00**	**0.86**	**0.03**	**0.50**	**1.00**	0.51	0.50	0.99	0.82	0.03	0.50	0.99
RM211	**0.50**	**0.50**	**1.00**	**0.85**	**0.03**	**0.51**	**1.00**	0.50	0.50	0.99	0.83	0.03	0.50	0.99
RM296	0.50	0.50	1.00	0.85	0.03	0.50	1.00	**0.50**	**0.50**	**0.99**	**0.83**	**0.03**	**0.50**	**0.99**

RM84	0.54	0.50	1.00	0.85	0.03	0.51	1.00	0.56	0.50	0.99	0.83	0.03	0.50	0.99
RM4128	0.58	0.50	1.00	0.85	0.03	0.50	1.00	0.61	0.50	0.99	0.83	0.03	0.50	0.99
RM172	**0.50**	**0.50**	**1.00**	**0.85**	**0.03**	**0.51**	**1.00**	0.55	0.50	0.99	0.83	0.03	0.50	0.99
RM5	0.51	0.50	1.00	0.85	0.03	0.51	1.00	0.59	0.50	0.99	0.82	0.03	0.50	0.99
RM168	0.52	0.50	1.00	0.85	0.03	0.50	1.00	0.93	0.50	0.99	0.83	0.03	0.50	0.99
RM186	-	-	-	-	-	-	-	-	-	-	-	-	-	-
RM520	0.74	0.50	1.00	0.86	0.03	0.50	1.00	0.73	0.50	0.99	0.84	0.03	0.50	0.99
RM8277	0.52	0.50	1.00	0.86	0.03	0.50	1.00	0.89	0.50	0.99	0.83	0.03	0.50	0.99
RM8243	0.51	0.50	1.00	0.86	0.03	0.50	1.00	0.51	0.50	0.99	0.83	0.03	0.50	0.99
RM532	0.90	0.50	0.99	0.83	0.03	0.50	0.99	0.81	0.50	0.98	0.81	0.03	0.50	0.98
RM128	0.54	0.50	1.00	0.85	0.03	0.50	1.00	0.57	0.50	0.99	0.83	0.03	0.50	0.99
RM8206	0.51	0.50	1.00	0.86	0.03	0.50	1.00	**0.50**	**0.50**	**0.99**	**0.83**	**0.03**	**0.50**	**0.99**
RM340	**0.50**	**0.50**	**1.00**	**0.84**	**0.03**	**0.50**	**1.00**	**0.50**	**0.50**	**0.99**	**0.84**	**0.03**	**0.51**	**0.99**
RM587	0.51	0.50	1.00	0.84	0.03	0.50	1.00	0.54	0.50	0.99	0.83	0.03	0.50	0.99
RM530	0.52	0.50	1.00	0.86	0.03	0.50	1.00	0.51	0.50	0.99	0.83	0.03	0.50	0.99
RM3117	0.67	0.50	1.00	0.85	0.03	0.50	1.00	0.75	0.50	0.99	0.83	0.03	0.50	0.99
RM295	0.50	0.50	0.99	0.84	0.03	0.50	0.99	0.51	0.50	0.98	0.82	0.03	0.50	0.98
RM5356	**0.50**	**0.50**	**1.00**	**0.85**	**0.03**	**0.50**	**1.00**	**0.50**	**0.50**	**0.99**	**0.83**	**0.03**	**0.50**	**0.99**
RM283	0.51	0.50	1.00	0.85	0.03	0.50	1.00	0.51	0.50	0.99	0.82	0.03	0.50	0.99
RM7102	0.51	0.50	1.00	0.85	0.03	0.50	1.00	0.51	0.50	0.99	0.82	0.03	0.50	0.99
RM190	**0.50**	**0.50**	**1.00**	**0.84**	**0.03**	**0.50**	**1.00**	0.52	0.50	0.99	0.82	0.03	0.50	0.99
RM5390	0.50	0.50	1.00	0.85	0.03	0.50	1.00	**0.50**	**0.50**	**0.99**	**0.83**	**0.03**	**0.50**	**0.99**

	F_2 CM-RM3533							F_2 non-CM-RM3533						
	Obs. F	Min F	Max F	Mean*	SE*	L95*	U95*	Obs. F	Min F	Max F	Mean*	SE*	L95*	U95*
RM527	**0.50**	**0.50**	**1.00**	**0.86**	**0.03**	**0.50**	**1.00**	**0.50**	**0.50**	**0.99**	**0.83**	**0.03**	**0.50**	**0.99**
RM23	**0.50**	**0.50**	**1.00**	**0.86**	**0.03**	**0.51**	**1.00**	**0.50**	**0.50**	**0.99**	**0.82**	**0.03**	**0.50**	**0.99**

RM231	0.62	0.50	1.00	0.85	0.03	0.50	1.00	0.60	0.50	0.99	0.83	0.03	0.50	0.99
RM4862	0.55	0.50	1.00	0.85	0.03	0.50	1.00	0.66	0.50	0.99	0.82	0.03	0.50	0.99
RM447	0.59	0.50	1.00	0.86	0.03	0.51	1.00	**1.00**	**1.00**	**1.00**	********	********	********	**0.99**
RM16	0.52	0.50	1.00	0.85	0.03	0.50	1.00	0.55	0.50	0.99	0.83	0.03	0.50	0.99
RM214	0.53	0.50	1.00	0.85	0.03	0.50	1.00	0.52	0.50	0.99	0.81	0.03	0.50	0.99
RM3533	-	-	-	-	-	-	-	-	-	-	-	-	-	-
RM211	**0.50**	**0.50**	**1.00**	**0.85**	**0.03**	**0.50**	**1.00**	0.51	0.50	0.99	0.83	0.03	0.50	0.99
RM296	0.52	0.50	1.00	0.84	0.03	0.50	1.00	0.54	0.50	0.99	0.83	0.03	0.50	0.99
RM84	0.53	0.50	1.00	0.85	0.03	0.50	1.00	0.61	0.50	0.99	0.83	0.03	0.50	0.99
RM4128	0.59	0.50	1.00	0.85	0.03	0.50	1.00	0.60	0.50	0.99	0.82	0.03	0.50	0.99
RM172	0.52	0.50	1.00	0.85	0.03	0.50	1.00	0.50	0.50	0.99	0.83	0.03	0.50	0.99
RM5	0.51	0.50	1.00	0.86	0.03	0.51	1.00	0.65	0.50	0.99	0.82	0.03	0.50	0.99
RM168	**0.50**	**0.50**	**1.00**	**0.85**	**0.03**	**0.50**	**1.00**	0.53	0.50	0.99	0.83	0.03	0.50	0.99
RM186	0.50	0.50	1.00	0.85	0.03	0.50	1.00	0.52	0.50	0.99	0.82	0.03	0.50	0.99
RM520	0.71	0.50	1.00	0.86	0.03	0.50	1.00	0.81	0.50	0.99	0.83	0.03	0.50	0.99
RM8277	0.51	0.50	1.00	0.86	0.03	0.51	1.00	0.50	0.50	0.99	0.82	0.03	0.50	0.99
RM8243	0.51	0.50	1.00	0.85	0.03	0.50	1.00	0.51	0.50	0.99	0.83	0.03	0.50	0.99
RM532	0.65	0.50	0.99	0.84	0.03	0.50	0.99	0.56	0.50	0.98	0.81	0.03	0.50	0.98
RM128	0.52	0.50	1.00	0.85	0.03	0.50	1.00	0.72	0.50	0.99	0.83	0.03	0.50	0.99
RM8206	**0.50**	**0.50**	**1.00**	**0.85**	**0.03**	**0.50**	**1.00**	0.56	0.50	0.99	0.82	0.03	0.50	0.99
RM340	0.51	0.50	1.00	0.85	0.03	0.51	1.00	0.52	0.50	0.99	0.82	0.03	0.50	0.99
RM587	0.51	0.50	1.00	0.85	0.03	0.50	1.00	0.56	0.50	0.99	0.83	0.03	0.50	0.99
RM530	**0.50**	**0.50**	**1.00**	**0.85**	**0.03**	**0.50**	**1.00**	0.51	0.50	0.99	0.82	0.03	0.50	0.99
RM3117	0.68	0.50	1.00	0.85	0.03	0.51	1.00	0.74	0.50	0.99	0.83	0.03	0.50	0.99
RM295	**0.50**	**0.50**	**0.99**	**0.84**	**0.03**	**0.50**	**0.99**	0.55	0.50	0.98	0.81	0.03	0.50	0.98
RM5356	**0.50**	**0.50**	**1.00**	**0.86**	**0.03**	**0.50**	**1.00**	**0.50**	**0.50**	**0.99**	**0.82**	**0.03**	**0.50**	**0.99**

RM283	**0.50**	**0.50**	**1.00**	**0.85**	**0.03**	**0.50**	**1.00**	0.55	0.50	0.99	0.83	0.03	0.51	0.99
RM7102	0.51	0.50	1.00	0.85	0.03	0.50	1.00	0.51	0.50	0.99	0.82	0.03	0.50	0.99
RM190	**0.50**	**0.50**	**1.00**	**0.85**	**0.03**	**0.50**	**1.00**	0.55	0.50	0.99	0.82	0.03	0.50	0.99
RM5390	0.50	0.50	1.00	0.85	0.03	0.50	1.00	0.50	0.50	0.99	0.82	0.03	0.50	0.99

	F_2 WM-RM3533							F_2 non-WM-RM3533						
	Obs. F	Min F	Max F	Mean*	SE*	L95*	U95*	Obs. F	Min F	Max F	Mean*	SE*	L95*	U95*
RM527	**0.50**	**0.50**	**1.00**	**0.85**	**0.03**	**0.50**	**1.00**	**0.50**	**0.50**	**0.99**	**0.82**	**0.03**	**0.50**	**0.99**
RM23	**0.50**	**0.50**	**1.00**	**0.86**	**0.03**	**0.51**	**1.00**	0.52	0.50	0.99	0.84	0.03	0.50	0.99
RM231	0.61	0.50	1.00	0.84	0.03	0.50	1.00	0.61	0.50	0.99	0.82	0.03	0.50	0.99
RM4862	0.59	0.50	1.00	0.86	0.03	0.51	1.00	0.54	0.50	0.99	0.84	0.03	0.50	0.99
RM447	0.56	0.50	1.00	0.86	0.03	0.51	1.00	0.97	0.50	0.99	0.83	0.03	0.50	0.99
RM16	0.53	0.50	1.00	0.85	0.03	0.50	1.00	0.52	0.50	0.99	0.83	0.03	0.50	0.99
RM214	**0.50**	**0.50**	**1.00**	**0.86**	**0.03**	**0.51**	**1.00**	0.52	0.50	0.99	0.82	0.03	0.50	0.99
RM3533	-	-	-	-	-	-	-	-	-	-	-	-	-	-
RM211	**0.50**	**0.50**	**1.00**	**0.85**	**0.03**	**0.50**	**1.00**	**0.50**	**0.50**	**0.99**	**0.83**	**0.03**	**0.50**	**0.99**
RM296	**0.50**	**0.50**	**1.00**	**0.85**	**0.03**	**0.51**	**1.00**	0.56	0.50	0.99	0.83	0.03	0.50	0.99
RM84	0.57	0.50	1.00	0.86	0.03	0.50	1.00	0.52	0.50	0.99	0.83	0.03	0.50	0.99
RM4128	0.57	0.50	1.00	0.85	0.03	0.50	1.00	0.66	0.50	0.99	0.83	0.03	0.50	0.99
RM172	0.51	0.50	1.00	0.85	0.03	0.51	1.00	0.51	0.50	0.99	0.83	0.03	0.50	0.99
RM5	0.54	0.50	1.00	0.85	0.03	0.50	1.00	0.51	0.50	0.99	0.84	0.03	0.50	0.99
RM168	0.51	0.50	1.00	0.85	0.03	0.50	1.00	**0.50**	**0.50**	**0.99**	**0.83**	**0.03**	**0.50**	**0.99**
RM186	0.50	0.50	1.00	0.85	0.03	0.50	1.00	0.51	0.50	0.99	0.84	0.03	0.50	0.99
RM520	0.74	0.50	1.00	0.85	0.03	0.50	1.00	0.72	0.50	0.99	0.84	0.03	0.50	0.99
RM8277	0.51	0.50	1.00	0.85	0.03	0.50	1.00	0.51	0.50	0.99	0.83	0.03	0.50	0.99
RM8243	0.51	0.50	1.00	0.84	0.03	0.51	1.00	0.51	0.50	0.99	0.84	0.03	0.51	0.99

RM532	0.65	0.50	0.99	0.84	0.03	0.50	0.99	0.58	0.50	0.98	0.82	0.03	0.50	0.98
RM128	0.56	0.50	1.00	0.85	0.03	0.50	1.00	0.52	0.50	0.99	0.83	0.03	0.50	0.99
RM8206	0.51	0.50	1.00	0.85	0.03	0.50	1.00	**0.50**	**0.50**	**0.99**	**0.83**	**0.03**	**0.50**	**0.99**
RM340	**0.50**	**0.50**	**1.00**	**0.85**	**0.03**	**0.50**	**1.00**	0.50	0.50	0.99	0.83	0.03	0.50	0.99
RM587	0.52	0.50	1.00	0.85	0.03	0.50	1.00	0.51	0.50	0.99	0.84	0.03	0.50	0.99
RM530	0.51	0.50	1.00	0.85	0.03	0.50	1.00	**0.50**	**0.50**	**0.99**	**0.84**	**0.03**	**0.51**	**0.99**
RM3117	0.73	0.50	1.00	0.85	0.03	0.50	1.00	0.62	0.50	0.99	0.84	0.03	0.50	0.99
RM295	**0.50**	**0.50**	**0.99**	**0.84**	**0.03**	**0.50**	**0.99**	**0.50**	**0.50**	**0.98**	**0.81**	**0.03**	**0.50**	**0.98**
RM5356	**0.50**	**0.50**	**1.00**	**0.86**	**0.03**	**0.50**	**1.00**	0.51	0.50	0.99	0.83	0.03	0.50	0.99
RM283	0.52	0.50	1.00	0.85	0.03	0.51	1.00	**0.50**	**0.50**	**0.99**	**0.84**	**0.03**	**0.50**	**0.99**
RM7102	**0.50**	**0.50**	**0.99**	**0.84**	**0.03**	**0.50**	**0.99**	**0.50**	**0.50**	**0.99**	**0.83**	**0.03**	**0.50**	**0.99**
RM190	**0.50**	**0.50**	**1.00**	**0.85**	**0.03**	**0.51**	**1.00**	**0.50**	**0.50**	**0.99**	**0.82**	**0.03**	**0.50**	**0.99**
RM5390	**0.50**	**0.50**	**1.00**	**0.85**	**0.03**	**0.50**	**1.00**	0.52	0.50	0.99	0.83	0.03	0.50	0.99

	F_2 Ideal-1							F_2 Ideal-2						
	Obs. F	Min F	Max F	Mean*	SE*	L95*	U95*	Obs. F	Min F	Max F	Mean*	SE*	L95*	U95*
RM527	**0.50**	**0.50**	**1.00**	**0.89**	**0.02**	**0.51**	**1.00**	**0.50**	**0.50**	**1.00**	**0.88**	**0.02**	**0.51**	**1.00**
RM23	**0.50**	**0.50**	**1.00**	**0.88**	**0.03**	**0.50**	**1.00**	**0.50**	**0.50**	**1.00**	**0.88**	**0.02**	**0.51**	**1.00**
RM231	0.61	0.50	1.00	0.89	0.02	0.50	1.00	0.62	0.50	1.00	0.87	0.03	0.50	1.00
RM4862	0.57	0.50	1.00	0.88	0.03	0.50	1.00	0.57	0.50	1.00	0.88	0.03	0.50	1.00
RM447	**0.50**	**0.50**	**1.00**	**0.90**	**0.02**	**0.51**	**1.00**	**0.50**	**0.50**	**1.00**	**0.88**	**0.03**	**0.51**	**1.00**
RM16	0.53	0.50	1.00	0.90	0.02	0.50	1.00	0.54	0.50	1.00	0.88	0.03	0.51	1.00
RM214	0.51	0.50	1.00	0.89	0.03	0.51	1.00	0.51	0.50	1.00	0.89	0.02	0.51	1.00
RM3533	**0.50**	**0.50**	**1.00**	**0.89**	**0.02**	**0.50**	**1.00**	**0.50**	**0.50**	**1.00**	**0.88**	**0.03**	**0.51**	**1.00**
RM211	**0.50**	**0.50**	**1.00**	**0.90**	**0.02**	**0.51**	**1.00**	**0.50**	**0.50**	**1.00**	**0.87**	**0.03**	**0.50**	**1.00**
RM296	**0.50**	**0.50**	**1.00**	**0.89**	**0.02**	**0.51**	**1.00**	**0.50**	**0.50**	**1.00**	**0.87**	**0.03**	**0.50**	**1.00**

	F3 GE							F3 non-GE						
	Obs. F	Min F	Max F	Mean*	SE*	L95*	U95*	Obs. F	Min F	Max F	Mean*	SE*	L95*	U95*
RM84	0.55	0.50	1.00	0.88	0.03	0.50	1.00	0.55	0.50	1.00	0.87	0.03	0.50	1.00
RM4128	0.59	0.50	1.00	0.89	0.03	0.51	1.00	0.60	0.50	1.00	0.87	0.03	0.51	1.00
RM172	**0.51**	**0.50**	**1.00**	**0.90**	**0.02**	**0.51**	**1.00**	**0.51**	**0.50**	**1.00**	**0.88**	**0.03**	**0.51**	**1.00**
RM5	0.53	0.50	1.00	0.89	0.03	0.51	1.00	0.53	0.50	1.00	0.87	0.03	0.51	1.00
RM168	**0.51**	**0.50**	**1.00**	**0.89**	**0.02**	**0.51**	**1.00**	0.51	0.50	1.00	0.87	0.03	0.51	1.00
RM186	0.51	0.50	1.00	0.90	0.02	0.50	1.00	0.51	0.50	1.00	0.87	0.03	0.51	1.00
RM520	0.74	0.50	1.00	0.89	0.03	0.50	1.00	0.72	0.50	1.00	0.88	0.02	0.51	1.00
RM8277	0.51	0.50	1.00	0.88	0.03	0.50	1.00	0.51	0.50	1.00	0.88	0.02	0.51	1.00
RM8243	0.51	0.50	1.00	0.90	0.02	0.51	1.00	0.51	0.50	1.00	0.88	0.03	0.50	1.00
RM532	0.63	0.50	1.00	0.88	0.03	0.51	1.00	0.61	0.50	1.00	0.86	0.03	0.50	1.00
RM128	0.55	0.50	1.00	0.89	0.03	0.50	1.00	0.55	0.50	1.00	0.87	0.03	0.51	1.00
RM8206	0.51	0.50	1.00	0.88	0.03	0.51	1.00	0.51	0.50	1.00	0.87	0.03	0.50	1.00
RM340	**0.50**	**0.50**	**1.00**	**0.89**	**0.02**	**0.51**	**1.00**	**0.50**	**0.50**	**1.00**	**0.87**	**0.03**	**0.50**	**1.00**
RM587	0.52	0.50	1.00	0.89	0.03	0.51	1.00	0.52	0.50	1.00	0.88	0.03	0.51	1.00
RM530	**0.50**	**0.50**	**1.00**	**0.89**	**0.03**	**0.51**	**1.00**	**0.50**	**0.50**	**1.00**	**0.87**	**0.03**	**0.50**	**1.00**
RM3117	0.53	0.50	1.00	0.90	0.02	0.51	1.00	0.54	0.50	1.00	0.88	0.03	0.50	1.00
RM295	0.58	0.50	1.00	0.89	0.02	0.51	1.00	0.59	0.50	1.00	0.88	0.03	0.51	1.00
RM5356	**0.50**	**0.50**	**1.00**	**0.89**	**0.02**	**0.50**	**1.00**	**0.50**	**0.50**	**1.00**	**0.87**	**0.03**	**0.51**	**1.00**
RM283	**0.50**	**0.50**	**1.00**	**0.89**	**0.02**	**0.51**	**1.00**	**0.50**	**0.50**	**1.00**	**0.87**	**0.03**	**0.51**	**1.00**
RM7102	0.50	0.50	1.00	0.88	0.03	0.50	1.00	**0.51**	**0.50**	**1.00**	**0.87**	**0.03**	**0.51**	**1.00**
RM190	**0.50**	**0.50**	**1.00**	**0.89**	**0.02**	**0.51**	**1.00**	**0.50**	**0.50**	**1.00**	**0.87**	**0.03**	**0.51**	**1.00**
RM5390	**0.50**	**0.50**	**1.00**	**0.89**	**0.02**	**0.51**	**1.00**	**0.50**	**0.50**	**1.00**	**0.88**	**0.02**	**0.51**	**1.00**

RM527	0.51	0.50	1.00	0.87	0.03	0.51	1.00	0.51	0.50	0.99	0.84	0.03	0.50	0.99
RM23	**0.50**	**0.50**	**1.00**	**0.87**	**0.03**	**0.51**	**1.00**	0.51	0.50	0.99	0.85	0.03	0.50	0.99
RM231	0.51	0.50	1.00	0.86	0.03	0.51	1.00	0.51	0.50	0.99	0.85	0.03	0.50	0.99
RM4862	0.51	0.50	1.00	0.87	0.02	0.51	1.00	0.52	0.50	0.99	0.84	0.03	0.50	0.99
RM447	**0.50**	**0.50**	**1.00**	**0.86**	**0.03**	**0.50**	**1.00**	**0.50**	**0.50**	**0.99**	**0.84**	**0.03**	**0.50**	**0.99**
RM16	**0.50**	**0.50**	**1.00**	**0.86**	**0.03**	**0.50**	**1.00**	0.51	0.50	0.99	0.85	0.03	0.50	0.99
RM214	0.52	0.50	1.00	0.85	0.03	0.50	1.00	0.51	0.50	0.99	0.85	0.03	0.50	0.99
RM3533	**0.50**	**0.50**	**1.00**	**0.85**	**0.03**	**0.50**	**1.00**	0.51	0.50	0.99	0.84	0.03	0.50	0.99
RM211	**0.50**	**0.50**	**1.00**	**0.86**	**0.03**	**0.50**	**1.00**	0.51	0.50	0.99	0.84	0.03	0.50	0.99
RM296	0.51	0.50	1.00	0.86	0.03	0.51	1.00	0.53	0.50	0.99	0.84	0.03	0.51	0.99
RM84	0.57	0.50	1.00	0.86	0.03	0.51	1.00	**0.50**	**0.50**	**0.99**	**0.85**	**0.03**	**0.50**	**0.99**
RM4128	0.54	0.50	1.00	0.86	0.03	0.50	1.00	0.61	0.50	0.99	0.85	0.03	0.50	0.99
RM172	**0.50**	**0.50**	**1.00**	**0.86**	**0.03**	**0.50**	**1.00**	**0.50**	**0.50**	**0.99**	**0.85**	**0.03**	**0.50**	**0.99**
RM5	0.57	0.50	1.00	0.87	0.03	0.50	1.00	0.52	0.50	0.99	0.85	0.03	0.51	0.99
RM168	0.53	0.50	1.00	0.86	0.03	0.50	1.00	0.51	0.50	0.99	0.84	0.03	0.50	0.99
RM186	**0.50**	**0.50**	**1.00**	**0.86**	**0.03**	**0.50**	**1.00**	**0.50**	**0.50**	**0.99**	**0.85**	**0.03**	**0.50**	**0.99**
RM520	0.55	0.50	1.00	0.86	0.03	0.51	1.00	0.53	0.50	0.99	0.84	0.03	0.51	0.99
RM8277	**0.50**	**0.50**	**1.00**	**0.86**	**0.03**	**0.50**	**1.00**	0.51	0.50	0.99	0.85	0.03	0.50	0.99
RM8243	0.53	0.50	1.00	0.86	0.03	0.50	1.00	**0.50**	**0.50**	**0.99**	**0.85**	**0.03**	**0.50**	**0.99**
RM532	0.51	0.50	1.00	0.87	0.03	0.50	1.00	0.51	0.50	0.99	0.86	0.03	0.50	0.99
RM128	0.51	0.50	1.00	0.87	0.03	0.50	1.00	**0.50**	**0.50**	**0.99**	**0.85**	**0.03**	**0.50**	**0.99**
RM8206	0.53	0.50	1.00	0.85	0.03	0.50	1.00	0.52	0.50	0.99	0.85	0.03	0.50	0.99
RM340	**0.50**	**0.50**	**1.00**	**0.86**	**0.03**	**0.50**	**1.00**	0.54	0.50	0.99	0.84	0.03	0.50	0.99
RM587	0.52	0.50	1.00	0.85	0.03	0.51	1.00	0.51	0.50	0.99	0.85	0.03	0.50	0.99
RM530	0.52	0.50	1.00	0.86	0.03	0.51	1.00	0.54	0.50	0.99	0.84	0.03	0.50	0.99
RM3117	0.82	0.50	1.00	0.87	0.03	0.50	1.00	0.83	0.50	0.99	0.84	0.03	0.51	0.99

RM295	0.50	0.50	1.00	0.86	0.03	0.50	1.00	0.54	0.50	0.99	0.85	0.03	0.50	0.99
RM5356	0.50	0.50	1.00	0.86	0.03	0.50	1.00	0.50	0.50	0.99	0.84	0.03	0.50	0.99
RM283	0.51	0.50	1.00	0.86	0.03	0.50	1.00	0.77	0.50	0.99	0.85	0.03	0.50	0.99
RM7102	**0.50**	**0.50**	**1.00**	**0.85**	**0.03**	**0.51**	**1.00**	0.51	0.50	0.99	0.85	0.03	0.50	0.99
RM190	0.51	0.50	1.00	0.86	0.03	0.51	1.00	**0.50**	**0.50**	**0.99**	**0.85**	**0.03**	**0.51**	**0.99**
RM5390	0.51	0.50	1.00	0.86	0.03	0.50	1.00	0.51	0.50	0.99	0.85	0.03	0.51	0.99

	F_3 CM-RM172							F_3 non-CM-RM172						
	Obs. F	Min F	Max F	Mean*	SE*	L95*	U95*	Obs. F	Min F	Max F	Mean*	SE*	L95*	U95*
RM527	0.51	0.50	1.00	0.87	0.03	0.51	1.00	0.53	0.50	1.00	0.85	0.03	0.51	1.00
RM23	0.53	0.50	1.00	0.87	0.03	0.51	1.00	0.50	0.50	1.00	0.85	0.03	0.50	1.00
RM231	0.55	0.50	1.00	0.86	0.03	0.51	1.00	**0.50**	**0.50**	**1.00**	**0.84**	**0.03**	**0.50**	**1.00**
RM4862	**0.50**	**0.50**	**1.00**	**0.87**	**0.02**	**0.51**	**1.00**	**0.50**	**0.50**	**1.00**	**0.85**	**0.03**	**0.50**	**1.00**
RM447	**0.50**	**0.50**	**1.00**	**0.86**	**0.03**	**0.50**	**1.00**	**0.50**	**0.50**	**1.00**	**0.85**	**0.03**	**0.50**	**1.00**
RM16	**0.50**	**0.50**	**1.00**	**0.86**	**0.03**	**0.50**	**1.00**	0.50	0.50	1.00	0.84	0.03	0.50	1.00
RM214	**0.50**	**0.50**	**1.00**	**0.85**	**0.03**	**0.50**	**1.00**	**0.50**	**0.50**	**1.00**	**0.85**	**0.03**	**0.50**	**1.00**
RM3533	**0.50**	**0.50**	**1.00**	**0.85**	**0.03**	**0.50**	**1.00**	0.50	0.50	1.00	0.85	0.03	0.50	1.00
RM211	**0.50**	**0.50**	**1.00**	**0.86**	**0.03**	**0.50**	**1.00**	**0.50**	**0.50**	**1.00**	**0.85**	**0.03**	**0.50**	**1.00**
RM296	**0.50**	**0.50**	**1.00**	**0.86**	**0.03**	**0.51**	**1.00**	0.55	0.50	1.00	0.84	0.03	0.50	1.00
RM84	0.54	0.50	1.00	0.86	0.03	0.51	1.00	0.50	0.50	1.00	0.85	0.03	0.50	1.00
RM4128	**0.50**	**0.50**	**1.00**	**0.86**	**0.03**	**0.50**	**1.00**	0.51	0.50	1.00	0.85	0.03	0.51	1.00
RM172	-	-	-	-	-	-	-	-	-	-	-	-	-	-
RM5	0.54	0.50	1.00	0.87	0.03	0.50	1.00	0.52	0.50	1.00	0.86	0.03	0.50	1.00
RM168	**0.50**	**0.50**	**1.00**	**0.86**	**0.03**	**0.50**	**1.00**	0.51	0.50	1.00	0.85	0.03	0.50	1.00
RM186	**0.50**	**0.50**	**1.00**	**0.85**	**0.03**	**0.50**	**1.00**	**0.50**	**0.50**	**1.00**	**0.85**	**0.03**	**0.51**	**1.00**
RM520	0.72	0.50	1.00	0.86	0.03	0.51	1.00	0.83	0.50	1.00	0.85	0.03	0.50	1.00

RM8277	**0.50**	**0.50**	**1.00**	**0.86**	**0.03**	**0.50**	**1.00**	**0.50**	**0.50**	**1.00**	**0.85**	**0.03**	**0.50**	**1.00**
RM8243	**0.50**	**0.50**	**1.00**	**0.86**	**0.03**	**0.50**	**1.00**	**0.50**	**0.50**	**1.00**	**0.84**	**0.03**	**0.51**	**1.00**
RM532	0.52	0.50	1.00	0.87	0.03	0.50	1.00	0.51	0.50	0.99	0.85	0.03	0.50	0.99
RM128	0.60	0.50	1.00	0.87	0.03	0.50	1.00	0.54	0.50	1.00	0.85	0.03	0.50	1.00
RM8206	0.52	0.50	1.00	0.85	0.03	0.50	1.00	0.50	0.50	1.00	0.86	0.03	0.50	1.00
RM340	0.51	0.50	1.00	0.86	0.03	0.50	1.00	**0.50**	**0.50**	**1.00**	**0.85**	**0.03**	**0.50**	**1.00**
RM587	0.51	0.50	1.00	0.85	0.03	0.51	1.00	0.54	0.50	1.00	0.85	0.03	0.50	1.00
RM530	**0.50**	**0.50**	**1.00**	**0.87**	**0.03**	**0.50**	**1.00**	0.50	0.50	1.00	0.85	0.03	0.50	1.00
RM3117	0.67	0.50	1.00	0.86	0.03	0.50	1.00	0.72	0.50	1.00	0.85	0.03	0.50	1.00
RM295	**0.50**	**0.50**	**1.00**	**0.86**	**0.03**	**0.50**	**1.00**	**0.50**	**0.50**	**1.00**	**0.85**	**0.03**	**0.51**	**1.00**
RM5356	**0.50**	**0.50**	**1.00**	**0.85**	**0.03**	**0.50**	**1.00**	0.50	0.50	1.00	0.86	0.03	0.50	1.00
RM283	0.54	0.50	1.00	0.85	0.03	0.51	1.00	**0.50**	**0.50**	**1.00**	**0.84**	**0.03**	**0.50**	**1.00**
RM7102	**0.50**	**0.50**	**1.00**	**0.86**	**0.03**	**0.51**	**1.00**	0.51	0.50	1.00	0.86	0.03	0.50	1.00
RM190	0.50	0.50	1.00	0.86	0.03	0.50	1.00	0.53	0.50	1.00	0.85	0.03	0.50	1.00
RM5390	0.51	0.50	1.00	0.85	0.03	0.50	1.00	**0.50**	**0.50**	**1.00**	**0.85**	**0.03**	**0.51**	**1.00**

	F_3 WM-RM172							F_3 non-WM-RM172						
	Obs. F	Min F	Max F	Mean*	SE*	L95*	U95*	Obs. F	Min F	Max F	Mean*	SE*	L95*	U95*
RM527	0.53	0.50	1.00	0.86	0.03	0.50	1.00	0.51	0.50	1.00	0.85	0.03	0.50	1.00
RM23	0.51	0.50	1.00	0.86	0.03	0.50	1.00	0.53	0.50	1.00	0.85	0.03	0.50	1.00
RM231	**0.50**	**0.50**	**1.00**	**0.85**	**0.03**	**0.50**	**1.00**	0.59	0.50	1.00	0.84	0.03	0.50	1.00
RM4862	**0.50**	**0.50**	**1.00**	**0.86**	**0.03**	**0.50**	**1.00**	0.51	0.50	1.00	0.85	0.03	0.50	1.00
RM447	**0.50**	**0.50**	**1.00**	**0.86**	**0.03**	**0.50**	**1.00**	**0.50**	**0.50**	**1.00**	**0.85**	**0.03**	**0.50**	**1.00**
RM16	**0.50**	**0.50**	**1.00**	**0.85**	**0.03**	**0.50**	**1.00**	**0.50**	**0.50**	**1.00**	**0.84**	**0.03**	**0.50**	**1.00**
RM214	**0.50**	**0.50**	**1.00**	**0.86**	**0.03**	**0.50**	**1.00**	**0.50**	**0.50**	**1.00**	**0.85**	**0.03**	**0.50**	**1.00**
RM3533	**0.50**	**0.50**	**1.00**	**0.86**	**0.03**	**0.50**	**1.00**	**0.50**	**0.50**	**1.00**	**0.85**	**0.03**	**0.50**	**1.00**

RM211	**0.50**	**0.50**	**1.00**	**0.86**	**0.03**	**0.51**	**1.00**	**0.50**	**0.50**	**1.00**	**0.85**	**0.03**	**0.50**	**1.00**
RM296	0.53	0.50	1.00	0.86	0.03	0.50	1.00	0.50	0.50	1.00	0.85	0.03	0.50	1.00
RM84	0.51	0.50	1.00	0.86	0.03	0.50	1.00	0.54	0.50	1.00	0.86	0.03	0.51	1.00
RM4128	0.51	0.50	1.00	0.85	0.03	0.50	1.00	**0.50**	**0.50**	**1.00**	**0.85**	**0.03**	**0.50**	**1.00**
RM172	-	-	-	-	-	-	-	-	-	-	-	-	-	-
RM5	0.53	0.50	1.00	0.87	0.03	0.50	1.00	0.55	0.50	1.00	0.85	0.03	0.50	1.00
RM168	0.52	0.50	1.00	0.86	0.03	0.50	1.00	**0.50**	**0.50**	**1.00**	**0.86**	**0.03**	**0.51**	**1.00**
RM186	**0.50**	**0.50**	**1.00**	**0.86**	**0.03**	**0.50**	**1.00**	**0.50**	**0.50**	**1.00**	**0.86**	**0.03**	**0.51**	**1.00**
RM520	0.84	0.50	1.00	0.87	0.03	0.50	1.00	0.65	0.50	1.00	0.85	0.03	0.50	1.00
RM8277	**0.50**	**0.50**	**1.00**	**0.86**	**0.03**	**0.50**	**1.00**	**0.50**	**0.50**	**1.00**	**0.85**	**0.03**	**0.50**	**1.00**
RM8243	**0.50**	**0.50**	**1.00**	**0.86**	**0.03**	**0.50**	**1.00**	0.50	0.50	1.00	0.85	0.03	0.50	1.00
RM532	**0.51**	**0.50**	**1.00**	**0.87**	**0.03**	**0.51**	**1.00**	0.53	0.50	1.00	0.86	0.03	0.50	1.00
RM128	0.56	0.50	1.00	0.86	0.03	0.50	1.00	0.61	0.50	1.00	0.85	0.03	0.50	1.00
RM8206	**0.50**	**0.50**	**1.00**	**0.86**	**0.03**	**0.50**	**1.00**	0.52	0.50	1.00	0.86	0.03	0.51	1.00
RM340	0.51	0.50	1.00	0.86	0.03	0.51	1.00	**0.50**	**0.50**	**1.00**	**0.86**	**0.03**	**0.50**	**1.00**
RM587	0.54	0.50	1.00	0.86	0.03	0.50	1.00	**0.50**	**0.50**	**1.00**	**0.85**	**0.03**	**0.50**	**1.00**
RM530	**0.50**	**0.50**	**1.00**	**0.86**	**0.03**	**0.50**	**1.00**	**0.50**	**0.50**	**1.00**	**0.86**	**0.03**	**0.51**	**1.00**
RM3117	0.72	0.50	1.00	0.85	0.03	0.50	1.00	0.65	0.50	1.00	0.85	0.03	0.51	1.00
RM295	**0.50**	**0.50**	**1.00**	**0.85**	**0.03**	**0.50**	**1.00**	**0.50**	**0.50**	**1.00**	**0.84**	**0.03**	**0.50**	**1.00**
RM5356	0.50	0.50	1.00	0.85	0.03	0.50	1.00	0.50	0.50	1.00	0.85	0.03	0.50	1.00
RM283	0.51	0.50	1.00	0.86	0.03	0.50	1.00	0.57	0.50	1.00	0.86	0.03	0.50	1.00
RM7102	**0.51**	**0.50**	**1.00**	**0.86**	**0.03**	**0.51**	**1.00**	**0.50**	**0.50**	**1.00**	**0.85**	**0.03**	**0.51**	**1.00**
RM190	0.53	0.50	1.00	0.85	0.03	0.50	1.00	**0.50**	**0.50**	**1.00**	**0.86**	**0.03**	**0.50**	**1.00**
RM5390	**0.50**	**0.50**	**1.00**	**0.86**	**0.03**	**0.50**	**1.00**	0.51	0.50	1.00	0.86	0.03	0.50	1.00

F_3 CM-RM186

F_3 non-CM-RM186

	Obs. F	Min F	Max F	Mean*	SE*	L95*	U95*	Obs. F	Min F	Max F	Mean*	SE*	L95*	U95*
RM527	0.50	0.50	1.00	0.86	0.03	0.50	1.00	0.56	0.50	1.00	0.85	0.03	0.51	1.00
RM23	0.51	0.50	1.00	0.85	0.03	0.50	1.00	0.53	0.50	1.00	0.86	0.03	0.51	1.00
RM231	0.52	0.50	1.00	0.86	0.03	0.50	1.00	0.52	0.50	1.00	0.85	0.03	0.51	1.00
RM4862	**0.50**	**0.50**	**1.00**	**0.86**	**0.03**	**0.51**	**1.00**	0.51	0.50	1.00	0.86	0.03	0.50	1.00
RM447	**0.50**	**0.50**	**1.00**	**0.86**	**0.03**	**0.50**	**1.00**	0.52	0.50	1.00	0.85	0.03	0.50	1.00
RM16	0.53	0.50	1.00	0.86	0.03	0.50	1.00	0.53	0.50	1.00	0.85	0.03	0.50	1.00
RM214	**0.50**	**0.50**	**1.00**	**0.86**	**0.03**	**0.51**	**1.00**	0.52	0.50	1.00	0.84	0.03	0.50	1.00
RM3533	**0.50**	**0.50**	**1.00**	**0.86**	**0.03**	**0.50**	**1.00**	**0.50**	**0.50**	**1.00**	**0.84**	**0.03**	**0.50**	**1.00**
RM211	**0.50**	**0.50**	**1.00**	**0.85**	**0.03**	**0.50**	**1.00**	0.50	0.50	1.00	0.85	0.03	0.50	1.00
RM296	0.51	0.50	1.00	0.87	0.03	0.51	1.00	0.52	0.50	1.00	0.85	0.03	0.51	1.00
RM84	0.52	0.50	1.00	0.87	0.03	0.51	1.00	0.52	0.50	1.00	0.84	0.03	0.50	1.00
RM4128	**0.50**	**0.50**	**1.00**	**0.86**	**0.03**	**0.50**	**1.00**	0.55	0.50	1.00	0.85	0.03	0.50	1.00
RM172	**0.50**	**0.50**	**1.00**	**0.86**	**0.03**	**0.50**	**1.00**	**0.50**	**0.50**	**1.00**	**0.85**	**0.03**	**0.50**	**1.00**
RM5	0.52	0.50	1.00	0.85	0.03	0.50	1.00	0.56	0.50	1.00	0.86	0.03	0.50	1.00
RM168	0.63	0.50	1.00	0.87	0.03	0.50	1.00	0.67	0.50	1.00	0.85	0.03	0.50	1.00
RM186	-	-	-	-	-	-	-	-	-	-	-	-	-	-
RM520	0.83	0.50	1.00	0.87	0.03	0.51	1.00	0.64	0.50	1.00	0.84	0.03	0.50	1.00
RM8277	0.66	0.50	1.00	0.87	0.03	0.50	1.00	0.81	0.50	1.00	0.85	0.03	0.50	1.00
RM8243	0.52	0.50	1.00	0.86	0.03	0.50	1.00	0.50	0.50	1.00	0.85	0.03	0.50	1.00
RM532	0.51	0.50	1.00	0.86	0.03	0.50	1.00	0.73	0.50	1.00	0.85	0.03	0.50	1.00
RM128	0.55	0.50	1.00	0.87	0.03	0.51	1.00	0.63	0.50	1.00	0.86	0.03	0.50	1.00
RM8206	**0.50**	**0.50**	**1.00**	**0.86**	**0.03**	**0.51**	**1.00**	0.52	0.50	1.00	0.86	0.03	0.50	1.00
RM340	**0.50**	**0.50**	**1.00**	**0.85**	**0.03**	**0.50**	**1.00**	0.53	0.50	1.00	0.84	0.03	0.50	1.00
RM587	0.51	0.50	1.00	0.86	0.03	0.50	1.00	0.52	0.50	1.00	0.85	0.03	0.50	1.00
RM530	**0.50**	**0.50**	**1.00**	**0.86**	**0.03**	**0.51**	**1.00**	**0.50**	**0.50**	**1.00**	**0.85**	**0.03**	**0.51**	**1.00**

RM3117	0.69	0.50	1.00	0.86	0.03	0.50	1.00	0.69	0.50	1.00	0.86	0.03	0.50	1.00
RM295	**0.50**	**0.50**	**1.00**	**0.87**	**0.03**	**0.50**	**1.00**	0.51	0.50	1.00	0.85	0.03	0.50	1.00
RM5356	0.50	0.50	1.00	0.86	0.03	0.50	1.00	0.51	0.50	1.00	0.85	0.03	0.50	1.00
RM283	0.51	0.50	1.00	0.85	0.03	0.50	1.00	0.55	0.50	1.00	0.84	0.03	0.50	1.00
RM7102	**0.50**	**0.50**	**1.00**	**0.87**	**0.03**	**0.50**	**1.00**	**0.50**	**0.50**	**1.00**	**0.84**	**0.03**	**0.50**	**1.00**
RM190	0.51	0.50	1.00	0.85	0.03	0.50	1.00	0.52	0.50	1.00	0.85	0.03	0.51	1.00
RM5390	0.50	0.50	1.00	0.86	0.03	0.50	1.00	0.50	0.50	1.00	0.85	0.03	0.50	1.00

	F_3 WM-RM186							F_3 non-WM-RM186						
	Obs. F	Min F	Max F	Mean*	SE*	L95*	U95*	Obs. F	Min F	Max F	Mean*	SE*	L95*	U95*
RM527	0.54	0.50	1.00	0.85	0.03	0.50	1.00	**0.50**	**0.50**	**1.00**	**0.85**	**0.03**	**0.50**	**1.00**
RM23	0.51	0.50	1.00	0.86	0.03	0.50	1.00	0.52	0.50	1.00	0.85	0.03	0.50	1.00
RM231	0.52	0.50	1.00	0.86	0.03	0.50	1.00	0.54	0.50	1.00	0.85	0.03	0.50	1.00
RM4862	**0.50**	**0.50**	**1.00**	**0.86**	**0.03**	**0.50**	**1.00**	**0.50**	**0.50**	**1.00**	**0.85**	**0.03**	**0.50**	**1.00**
RM447	**0.50**	**0.50**	**1.00**	**0.87**	**0.03**	**0.51**	**1.00**	**0.50**	**0.50**	**1.00**	**0.84**	**0.03**	**0.50**	**1.00**
RM16	**0.50**	**0.50**	**1.00**	**0.86**	**0.03**	**0.51**	**1.00**	0.55	0.50	1.00	0.85	0.03	0.50	1.00
RM214	0.50	0.50	1.00	0.87	0.03	0.50	1.00	**0.50**	**0.50**	**1.00**	**0.85**	**0.03**	**0.50**	**1.00**
RM3533	**0.50**	**0.50**	**1.00**	**0.86**	**0.03**	**0.51**	**1.00**	0.51	0.50	1.00	0.84	0.03	0.50	1.00
RM211	**0.50**	**0.50**	**1.00**	**0.85**	**0.03**	**0.50**	**1.00**	**0.50**	**0.50**	**1.00**	**0.85**	**0.03**	**0.50**	**1.00**
RM296	0.52	0.50	1.00	0.87	0.03	0.50	1.00	0.51	0.50	1.00	0.85	0.03	0.50	1.00
RM84	0.52	0.50	1.00	0.86	0.03	0.50	1.00	0.53	0.50	1.00	0.85	0.03	0.51	1.00
RM4128	0.52	0.50	1.00	0.86	0.03	0.50	1.00	0.51	0.50	1.00	0.85	0.03	0.50	1.00
RM172	**0.50**	**0.50**	**1.00**	**0.86**	**0.03**	**0.50**	**1.00**	**0.50**	**0.50**	**1.00**	**0.85**	**0.03**	**0.50**	**1.00**
RM5	0.52	0.50	1.00	0.86	0.03	0.50	1.00	0.56	0.50	1.00	0.84	0.03	0.50	1.00
RM168	0.58	0.50	1.00	0.86	0.03	0.50	1.00	0.95	0.50	1.00	0.85	0.03	0.50	1.00
RM186	-	-	-	-	-	-	-	-	-	-	-	-	-	-

RM520	0.71	0.50	1.00	0.86	0.03	0.50	1.00	0.83	0.50	1.00	0.86	0.03	0.50	1.00
RM8277	0.61	0.50	1.00	0.86	0.03	0.50	1.00	0.96	0.50	1.00	0.85	0.03	0.50	1.00
RM8243	0.50	0.50	1.00	0.85	0.03	0.50	1.00	0.52	0.50	1.00	0.85	0.03	0.50	1.00
RM532	0.75	0.50	1.00	0.86	0.03	0.51	1.00	0.78	0.50	1.00	0.86	0.03	0.50	1.00
RM128	0.58	0.50	1.00	0.86	0.03	0.50	1.00	0.57	0.50	1.00	0.85	0.03	0.50	1.00
RM8206	0.50	0.50	1.00	0.86	0.03	0.50	1.00	0.51	0.50	1.00	0.85	0.03	0.50	1.00
RM340	**0.50**	**0.50**	**1.00**	**0.86**	**0.03**	**0.50**	**1.00**	**0.50**	**0.50**	**1.00**	**0.86**	**0.03**	**0.51**	**1.00**
RM587	0.51	0.50	1.00	0.87	0.03	0.50	1.00	0.53	0.50	1.00	0.85	0.03	0.50	1.00
RM530	**0.50**	**0.50**	**1.00**	**0.87**	**0.03**	**0.50**	**1.00**	**0.50**	**0.50**	**1.00**	**0.85**	**0.03**	**0.51**	**1.00**
RM3117	0.69	0.50	1.00	0.86	0.03	0.50	1.00	0.69	0.50	1.00	0.85	0.03	0.50	1.00
RM295	0.52	0.50	1.00	0.86	0.03	0.51	1.00	0.54	0.50	1.00	0.86	0.03	0.51	1.00
RM5356	**0.50**	**0.50**	**1.00**	**0.85**	**0.03**	**0.50**	**1.00**	**0.50**	**0.50**	**1.00**	**0.85**	**0.03**	**0.50**	**1.00**
RM283	0.54	0.50	1.00	0.86	0.03	0.50	1.00	0.51	0.50	1.00	0.84	0.03	0.50	1.00
RM7102	**0.50**	**0.50**	**1.00**	**0.86**	**0.03**	**0.50**	**1.00**	**0.50**	**0.50**	**1.00**	**0.84**	**0.03**	**0.50**	**1.00**
RM190	0.51	0.50	1.00	0.86	0.03	0.50	1.00	0.51	0.50	1.00	0.84	0.03	0.50	1.00
RM5390	**0.50**	**0.50**	**1.00**	**0.85**	**0.03**	**0.50**	**1.00**	**0.50**	**0.50**	**1.00**	**0.85**	**0.03**	**0.50**	**1.00**

	F_3 CM-RM3533							F_3 non-CM-RM3533						
	Obs. F	Min F	Max F	Mean*	SE*	L95*	U95*	Obs. F	Min F	Max F	Mean*	SE*	L95*	U95*
RM527	0.52	0.50	1.00	0.86	0.03	0.50	1.00	0.52	0.50	1.00	0.85	0.03	0.50	1.00
RM23	0.56	0.50	1.00	0.85	0.03	0.50	1.00	**0.50**	**0.50**	**1.00**	**0.86**	**0.03**	**0.51**	**1.00**
RM231	0.52	0.50	1.00	0.86	0.03	0.50	1.00	0.54	0.50	1.00	0.85	0.03	0.50	1.00
RM4862	**0.50**	**0.50**	**1.00**	**0.86**	**0.03**	**0.50**	**1.00**	0.51	0.50	1.00	0.85	0.03	0.50	1.00
RM447	0.51	0.50	1.00	0.86	0.03	0.51	1.00	**0.50**	**0.50**	**1.00**	**0.86**	**0.03**	**0.51**	**1.00**
RM16	0.51	0.50	1.00	0.86	0.03	0.50	1.00	**0.50**	**0.50**	**1.00**	**0.85**	**0.03**	**0.50**	**1.00**
RM214	0.51	0.50	1.00	0.86	0.03	0.50	1.00	0.50	0.50	1.00	0.85	0.03	0.50	1.00

RM3533	-	-	-	-	-	-	-	-	-	-	-	-	-	-
RM211	**0.50**	**0.50**	**1.00**	**0.86**	**0.03**	**0.50**	**1.00**	**0.50**	**0.50**	**1.00**	**0.85**	**0.03**	**0.50**	**1.00**
RM296	**0.50**	**0.50**	**1.00**	**0.87**	**0.03**	**0.50**	**1.00**	0.58	0.50	1.00	0.85	0.03	0.50	1.00
RM84	0.56	0.50	1.00	0.86	0.03	0.50	1.00	**0.50**	**0.50**	**1.00**	**0.84**	**0.03**	**0.50**	**1.00**
RM4128	**0.50**	**0.50**	**1.00**	**0.86**	**0.03**	**0.50**	**1.00**	0.51	0.50	1.00	0.85	0.03	0.50	1.00
RM172	**0.50**	**0.50**	**1.00**	**0.87**	**0.03**	**0.50**	**1.00**	**0.50**	**0.50**	**1.00**	**0.85**	**0.03**	**0.50**	**1.00**
RM5	0.54	0.50	1.00	0.86	0.03	0.50	1.00	0.52	0.50	1.00	0.85	0.03	0.50	1.00
RM168	0.51	0.50	1.00	0.86	0.03	0.50	1.00	**0.50**	**0.50**	**1.00**	**0.86**	**0.03**	**0.51**	**1.00**
RM186	**0.50**	**0.50**	**1.00**	**0.87**	**0.03**	**0.51**	**1.00**	**0.50**	**0.50**	**1.00**	**0.85**	**0.03**	**0.50**	**1.00**
RM520	0.79	0.50	1.00	0.86	0.03	0.50	1.00	0.70	0.50	1.00	0.85	0.03	0.50	1.00
RM8277	**0.50**	**0.50**	**1.00**	**0.86**	**0.03**	**0.50**	**1.00**	**0.50**	**0.50**	**1.00**	**0.86**	**0.03**	**0.50**	**1.00**
RM8243	**0.50**	**0.50**	**1.00**	**0.86**	**0.03**	**0.51**	**1.00**	**0.50**	**0.50**	**1.00**	**0.86**	**0.03**	**0.51**	**1.00**
RM532	0.51	0.50	1.00	0.86	0.03	0.50	1.00	0.53	0.50	1.00	0.85	0.03	0.50	1.00
RM128	0.59	0.50	1.00	0.87	0.02	0.51	1.00	0.55	0.50	1.00	0.85	0.03	0.50	1.00
RM8206	0.52	0.50	1.00	0.86	0.03	0.51	1.00	**0.50**	**0.50**	**1.00**	**0.85**	**0.03**	**0.50**	**1.00**
RM340	**0.50**	**0.50**	**1.00**	**0.85**	**0.03**	**0.50**	**1.00**	0.52	0.50	1.00	0.85	0.03	0.50	1.00
RM587	0.52	0.50	1.00	0.85	0.03	0.50	1.00	0.51	0.50	1.00	0.85	0.03	0.51	1.00
RM530	**0.50**	**0.50**	**1.00**	**0.86**	**0.03**	**0.51**	**1.00**	0.51	0.50	1.00	0.85	0.03	0.50	1.00
RM3117	0.70	0.50	1.00	0.86	0.03	0.51	1.00	0.68	0.50	1.00	0.85	0.03	0.50	1.00
RM295	**0.50**	**0.50**	**1.00**	**0.85**	**0.03**	**0.50**	**1.00**	**0.50**	**0.50**	**1.00**	**0.85**	**0.03**	**0.51**	**1.00**
RM5356	**0.50**	**0.50**	**1.00**	**0.86**	**0.03**	**0.50**	**1.00**	0.50	0.50	1.00	0.85	0.03	0.50	1.00
RM283	0.56	0.50	1.00	0.86	0.03	0.51	1.00	**0.50**	**0.50**	**1.00**	**0.85**	**0.03**	**0.50**	**1.00**
RM7102	**0.50**	**0.50**	**1.00**	**0.87**	**0.03**	**0.51**	**1.00**	**0.50**	**0.50**	**1.00**	**0.85**	**0.03**	**0.50**	**1.00**
RM190	0.52	0.50	1.00	0.87	0.03	0.51	1.00	**0.50**	**0.50**	**1.00**	**0.85**	**0.03**	**0.50**	**1.00**
RM5390	**0.50**	**0.50**	**1.00**	**0.86**	**0.03**	**0.50**	**1.00**	0.52	0.50	1.00	0.85	0.03	0.50	1.00

	F_3 WM-RM3533							F_3 non-WM-RM3533						
	Obs. F	Min F	Max F	Mean*	SE*	L95*	U95*	Obs. F	Min F	Max F	Mean*	SE*	L95*	U95*
RM527	0.52	0.50	1.00	0.86	0.03	0.50	1.00	0.51	0.50	1.00	0.85	0.03	0.51	1.00
RM23	**0.50**	**0.50**	**1.00**	**0.86**	**0.03**	**0.51**	**1.00**	0.55	0.50	1.00	0.85	0.03	0.50	1.00
RM231	0.54	0.50	1.00	0.86	0.03	0.50	1.00	0.51	0.50	1.00	0.84	0.03	0.50	1.00
RM4862	0.50	0.50	1.00	0.86	0.03	0.50	1.00	**0.50**	**0.50**	**1.00**	**0.85**	**0.03**	**0.50**	**1.00**
RM447	**0.50**	**0.50**	**1.00**	**0.86**	**0.03**	**0.50**	**1.00**	0.51	0.50	0.99	0.85	0.03	0.50	0.99
RM16	**0.50**	**0.50**	**1.00**	**0.86**	**0.03**	**0.50**	**1.00**	0.51	0.50	1.00	0.84	0.03	0.50	1.00
RM214	0.50	0.50	1.00	0.87	0.03	0.50	1.00	0.55	0.50	1.00	0.85	0.03	0.50	1.00
RM3533	-	-	-	-	-	-	-	-	-	-	-	-	-	-
RM211	**0.50**	**0.50**	**1.00**	**0.86**	**0.03**	**0.51**	**1.00**	**0.50**	**0.50**	**1.00**	**0.85**	**0.03**	**0.50**	**1.00**
RM296	0.55	0.50	1.00	0.86	0.03	0.51	1.00	0.50	0.50	1.00	0.85	0.03	0.50	1.00
RM84	0.51	0.50	1.00	0.86	0.03	0.50	1.00	0.57	0.50	1.00	0.84	0.03	0.50	1.00
RM4128	**0.51**	**0.50**	**1.00**	**0.86**	**0.03**	**0.51**	**1.00**	**0.50**	**0.50**	**1.00**	**0.85**	**0.03**	**0.50**	**1.00**
RM172	**0.50**	**0.50**	**1.00**	**0.87**	**0.03**	**0.51**	**1.00**	**0.50**	**0.50**	**0.99**	**0.84**	**0.03**	**0.51**	**0.99**
RM5	0.52	0.50	1.00	0.86	0.03	0.51	1.00	0.55	0.50	1.00	0.85	0.03	0.50	1.00
RM168	0.50	0.50	1.00	0.86	0.03	0.50	1.00	0.50	0.50	0.99	0.85	0.03	0.50	0.99
RM186	**0.50**	**0.50**	**1.00**	**0.86**	**0.03**	**0.50**	**1.00**	0.51	0.50	1.00	0.85	0.03	0.51	1.00
RM520	0.72	0.50	1.00	0.86	0.03	0.50	1.00	0.82	0.50	1.00	0.85	0.03	0.50	1.00
RM8277	0.51	0.50	1.00	0.86	0.03	0.50	1.00	**0.50**	**0.50**	**1.00**	**0.85**	**0.03**	**0.50**	**1.00**
RM8243	0.51	0.50	1.00	0.86	0.03	0.50	1.00	**0.50**	**0.50**	**1.00**	**0.84**	**0.03**	**0.51**	**1.00**
RM532	0.52	0.50	1.00	0.85	0.03	0.50	1.00	0.51	0.50	0.99	0.85	0.03	0.50	0.99
RM128	0.55	0.50	1.00	0.86	0.03	0.50	1.00	0.62	0.50	1.00	0.85	0.03	0.50	1.00
RM8206	**0.50**	**0.50**	**1.00**	**0.85**	**0.03**	**0.50**	**1.00**	0.54	0.50	1.00	0.86	0.03	0.50	1.00
RM340	0.51	0.50	1.00	0.86	0.03	0.50	1.00	**0.50**	**0.50**	**1.00**	**0.85**	**0.03**	**0.50**	**1.00**
RM587	0.51	0.50	1.00	0.87	0.03	0.51	1.00	0.53	0.50	1.00	0.85	0.03	0.50	1.00

RM530	**0.50**	**0.50**	**1.00**	**0.86**	**0.03**	**0.50**	**1.00**	0.50	0.50	1.00	0.85	0.03	0.50	1.00
RM3117	0.66	0.50	1.00	0.87	0.03	0.50	1.00	0.76	0.50	1.00	0.85	0.03	0.50	1.00
RM295	**0.50**	**0.50**	**1.00**	**0.86**	**0.03**	**0.50**	**1.00**	**0.50**	**0.50**	**1.00**	**0.85**	**0.03**	**0.51**	**1.00**
RM5356	0.50	0.50	1.00	0.87	0.03	0.51	1.00	0.50	0.50	1.00	0.86	0.03	0.50	1.00
RM283	0.51	0.50	1.00	0.86	0.03	0.50	1.00	0.58	0.50	1.00	0.84	0.03	0.50	1.00
RM7102	**0.50**	**0.50**	**1.00**	**0.86**	**0.03**	**0.50**	**1.00**	**0.50**	**0.50**	**1.00**	**0.86**	**0.03**	**0.51**	**1.00**
RM190	**0.50**	**0.50**	**1.00**	**0.85**	**0.03**	**0.50**	**1.00**	0.55	0.50	1.00	0.85	0.03	0.50	1.00
RM5390	0.51	0.50	1.00	0.86	0.03	0.50	1.00	**0.50**	**0.50**	**1.00**	**0.85**	**0.03**	**0.51**	**1.00**

	F_3 Ideal-1							F_3 Ideal-2						
	Obs. F	Min F	Max F	Mean*	SE*	L95*	U95*	Obs.F	Min F	Max F	Mean*	SE*	L95*	U95*
RM527	0.52	0.50	1.00	0.89	0.02	0.51	1.00	0.51	0.50	1.00	0.89	0.02	0.51	1.00
RM23	0.52	0.50	1.00	0.89	0.03	0.51	1.00	0.51	0.50	1.00	0.89	0.02	0.51	1.00
RM231	0.52	0.50	1.00	0.89	0.02	0.51	1.00	0.52	0.50	1.00	0.89	0.03	0.50	1.00
RM4862	**0.50**	**0.50**	**1.00**	**0.90**	**0.02**	**0.51**	**1.00**	**0.50**	**0.50**	**1.00**	**0.88**	**0.03**	**0.50**	**1.00**
RM447	**0.50**	**0.50**	**1.00**	**0.89**	**0.03**	**0.51**	**1.00**	**0.50**	**0.50**	**1.00**	**0.89**	**0.03**	**0.51**	**1.00**
RM16	**0.50**	**0.50**	**1.00**	**0.89**	**0.03**	**0.50**	**1.00**	**0.50**	**0.50**	**1.00**	**0.89**	**0.03**	**0.51**	**1.00**
RM214	**0.50**	**0.50**	**1.00**	**0.89**	**0.02**	**0.51**	**1.00**	**0.50**	**0.50**	**1.00**	**0.89**	**0.02**	**0.51**	**1.00**
RM3533	**0.50**	**0.50**	**1.00**	**0.90**	**0.02**	**0.51**	**1.00**	**0.50**	**0.50**	**1.00**	**0.89**	**0.03**	**0.50**	**1.00**
RM211	**0.50**	**0.50**	**1.00**	**0.90**	**0.02**	**0.51**	**1.00**	**0.50**	**0.50**	**1.00**	**0.89**	**0.02**	**0.51**	**1.00**
RM296	0.51	0.50	1.00	0.89	0.02	0.51	1.00	0.52	0.50	1.00	0.89	0.02	0.51	1.00
RM84	0.52	0.50	1.00	0.89	0.02	0.51	1.00	0.52	0.50	1.00	0.89	0.02	0.51	1.00
RM4128	**0.51**	**0.50**	**1.00**	**0.89**	**0.02**	**0.51**	**1.00**	**0.50**	**0.50**	**1.00**	**0.88**	**0.03**	**0.50**	**1.00**
RM172	**0.50**	**0.50**	**1.00**	**0.89**	**0.02**	**0.51**	**1.00**	**0.50**	**0.50**	**1.00**	**0.90**	**0.02**	**0.51**	**1.00**
RM5	0.54	0.50	1.00	0.89	0.02	0.51	1.00	0.53	0.50	1.00	0.88	0.03	0.50	1.00
RM168	**0.50**	**0.50**	**1.00**	**0.90**	**0.02**	**0.51**	**1.00**	**0.51**	**0.50**	**1.00**	**0.89**	**0.02**	**0.51**	**1.00**

RM186	**0.50**	**0.50**	**1.00**	**0.89**	**0.03**	**0.50**	**1.00**	**0.50**	**0.50**	**1.00**	**0.89**	**0.02**	**0.50**	**1.00**
RM520	0.75	0.50	1.00	0.89	0.03	0.50	1.00	0.76	0.50	1.00	0.88	0.03	0.51	1.00
RM8277	**0.50**	**0.50**	**1.00**	**0.90**	**0.02**	**0.51**	**1.00**	**0.50**	**0.50**	**1.00**	**0.88**	**0.03**	**0.50**	**1.00**
RM8243	**0.50**	**0.50**	**1.00**	**0.89**	**0.02**	**0.51**	**1.00**	**0.50**	**0.50**	**1.00**	**0.89**	**0.02**	**0.51**	**1.00**
RM532	0.51	0.50	1.00	0.89	0.03	0.51	1.00	0.52	0.50	1.00	0.88	0.03	0.50	1.00
RM128	0.58	0.50	1.00	0.89	0.02	0.51	1.00	0.57	0.50	1.00	0.89	0.02	0.50	1.00
RM8206	0.51	0.50	1.00	0.90	0.02	0.51	1.00	0.50	0.50	1.00	0.89	0.02	0.50	1.00
RM340	**0.50**	**0.50**	**1.00**	**0.90**	**0.02**	**0.51**	**1.00**	**0.50**	**0.50**	**1.00**	**0.88**	**0.03**	**0.51**	**1.00**
RM587	0.52	0.50	1.00	0.89	0.02	0.51	1.00	0.52	0.50	1.00	0.89	0.03	0.50	1.00
RM530	**0.50**	**0.50**	**1.00**	**0.90**	**0.02**	**0.51**	**1.00**	**0.50**	**0.50**	**1.00**	**0.89**	**0.02**	**0.50**	**1.00**
RM3117	0.69	0.50	1.00	0.90	0.03	0.51	1.00	0.68	0.50	1.00	0.89	0.02	0.51	1.00
RM295	**0.50**	**0.50**	**1.00**	**0.89**	**0.02**	**0.51**	**1.00**	**0.50**	**0.50**	**1.00**	**0.88**	**0.03**	**0.50**	**1.00**
RM5356	0.51	0.50	1.00	0.89	0.02	0.51	1.00	0.51	0.50	1.00	0.89	0.02	0.51	1.00
RM283	0.54	0.50	1.00	0.89	0.02	0.51	1.00	0.55	0.50	1.00	0.88	0.03	0.51	1.00
RM7102	0.58	0.50	1.00	0.90	0.02	0.51	1.00	0.59	0.50	1.00	0.89	0.03	0.50	1.00
RM190	**0.50**	**0.50**	**1.00**	**0.89**	**0.03**	**0.50**	**1.00**	**0.50**	**0.50**	**1.00**	**0.88**	**0.02**	**0.51**	**1.00**
RM5390	0.51	0.50	1.00	0.88	0.03	0.51	1.00	0.51	0.50	1.00	0.88	0.03	0.51	1.00

* Obs. F indicates observed allele frequencies; Nul. F indicates null allele frequencies; L95 and U95 indicate the lower and upper limits of the 95% confidence interval of Nul. F, respectively. If Obs. F is within this confidence interval, the locus is neutral; otherwise, it is not.

Supplementary Table 5 Parent-allele frequencies (crop : wild) in F_2 and F_3 crop-wild hybrid lineages with (CM) or without (non-CM) the crop-parent markers; and with (WM) or without (non-WM) the wild-parent markers, using the ideal groups as a reference. Frequencies in bold indicate significant differences (*P* <0.05) between lineages.

Primer name	F_2 CM-RM572	F_2 non-CM-RM572	F_2 WM-RM572	F_2 non-WM-RM572
RM84	**0.33 : 0.67**	**0.52 : 0.48**	**0.43 : 0.57**	**0.29 : 0.71**
RM575	**0.53 : 0.47**	**0.19 : 0.81**	**0.32 : 0.68**	**0.63 : 0.37**
RM572	-	-	-	-
RM9	**0.51 : 0.49**	**0.43 : 0.57**	**0.42 : 0.58**	**0.62 : 0.38**
RM5	**0.52 : 0.48**	**0.42 : 0.58**	**0.44 : 0.56**	**0.61 : 0.39**
RM212	**0.40 : 0.60**	**0.34 : 0.66**	**0.36 : 0.64**	**0.42 : 0.58**
RM6895	**0.50 : 0.50**	**0.56 : 0.44**	0.53 : 0.47	0.50 : 0.50
RM7245	0.52 : 0.48	0.57 : 0.43	0.54 : 0.46	0.54 : 0.46
RM523	**0.55 : 0.45**	**0.48 : 0.52**	0.51 : 0.49	0.55 : 0.45
RM282	0.38 : 0.62	0.35 : 0.65	0.37 : 0.63	0.38 : 0.62
RM16	0.60 : 0.40	0.56 : 0.44	**0.56 : 0.44**	**0.64 : 0.36**
RM186	**0.33 : 0.67**	**0.26 : 0.74**	**0.27 : 0.73**	**0.38 : 0.62**
RM468	0.34 : 0.66	0.30 : 0.70	0.31 : 0.69	0.35 : 0.65
RM6395	0.52 : 0.48	0.50 : 0.50	0.51 : 0.49	0.53 : 0.47
RM6089	**0.58 : 0.42**	**0.44 : 0.56**	**0.49 : 0.51**	**0.64 : 0.36**
RM3558	**0.49 : 0.51**	**0.37 : 0.63**	**0.42 : 0.58**	**0.53 : 0.47**
RM6748	0.51 : 0.49	0.46 : 0.54	0.50 : 0.50	0.48 : 0.52
RM3419	**0.58 : 0.42**	**0.51 : 0.49**	0.55 : 0.45	0.58 : 0.42
RM6841	0.73 : 0.27	0.67 : 0.33	0.70 : 0.30	0.72 : 0.28
RM190	0.23 : 0.77	0.18 : 0.82	**0.19 : 0.81**	**0.26 : 0.74**
RM276	**0.52 : 0.48**	**0.43 : 0.57**	**0.46 : 0.54**	**0.57 : 0.43**

RM214	0.33 : 0.67	0.31 : 0.69	0.32 : 0.68	0.34 : 0.66
RM11	0.46 : 0.54	0.44 : 0.56	0.44 : 0.56	0.48 : 0.52
RM505	0.59 : 0.41	0.57 : 0.43	0.59 : 0.41	0.57 : 0.43
RM172	0.51 : 0.49	0.46 : 0.54	0.49 : 0.51	0.49 : 0.51
RM408	0.61 : 0.39	0.56 : 0.44	0.59 : 0.41	0.61 : 0.39
RM5068	0.17 : 0.83	0.17 : 0.83	0.16 : 0.84	0.18 : 0.82
RM331	0.60 : 0.40	0.60 : 0.40	**0.58 : 0.42**	**0.65 : 0.35**
RM296	0.55 : 0.45	0.57 : 0.43	0.55 : 0.45	0.59 : 0.41
RM4455	**0.42 : 0.58**	**0.35 : 0.65**	**0.37 : 0.63**	**0.45 : 0.55**
RM7557	**0.45 : 0.55**	**0.39 : 0.61**	0.41 : 0.59	0.48 : 0.52
RM332	0.47 : 0.53	0.47 : 0.53	0.46 : 0.54	0.50 : 0.50
RM4862	0.82 : 0.18	0.82 : 0.18	0.83 : 0.17	0.79 : 0.21
RM224	0.36 : 0.64	0.37 : 0.63	0.36 : 0.64	0.37 : 0.63
RM8216	**0.51 : 0.49**	**0.61 : 0.39**	0.55 : 0.45	0.53 : 0.47
RM3483	0.56 : 0.44	0.55 : 0.45	**0.52 : 0.48**	**0.65 : 0.35**
RM6296	**0.52 : 0.48**	**0.58 : 0.42**	0.55 : 0.45	0.51 : 0.49
RM277	0.53 : 0.47	0.50 : 0.50	0.51 : 0.49	0.55 : 0.45
RM6732	**0.34 : 0.66**	**0.21 : 0.79**	0.29 : 0.71	0.30 : 0.70
RM17	0.34 : 0.66	0.30 : 0.70	0.33 : 0.67	0.32 : 0.68
RM111	0.41 : 0.59	0.37 : 0.63	**0.38 : 0.62**	**0.45 : 0.55**
RM143	**0.59 : 0.41**	**0.53 : 0.47**	0.55 : 0.45	0.60 : 0.40
RM333	0.51 : 0.49	0.53 : 0.47	0.53 : 0.47	0.50 : 0.50
RM4128	**0.56 : 0.44**	**0.47 : 0.53**	0.51 : 0.49	0.57 : 0.43
RM168	**0.35 : 0.65**	**0.25 : 0.75**	**0.29 : 0.71**	**0.38 : 0.62**
RM565	0.37 : 0.63	0.32 : 0.68	0.34 : 0.66	0.38 : 0.62
RM16074	0.50 : 0.50	0.47 : 0.53	**0.47 : 0.53**	**0.53 : 0.47**

RM20468	0.50 : 0.50	0.49 : 0.51	0.49 : 0.51	0.51 : 0.49
RM130	0.50 : 0.50	0.48 : 0.52	**0.47 : 0.53**	**0.54 : 0.46**
RM20460	0.52 : 0.48	0.47 : 0.53	**0.48 : 0.52**	**0.54 : 0.46**
RM5371	0.57 : 0.43	0.53 : 0.47	0.54 : 0.46	0.58 : 0.42
RM16071	**0.58 : 0.42**	**0.50 : 0.50**	**0.53 : 0.47**	**0.61 : 0.39**
Average	0.48 : 0.52	0.44 : 0.56	0.45 : 0.55	0.50 : 0.50

Primer name	F_3 CM-RM572	F_3 non-CM-RM572	F_3 WM-RM572	F_3 non-WM-RM572
RM84	**0.39 : 0.61**	**0.31 : 0.69**	0.36 : 0.64	0.36 : 0.64
RM575	**0.56 : 0.44**	**0.33 : 0.67**	**0.41 : 0.59**	**0.57 : 0.43**
RM572	-	-	-	-
RM9	**0.47 : 0.53**	**0.39 : 0.61**	0.44 : 0.56	0.43 : 0.57
RM5	**0.53 : 0.47**	**0.38 : 0.62**	**0.45 : 0.55**	**0.52 : 0.48**
RM212	0.42 : 0.58	0.39 : 0.61	0.40 : 0.60	0.42 : 0.58
RM6895	0.67 : 0.33	0.62 : 0.38	**0.63 : 0.37**	**0.68 : 0.32**
RM7245	0.56 : 0.44	0.56 : 0.44	0.57 : 0.43	0.56 : 0.44
RM523	0.63 : 0.37	0.61 : 0.39	0.63 : 0.37	0.61 : 0.39
RM282	0.29 : 0.71	0.26 : 0.74	0.27 : 0.73	0.30 : 0.70
RM16	0.62 : 0.38	0.66 : 0.34	**0.66 : 0.34**	**0.59 : 0.41**
RM186	0.30 : 0.70	0.29 : 0.71	0.29 : 0.71	0.31 : 0.69
RM468	0.33 : 0.67	0.33 : 0.67	0.32 : 0.68	0.34 : 0.66
RM6395	**0.33 : 0.67**	**0.44 : 0.56**	0.39 : 0.61	0.34 : 0.66
RM6089	**0.57 : 0.43**	**0.48 : 0.52**	0.52 : 0.48	0.56 : 0.44
RM3558	0.45 : 0.55	0.43 : 0.57	0.44 : 0.56	0.46 : 0.54
RM6748	0.48 : 0.52	0.46 : 0.54	0.48 : 0.52	0.46 : 0.54
RM3419	0.54 : 0.46	0.56 : 0.44	0.56 : 0.44	0.52 : 0.48

RM6841	0.72 : 0.28	0.73 : 0.27	0.74 : 0.26	0.70 : 0.30
RM190	0.27 : 0.73	0.29 : 0.71	0.28 : 0.72	0.27 : 0.73
RM276	0.49 : 0.51	0.49 : 0.51	0.47 : 0.53	0.51 : 0.49
RM214	0.31 : 0.69	0.27 : 0.73	**0.26 : 0.74**	**0.34 : 0.66**
RM11	0.56 : 0.44	0.54 : 0.46	0.56 : 0.44	0.53 : 0.47
RM505	**0.68 : 0.32**	**0.62 : 0.38**	0.64 : 0.36	0.69 : 0.31
RM172	0.53 : 0.47	0.51 : 0.49	0.51 : 0.49	0.54 : 0.46
RM408	0.49 : 0.51	0.52 : 0.48	0.51 : 0.49	0.49 : 0.51
RM5068	**0.24 : 0.76**	**0.18 : 0.82**	**0.20 : 0.80**	**0.25 : 0.75**
RM331	**0.65 : 0.35**	**0.55 : 0.45**	**0.58 : 0.42**	**0.66 : 0.34**
RM296	0.61 : 0.39	0.57 : 0.43	0.58 : 0.42	0.60 : 0.40
RM4455	0.60 : 0.40	0.64 : 0.36	**0.64 : 0.36**	**0.57 : 0.43**
RM7557	0.45 : 0.55	0.46 : 0.54	0.47 : 0.53	0.43 : 0.57
RM332	0.60 : 0.40	0.58 : 0.42	0.58 : 0.42	0.61 : 0.39
RM4862	0.80 : 0.20	0.77 : 0.23	0.79 : 0.21	0.78 : 0.22
RM224	0.40 : 0.60	0.45 : 0.55	0.44 : 0.56	0.39 : 0.61
RM8216	0.66 : 0.34	0.66 : 0.34	0.66 : 0.34	0.66 : 0.34
RM3483	0.56 : 0.44	0.61 : 0.39	0.59 : 0.41	0.56 : 0.44
RM6296	0.66 : 0.34	0.70 : 0.30	0.70 : 0.30	0.65 : 0.35
RM277	0.55 : 0.45	0.51 : 0.49	0.54 : 0.46	0.53 : 0.47
RM6732	0.35 : 0.65	0.34 : 0.66	0.35 : 0.65	0.34 : 0.66
RM17	0.45 : 0.55	0.46 : 0.54	0.46 : 0.54	0.45 : 0.55
RM111	0.59 : 0.41	0.65 : 0.35	**0.64 : 0.36**	**0.58 : 0.42**
RM143	0.53 : 0.47	0.51 : 0.49	0.51 : 0.49	0.54 : 0.46
RM333	**0.54 : 0.46**	**0.45 : 0.55**	**0.48 : 0.52**	**0.55 : 0.45**
RM4128	0.41 : 0.59	0.46 : 0.54	0.45 : 0.55	0.40 : 0.60

RM168	0.35 : 0.65	0.31 : 0.69	0.32 : 0.68	0.36 : 0.64
RM565	0.62 : 0.38	0.62 : 0.38	0.62 : 0.38	0.62 : 0.38
RM16074	0.53 : 0.47	0.49 : 0.51	0.52 : 0.48	0.52 : 0.48
RM20468	0.56 : 0.44	0.59 : 0.41	0.59 : 0.41	0.56 : 0.44
RM130	0.56 : 0.44	0.52 : 0.48	0.54 : 0.46	0.54 : 0.46
RM20460	**0.55 : 0.45**	**0.61 : 0.39**	0.59 : 0.41	0.55 : 0.45
RM5371	0.59 : 0.41	0.63 : 0.37	0.62 : 0.38	0.59 : 0.41
RM16071	0.55 : 0.45	0.54 : 0.46	0.55 : 0.45	0.54 : 0.46
Average	0.51 : 0.49	0.49 : 0.51	0.50 : 0.50	0.50 : 0.50

Primer name	F_2 CM-RM408	F_2 non-CM-RM408	F_2 WM-RM408	F_2 non-WM-RM408
RM84	0.39 : 0.61	0.42 : 0.58	**0.36 : 0.64**	**0.45 : 0.55**
RM575	**0.43 : 0.57**	**0.33 : 0.67**	0.41 : 0.59	0.42 : 0.58
RM572	**0.49 : 0.51**	**0.37 : 0.63**	0.47 : 0.53	0.48 : 0.52
RM9	**0.47 : 0.53**	**0.55 : 0.45**	0.48 : 0.52	0.47 : 0.53
RM5	0.48 : 0.52	0.53 : 0.47	0.48 : 0.52	0.50 : 0.50
RM212	0.37 : 0.63	0.41 : 0.59	0.36 : 0.64	0.40 : 0.60
RM6895	0.52 : 0.48	0.52 : 0.48	0.52 : 0.48	0.52 : 0.48
RM7245	0.54 : 0.46	0.53 : 0.47	0.54 : 0.46	0.52 : 0.48
RM523	0.53 : 0.47	0.49 : 0.51	**0.55 : 0.45**	**0.47 : 0.53**
RM282	0.37 : 0.63	0.34 : 0.66	0.38 : 0.62	0.34 : 0.66
RM16	0.59 : 0.41	0.57 : 0.43	0.59 : 0.41	0.57 : 0.43
RM186	0.30 : 0.70	0.35 : 0.65	0.31 : 0.69	0.29 : 0.71
RM468	0.33 : 0.67	0.31 : 0.69	0.33 : 0.67	0.30 : 0.70
RM6395	0.52 : 0.48	0.48 : 0.52	0.53 : 0.47	0.48 : 0.52
RM6089	0.50 : 0.50	0.54 : 0.46	0.49 : 0.51	0.52 : 0.48

RM3558	**0.51 : 0.49**	**0.43 : 0.57**	0.49 : 0.51	0.52 : 0.48
RM6748	0.50 : 0.50	0.50 : 0.50	**0.53 : 0.47**	**0.44 : 0.56**
RM3419	0.55 : 0.45	0.57 : 0.43	0.57 : 0.43	0.53 : 0.47
RM6841	0.71 : 0.29	0.70 : 0.30	**0.73 : 0.27**	**0.67 : 0.33**
RM190	0.20 : 0.80	0.25 : 0.75	0.22 : 0.78	0.19 : 0.81
RM276	0.52 : 0.48	0.47 : 0.53	**0.55 : 0.45**	**0.44 : 0.56**
RM214	**0.36 : 0.64**	**0.15 : 0.85**	0.33 : 0.67	0.33 : 0.67
RM11	**0.48 : 0.52**	**0.30 : 0.70**	**0.41 : 0.59**	**0.48 : 0.52**
RM505	**0.60 : 0.40**	**0.46 : 0.54**	0.59 : 0.41	0.58 : 0.42
RM172	**0.50 : 0.50**	**0.44 : 0.56**	0.48 : 0.52	0.51 : 0.49
RM408	-	-	-	-
RM5068	**0.18 : 0.82**	**0.07 : 0.93**	**0.13 : 0.87**	**0.24 : 0.76**
RM331	0.59 : 0.41	0.62 : 0.38	0.61 : 0.39	0.57 : 0.43
RM296	0.56 : 0.44	0.59 : 0.41	0.56 : 0.44	0.56 : 0.44
RM4455	0.39 : 0.61	0.43 : 0.57	0.40 : 0.60	0.37 : 0.63
RM7557	**0.44 : 0.56**	**0.38 : 0.62**	0.43 : 0.57	0.43 : 0.57
RM332	**0.48 : 0.52**	**0.40 : 0.60**	0.46 : 0.54	0.49 : 0.51
RM4862	0.83 : 0.17	0.77 : 0.23	**0.80 : 0.20**	**0.87 : 0.13**
RM224	0.36 : 0.64	0.38 : 0.62	0.35 : 0.65	0.40 : 0.60
RM8216	0.55 : 0.45	0.51 : 0.49	0.53 : 0.47	0.58 : 0.42
RM3483	0.56 : 0.44	0.55 : 0.45	**0.51 : 0.49**	**0.58 : 0.42**
RM6296	0.54 : 0.46	0.58 : 0.42	0.53 : 0.47	0.57 : 0.43
RM277	0.51 : 0.49	0.54 : 0.46	0.54 : 0.46	0.48 : 0.52
RM6732	**0.31 : 0.69**	**0.22 : 0.78**	0.29 : 0.71	0.30 : 0.70
RM17	0.32 : 0.68	0.34 : 0.66	0.34 : 0.66	0.30 : 0.70
RM111	0.39 : 0.61	0.42 : 0.58	0.38 : 0.62	0.43 : 0.57

RM143	0.57 : 0.43	0.56 : 0.44	0.58 : 0.42	0.54 : 0.46
RM333	0.52 : 0.48	0.51 : 0.49	**0.50 : 0.50**	**0.56 : 0.44**
RM4128	0.53 : 0.47	0.49 : 0.51	**0.59 : 0.41**	**0.41 : 0.59**
RM168	0.32 : 0.68	0.31 : 0.69	**0.28 : 0.72**	**0.34 : 0.66**
RM565	0.35 : 0.65	0.36 : 0.64	0.35 : 0.65	0.34 : 0.66
RM16074	0.49 : 0.51	0.50 : 0.50	0.49 : 0.51	0.48 : 0.52
RM20468	**0.49 : 0.51**	**0.55 : 0.45**	0.51 : 0.49	0.46 : 0.54
RM130	0.49 : 0.51	0.52 : 0.48	0.50 : 0.50	0.48 : 0.52
RM20460	**0.51 : 0.49**	**0.45 : 0.55**	0.51 : 0.49	0.48 : 0.52
RM5371	0.56 : 0.44	0.51 : 0.49	0.55 : 0.45	0.56 : 0.44
RM16071	0.55 : 0.45	0.56 : 0.44	0.57 : 0.43	0.52 : 0.48
Average	0.47 : 0.53	0.45 : 0.55	0.47 : 0.53	0.46 : 0.54

Primer name	F_3 CM-RM408	F_3 non-CM-RM408	F_3 WM-RM408	F_3 non-WM-RM408
RM84	**0.38 : 0.62**	**0.32 : 0.68**	**0.34 : 0.66**	**0.40 : 0.60**
RM575	0.49 : 0.51	0.44 : 0.56	0.47 : 0.53	0.49 : 0.51
RM572	0.53 : 0.47	0.54 : 0.46	0.54 : 0.46	0.50 : 0.50
RM9	**0.46 : 0.54**	**0.40 : 0.60**	**0.41 : 0.59**	**0.50 : 0.50**
RM5	0.48 : 0.52	0.45 : 0.55	0.45 : 0.55	0.50 : 0.50
RM212	0.42 : 0.58	0.39 : 0.61	0.40 : 0.60	0.44 : 0.56
RM6895	0.64 : 0.36	0.66 : 0.34	0.65 : 0.35	0.64 : 0.36
RM7245	0.57 : 0.43	0.56 : 0.44	0.56 : 0.44	0.57 : 0.43
RM523	0.62 : 0.38	0.63 : 0.37	0.63 : 0.37	0.61 : 0.39
RM282	0.29 : 0.71	0.25 : 0.75	0.28 : 0.72	0.28 : 0.72
RM16	0.63 : 0.37	0.64 : 0.36	0.63 : 0.37	0.64 : 0.36
RM186	0.31 : 0.69	0.27 : 0.73	**0.28 : 0.72**	**0.34 : 0.66**

RM468	0.33 : 0.67	0.32 : 0.68	0.31 : 0.69	0.36 : 0.64
RM6395	0.37 : 0.63	0.35 : 0.65	0.36 : 0.64	0.38 : 0.62
RM6089	0.54 : 0.46	0.53 : 0.47	0.54 : 0.46	0.53 : 0.47
RM3558	**0.45 : 0.55**	**0.37 : 0.63**	0.42 : 0.58	0.44 : 0.56
RM6748	0.47 : 0.53	0.46 : 0.54	0.48 : 0.52	0.46 : 0.54
RM3419	**0.57 : 0.43**	**0.49 : 0.51**	**0.52 : 0.48**	**0.59 : 0.41**
RM6841	0.71 : 0.29	0.76 : 0.24	**0.74 : 0.26**	**0.68 : 0.32**
RM190	**0.30 : 0.70**	**0.23 : 0.77**	0.27 : 0.73	0.30 : 0.70
RM276	**0.53 : 0.47**	**0.40 : 0.60**	0.47 : 0.53	0.52 : 0.48
RM214	0.31 : 0.69	0.26 : 0.74	**0.28 : 0.72**	**0.34 : 0.66**
RM11	**0.58 : 0.42**	**0.47 : 0.53**	**0.52 : 0.48**	**0.61 : 0.39**
RM505	0.66 : 0.34	0.65 : 0.35	0.65 : 0.35	0.69 : 0.31
RM172	**0.50 : 0.50**	**0.57 : 0.43**	0.53 : 0.47	0.50 : 0.50
RM408	-	-	-	-
RM5068	**0.25 : 0.75**	**0.15 : 0.85**	**0.15 : 0.85**	**0.38 : 0.62**
RM331	0.61 : 0.39	0.62 : 0.38	0.61 : 0.39	0.61 : 0.39
RM296	0.59 : 0.41	0.60 : 0.40	**0.57 : 0.43**	**0.63 : 0.37**
RM4455	**0.63 : 0.37**	**0.57 : 0.43**	0.62 : 0.38	0.59 : 0.41
RM7557	**0.48 : 0.52**	**0.39 : 0.61**	0.44 : 0.56	0.47 : 0.53
RM332	**0.61 : 0.39**	**0.55 : 0.45**	0.58 : 0.42	0.62 : 0.38
RM4862	**0.81 : 0.19**	**0.74 : 0.26**	0.79 : 0.21	0.80 : 0.20
RM224	0.43 : 0.57	0.40 : 0.60	0.41 : 0.59	0.44 : 0.56
RM8216	**0.70 : 0.30**	**0.59 : 0.41**	0.65 : 0.35	0.69 : 0.31
RM3483	**0.61 : 0.39**	**0.51 : 0.49**	**0.56 : 0.44**	**0.62 : 0.38**
RM6296	**0.70 : 0.30**	**0.63 : 0.37**	**0.66 : 0.34**	**0.72 : 0.28**
RM277	**0.57 : 0.43**	**0.46 : 0.54**	**0.50 : 0.50**	**0.63 : 0.37**

RM6732	0.36 : 0.64	0.31 : 0.69	0.34 : 0.66	0.36 : 0.64
RM17	0.44 : 0.56	0.49 : 0.51	**0.48 : 0.52**	**0.40 : 0.60**
RM111	**0.65 : 0.35**	**0.53 : 0.47**	0.59 : 0.41	0.66 : 0.34
RM143	0.52 : 0.48	0.53 : 0.47	0.52 : 0.48	0.53 : 0.47
RM333	0.50 : 0.50	0.53 : 0.47	**0.53 : 0.47**	**0.46 : 0.54**
RM4128	0.44 : 0.56	0.40 : 0.60	0.42 : 0.58	0.45 : 0.55
RM168	**0.36 : 0.64**	**0.26 : 0.74**	**0.31 : 0.69**	**0.38 : 0.62**
RM565	0.62 : 0.38	0.63 : 0.37	0.63 : 0.37	0.59 : 0.41
RM16074	0.52 : 0.48	0.52 : 0.48	0.52 : 0.48	0.50 : 0.50
RM20468	0.57 : 0.43	0.58 : 0.42	0.57 : 0.43	0.57 : 0.43
RM130	0.53 : 0.47	0.56 : 0.44	0.55 : 0.45	0.53 : 0.47
RM20460	0.57 : 0.43	0.57 : 0.43	0.58 : 0.42	0.56 : 0.44
RM5371	0.61 : 0.39	0.60 : 0.40	0.61 : 0.39	0.59 : 0.41
RM16071	0.55 : 0.45	0.55 : 0.45	0.55 : 0.45	0.53 : 0.47
Average	0.51 : 0.49	0.48 : 0.52	0.50 : 0.50	0.52 : 0.48

Primer name	F_2 CM-RM277	F_2 non-CM-RM277	F_2 WM-RM277	F_2 non-WM-RM277
RM84	**0.37 : 0.63**	**0.47 : 0.53**	0.39 : 0.61	0.39 : 0.61
RM575	0.41 : 0.59	0.41 : 0.59	0.42 : 0.58	0.39 : 0.61
RM572	**0.52 : 0.48**	**0.36 : 0.64**	**0.43 : 0.57**	**0.60 : 0.40**
RM9	**0.51 : 0.49**	**0.34 : 0.66**	**0.42 : 0.58**	**0.59 : 0.41**
RM5	0.49 : 0.51	0.49 : 0.51	0.48 : 0.52	0.51 : 0.49
RM212	**0.42 : 0.58**	**0.27 : 0.73**	**0.34 : 0.66**	**0.48 : 0.52**
RM6895	**0.51 : 0.49**	**0.58 : 0.42**	0.51 : 0.49	0.55 : 0.45
RM7245	**0.55 : 0.45**	**0.49 : 0.51**	0.53 : 0.47	0.57 : 0.43
RM523	0.52 : 0.48	0.53 : 0.47	0.52 : 0.48	0.54 : 0.46

RM282	0.37 : 0.63	0.37 : 0.63	0.38 : 0.62	0.34 : 0.66
RM16	0.58 : 0.42	0.59 : 0.41	0.57 : 0.43	0.62 : 0.38
RM186	0.30 : 0.70	0.31 : 0.69	**0.28 : 0.72**	**0.38 : 0.62**
RM468	0.32 : 0.68	0.35 : 0.65	0.32 : 0.68	0.35 : 0.65
RM6395	0.51 : 0.49	0.50 : 0.50	**0.50 : 0.50**	**0.56 : 0.44**
RM6089	**0.48 : 0.52**	**0.61 : 0.39**	**0.49 : 0.51**	**0.55 : 0.45**
RM3558	**0.52 : 0.48**	**0.28 : 0.72**	**0.41 : 0.59**	**0.57 : 0.43**
RM6748	**0.53 : 0.47**	**0.36 : 0.64**	0.50 : 0.50	0.48 : 0.52
RM3419	0.56 : 0.44	0.53 : 0.47	0.56 : 0.44	0.54 : 0.46
RM6841	0.72 : 0.28	0.67 : 0.33	0.71 : 0.29	0.69 : 0.31
RM190	**0.22 : 0.78**	**0.16 : 0.84**	**0.20 : 0.80**	**0.26 : 0.74**
RM276	0.51 : 0.49	0.53 : 0.47	0.52 : 0.48	0.49 : 0.51
RM214	**0.37 : 0.63**	**0.22 : 0.78**	**0.31 : 0.69**	**0.37 : 0.63**
RM11	**0.48 : 0.52**	**0.35 : 0.65**	**0.43 : 0.57**	**0.53 : 0.47**
RM505	0.59 : 0.41	0.57 : 0.43	0.58 : 0.42	0.60 : 0.40
RM172	0.49 : 0.51	0.50 : 0.50	0.50 : 0.50	0.47 : 0.53
RM408	**0.59 : 0.41**	**0.25 : 0.75**	**0.41 : 0.59**	**0.71 : 0.29**
RM5068	**0.19 : 0.81**	**0.12 : 0.88**	0.16 : 0.84	0.18 : 0.82
RM331	**0.61 : 0.39**	**0.55 : 0.45**	0.60 : 0.40	0.58 : 0.42
RM296	0.56 : 0.44	0.57 : 0.43	0.55 : 0.45	0.58 : 0.42
RM4455	**0.41 : 0.59**	**0.34 : 0.66**	0.38 : 0.62	0.42 : 0.58
RM7557	**0.44 : 0.56**	**0.38 : 0.62**	**0.41 : 0.59**	**0.48 : 0.52**
RM332	0.47 : 0.53	0.47 : 0.53	**0.45 : 0.55**	**0.53 : 0.47**
RM4862	0.82 : 0.18	0.83 : 0.17	0.82 : 0.18	0.83 : 0.17
RM224	0.36 : 0.64	0.37 : 0.63	0.35 : 0.65	0.40 : 0.60
RM8216	0.53 : 0.47	0.58 : 0.42	0.54 : 0.46	0.54 : 0.46

RM3483	**0.58 : 0.42**	**0.47 : 0.53**	**0.53 : 0.47**	**0.65 : 0.35**
RM6296	**0.50 : 0.50**	**0.73 : 0.27**	**0.60 : 0.40**	**0.35 : 0.65**
RM277	-	-	-	-
RM6732	0.24 : 0.76	0.21 : 0.79	0.22 : 0.78	0.27 : 0.73
RM17	**0.30 : 0.70**	**0.42 : 0.58**	0.32 : 0.68	0.36 : 0.64
RM111	**0.34 : 0.66**	**0.56 : 0.44**	**0.41 : 0.59**	**0.33 : 0.67**
RM143	0.56 : 0.44	0.57 : 0.43	0.57 : 0.43	0.55 : 0.45
RM333	**0.54 : 0.46**	**0.44 : 0.56**	0.52 : 0.48	0.52 : 0.48
RM4128	0.53 : 0.47	0.51 : 0.49	0.52 : 0.48	0.55 : 0.45
RM168	**0.33 : 0.67**	**0.27 : 0.73**	**0.29 : 0.71**	**0.41 : 0.59**
RM565	0.35 : 0.65	0.36 : 0.64	0.35 : 0.65	0.36 : 0.64
RM16074	0.48 : 0.52	0.51 : 0.49	0.48 : 0.52	0.51 : 0.49
RM20468	0.50 : 0.50	0.47 : 0.53	0.49 : 0.51	0.52 : 0.48
RM130	0.49 : 0.51	0.50 : 0.50	0.48 : 0.52	0.52 : 0.48
RM20460	0.51 : 0.49	0.47 : 0.53	0.49 : 0.51	0.53 : 0.47
RM5371	0.56 : 0.44	0.53 : 0.47	0.55 : 0.45	0.57 : 0.43
RM16071	0.55 : 0.45	0.56 : 0.44	0.55 : 0.45	0.56 : 0.44
Average	0.47 : 0.53	0.44 : 0.56	0.45 : 0.55	0.49 : 0.51

Primer name	F_3 CM-RM277	F_3 non-CM-RM277	F_3 WM-RM277	F_3 non-WM-RM277
RM84	0.37 : 0.63	0.33 : 0.67	0.37 : 0.63	0.34 : 0.66
RM575	0.49 : 0.51	0.45 : 0.55	0.47 : 0.53	0.50 : 0.50
RM572	**0.56 : 0.44**	**0.46 : 0.54**	**0.51 : 0.49**	**0.58 : 0.42**
RM9	0.45 : 0.55	0.40 : 0.60	**0.42 : 0.58**	**0.48 : 0.52**
RM5	**0.49 : 0.51**	**0.41 : 0.59**	**0.44 : 0.56**	**0.54 : 0.46**
RM212	0.40 : 0.60	0.44 : 0.56	0.40 : 0.60	0.42 : 0.58

RM6895	**0.68 : 0.32**	**0.56 : 0.44**	0.66 : 0.34	0.63 : 0.37
RM7245	0.57 : 0.43	0.55 : 0.45	0.56 : 0.44	0.59 : 0.41
RM523	0.64 : 0.36	0.59 : 0.41	0.63 : 0.37	0.62 : 0.38
RM282	0.28 : 0.72	0.29 : 0.71	0.29 : 0.71	0.27 : 0.73
RM16	0.63 : 0.37	0.63 : 0.37	0.62 : 0.38	0.66 : 0.34
RM186	0.30 : 0.70	0.29 : 0.71	0.28 : 0.72	0.33 : 0.67
RM468	0.33 : 0.67	0.32 : 0.68	**0.31 : 0.69**	**0.37 : 0.63**
RM6395	0.37 : 0.63	0.35 : 0.65	**0.34 : 0.66**	**0.43 : 0.57**
RM6089	0.53 : 0.47	0.55 : 0.45	0.54 : 0.46	0.53 : 0.47
RM3558	**0.46 : 0.54**	**0.40 : 0.60**	0.45 : 0.55	0.43 : 0.57
RM6748	0.47 : 0.53	0.49 : 0.51	**0.45 : 0.55**	**0.52 : 0.48**
RM3419	**0.57 : 0.43**	**0.45 : 0.55**	0.54 : 0.46	0.56 : 0.44
RM6841	**0.74 : 0.26**	**0.67 : 0.33**	0.72 : 0.28	0.72 : 0.28
RM190	0.29 : 0.71	0.24 : 0.76	0.29 : 0.71	0.26 : 0.74
RM276	**0.51 : 0.49**	**0.40 : 0.60**	0.49 : 0.51	0.47 : 0.53
RM214	**0.30 : 0.70**	**0.26 : 0.74**	0.29 : 0.71	0.29 : 0.71
RM11	**0.57 : 0.43**	**0.49 : 0.51**	0.56 : 0.44	0.53 : 0.47
RM505	0.66 : 0.34	0.65 : 0.35	0.65 : 0.35	0.68 : 0.32
RM172	**0.51 : 0.49**	**0.56 : 0.44**	0.52 : 0.48	0.52 : 0.48
RM408	**0.53 : 0.47**	**0.43 : 0.57**	**0.47 : 0.53**	**0.59 : 0.41**
RM5068	**0.25 : 0.75**	**0.14 : 0.86**	**0.17 : 0.83**	**0.33 : 0.67**
RM331	0.62 : 0.38	0.57 : 0.43	**0.59 : 0.41**	**0.66 : 0.34**
RM296	0.59 : 0.41	0.58 : 0.42	0.58 : 0.42	0.62 : 0.38
RM4455	0.61 : 0.39	0.63 : 0.37	**0.63 : 0.37**	**0.57 : 0.43**
RM7557	0.46 : 0.54	0.41 : 0.59	0.45 : 0.55	0.46 : 0.54
RM332	0.60 : 0.40	0.57 : 0.43	0.60 : 0.40	0.59 : 0.41

RM4862	0.80 : 0.20	0.77 : 0.23	0.78 : 0.22	0.81 : 0.19
RM224	**0.43 : 0.57**	**0.37 : 0.63**	0.41 : 0.59	0.43 : 0.57
RM8216	**0.68 : 0.32**	**0.60 : 0.40**	0.65 : 0.35	0.70 : 0.30
RM3483	**0.61 : 0.39**	**0.48 : 0.52**	**0.54 : 0.46**	**0.68 : 0.32**
RM6296	**0.72 : 0.28**	**0.53 : 0.47**	**0.60 : 0.40**	**0.86 : 0.14**
RM277	-	-	-	-
RM6732	**0.33 : 0.67**	**0.40 : 0.60**	**0.36 : 0.64**	**0.29 : 0.71**
RM17	0.46 : 0.54	0.44 : 0.56	0.46 : 0.54	0.45 : 0.55
RM111	**0.63 : 0.37**	**0.55 : 0.45**	0.61 : 0.39	0.61 : 0.39
RM143	0.53 : 0.47	0.49 : 0.51	**0.49 : 0.51**	**0.60 : 0.40**
RM333	0.51 : 0.49	0.50 : 0.50	0.50 : 0.50	0.53 : 0.47
RM4128	**0.41 : 0.59**	**0.50 : 0.50**	0.44 : 0.56	0.41 : 0.59
RM168	0.34 : 0.66	0.33 : 0.67	0.32 : 0.68	0.36 : 0.64
RM565	0.62 : 0.38	0.62 : 0.38	**0.65 : 0.35**	**0.55 : 0.45**
RM16074	**0.53 : 0.47**	**0.47 : 0.53**	0.51 : 0.49	0.54 : 0.46
RM20468	0.58 : 0.42	0.56 : 0.44	**0.59 : 0.41**	**0.53 : 0.47**
RM130	0.55 : 0.45	0.51 : 0.49	0.54 : 0.46	0.56 : 0.44
RM20460	0.58 : 0.42	0.54 : 0.46	**0.59 : 0.41**	**0.52 : 0.48**
RM5371	0.61 : 0.39	0.58 : 0.42	**0.63 : 0.37**	**0.55 : 0.45**
RM16071	0.56 : 0.44	0.51 : 0.49	0.54 : 0.46	0.56 : 0.44
Average	0.51 : 0.49	0.47 : 0.53	0.50 : 0.50	0.52 : 0.48

Primer name	F_2 ideal-1	F_2 ideal-2	F_3 ideal-1	F_3 ideal-2
RM84	0.38 : 0.62	0.41 : 0.59	0.36 : 0.64	0.37 : 0.63
RM575	0.41 : 0.59	0.41 : 0.59	0.49 : 0.51	0.46 : 0.54
RM572	0.48 : 0.52	0.46 : 0.54	0.53 : 0.47	0.53 : 0.47

RM9	0.48 : 0.52	0.49 : 0.51	0.45 : 0.55	0.43 : 0.57
RM5	0.49 : 0.51	0.49 : 0.51	0.48 : 0.52	0.47 : 0.53
RM212	0.37 : 0.63	0.39 : 0.61	0.42 : 0.58	0.40 : 0.60
RM6895	0.52 : 0.48	0.53 : 0.47	0.66 : 0.34	0.64 : 0.36
RM7245	0.54 : 0.46	0.54 : 0.46	0.56 : 0.44	0.57 : 0.43
RM523	0.53 : 0.47	0.52 : 0.48	0.62 : 0.38	0.62 : 0.38
RM282	0.37 : 0.63	0.37 : 0.63	0.28 : 0.72	0.28 : 0.72
RM16	0.58 : 0.42	0.59 : 0.41	0.63 : 0.37	0.63 : 0.37
RM186	0.30 : 0.70	0.31 : 0.69	0.29 : 0.71	0.31 : 0.69
RM468	0.32 : 0.68	0.33 : 0.67	0.32 : 0.68	0.33 : 0.67
RM6395	0.51 : 0.49	0.52 : 0.48	0.36 : 0.64	0.37 : 0.63
RM6089	0.51 : 0.49	0.51 : 0.49	0.53 : 0.47	0.54 : 0.46
RM3558	0.50 : 0.50	0.50 : 0.50	0.43 : 0.57	0.42 : 0.58
RM6748	0.49 : 0.51	0.51 : 0.49	0.47 : 0.53	0.47 : 0.53
RM3419	0.56 : 0.44	0.55 : 0.45	0.55 : 0.45	0.54 : 0.46
RM6841	0.71 : 0.29	0.71 : 0.29	0.73 : 0.27	0.71 : 0.29
RM190	0.21 : 0.79	0.21 : 0.79	0.27 : 0.73	0.28 : 0.72
RM276	0.52 : 0.48	0.50 : 0.50	0.49 : 0.51	0.49 : 0.51
RM214	0.33 : 0.67	0.32 : 0.68	0.30 : 0.70	0.29 : 0.71
RM11	0.46 : 0.54	0.45 : 0.55	0.56 : 0.44	0.55 : 0.45
RM505	0.59 : 0.41	0.58 : 0.42	0.65 : 0.35	0.67 : 0.33
RM172	0.49 : 0.51	0.50 : 0.50	0.51 : 0.49	0.53 : 0.47
RM408	0.60 : 0.40	0.58 : 0.42	0.51 : 0.49	0.51 : 0.49
RM5068	0.17 : 0.83	0.16 : 0.84	0.22 : 0.78	0.23 : 0.77
RM331	0.59 : 0.41	0.61 : 0.39	0.62 : 0.38	0.61 : 0.39
RM296	0.57 : 0.43	0.55 : 0.45	0.60 : 0.40	0.59 : 0.41

RM4455	0.40 : 0.60	0.38 : 0.62	0.62 : 0.38	0.61 : 0.39
RM7557	0.43 : 0.57	0.43 : 0.57	0.46 : 0.54	0.45 : 0.55
RM332	0.48 : 0.52	0.47 : 0.53	0.60 : 0.40	0.59 : 0.41
RM4862	0.83 : 0.17	0.82 : 0.18	0.79 : 0.21	0.79 : 0.21
RM224	0.37 : 0.63	0.36 : 0.64	0.42 : 0.58	0.42 : 0.58
RM8216	0.54 : 0.46	0.54 : 0.46	0.67 : 0.33	0.66 : 0.34
RM3483	0.56 : 0.44	0.55 : 0.45	0.57 : 0.43	0.60 : 0.40
RM6296	0.54 : 0.46	0.55 : 0.45	0.67 : 0.33	0.68 : 0.32
RM277	0.52 : 0.48	0.52 : 0.48	0.55 : 0.45	0.53 : 0.47
RM6732	0.30 : 0.70	0.29 : 0.71	0.33 : 0.67	0.35 : 0.65
RM17	0.33 : 0.67	0.32 : 0.68	0.45 : 0.55	0.45 : 0.55
RM111	0.41 : 0.59	0.38 : 0.62	0.62 : 0.38	0.61 : 0.39
RM143	0.56 : 0.44	0.58 : 0.42	0.51 : 0.49	0.54 : 0.46
RM333	0.52 : 0.48	0.52 : 0.48	0.51 : 0.49	0.51 : 0.49
RM4128	0.53 : 0.47	0.52 : 0.48	0.44 : 0.56	0.43 : 0.57
RM168	0.32 : 0.68	0.33 : 0.67	0.33 : 0.67	0.34 : 0.66
RM565	0.34 : 0.66	0.37 : 0.63	0.62 : 0.38	0.61 : 0.39
RM16074	0.48 : 0.52	0.50 : 0.50	0.51 : 0.49	0.53 : 0.47
RM20468	0.50 : 0.50	0.50 : 0.50	0.57 : 0.43	0.57 : 0.43
RM130	0.49 : 0.51	0.51 : 0.49	0.54 : 0.46	0.55 : 0.45
RM20460	0.50 : 0.50	0.49 : 0.51	0.57 : 0.43	0.57 : 0.43
RM5371	0.56 : 0.44	0.55 : 0.45	0.61 : 0.39	0.60 : 0.40
RM16071	0.55 : 0.45	0.57 : 0.43	0.54 : 0.46	0.56 : 0.44
Average	0.47 : 0.53	0.47 : 0.53	0.50 : 0.50	0.50 : 0.50

Supplementary Table 6 Parent-allele frequencies (crop : weed) in F_2 and F_3 crop-weed hybrid lineages with (CM) or without (non-CM) the crop-parent markers; and with (WM) or without (non-WM) the weedy-parent markers, using the ideal groups as a reference. Frequencies in bold indicate significant differences (P <0.05) between lineages.

Primer name	F_2 CM-RM3533	F_2 non-CM-RM3533	F_2 WM-RM3533	F_2 non-WM-RM3533
RM84	**0.68 : 0.32**	**0.59 : 0.41**	**0.62 : 0.38**	**0.74 : 0.26**
RM283	**0.60 : 0.40**	**0.50 : 0.50**	**0.54 : 0.46**	**0.66 : 0.34**
RM23	0.51 : 0.49	0.47 : 0.53	**0.45 : 0.55**	**0.61 : 0.39**
RM5	**0.64 : 0.36**	**0.57 : 0.43**	**0.57 : 0.43**	**0.77 : 0.23**
RM128	**0.67 : 0.33**	**0.61 : 0.39**	**0.59 : 0.41**	**0.83 : 0.17**
RM5356	**0.53 : 0.47**	**0.44 : 0.56**	0.51 : 0.49	0.49 : 0.51
RM5390	**0.50 : 0.50**	**0.40 : 0.60**	**0.45 : 0.55**	**0.52 : 0.48**
RM530	**0.43 : 0.57**	**0.52 : 0.48**	0.46 : 0.54	0.44 : 0.56
RM231	0.74 : 0.26	0.74 : 0.26	0.74 : 0.26	0.72 : 0.28
RM16	0.63 : 0.37	0.60 : 0.40	0.61 : 0.39	0.66 : 0.34
RM8277	0.55 : 0.45	0.58 : 0.42	0.56 : 0.44	0.55 : 0.45
RM168	**0.58 : 0.42**	**0.50 : 0.50**	**0.53 : 0.47**	**0.63 : 0.37**
RM186	0.54 : 0.46	0.57 : 0.43	0.54 : 0.46	0.59 : 0.41
RM520	0.84 : 0.16	0.83 : 0.17	**0.82 : 0.18**	**0.89 : 0.11**
RM532	**0.23 : 0.77**	**0.30 : 0.70**	**0.23 : 0.77**	**0.33 : 0.67**
RM211	0.50 : 0.50	0.50 : 0.50	**0.48 : 0.52**	**0.58 : 0.42**
RM190	0.54 : 0.46	0.50 : 0.50	**0.48 : 0.52**	**0.66 : 0.34**
RM587	0.61 : 0.39	0.58 : 0.42	**0.57 : 0.43**	**0.67 : 0.33**
RM4128	**0.68 : 0.32**	**0.78 : 0.22**	0.71 : 0.29	0.73 : 0.27
RM527	0.53 : 0.47	0.50 : 0.50	0.52 : 0.48	0.52 : 0.48
RM340	0.49 : 0.51	0.47 : 0.53	**0.44 : 0.56**	**0.61 : 0.39**
RM295	0.52 : 0.48	0.50 : 0.50	**0.46 : 0.54**	**0.66 : 0.34**

RM214	**0.45 : 0.55**	**0.39 : 0.61**	**0.38 : 0.62**	**0.60 : 0.40**
RM172	0.55 : 0.45	0.57 : 0.43	**0.59 : 0.41**	**0.46 : 0.54**
RM8243	0.56 : 0.44	0.56 : 0.44	0.56 : 0.44	0.58 : 0.42
RM447	**0.71 : 0.29**	**0.01 : 0.99**	**0.33 : 0.67**	**0.99 : 0.01**
RM8206	**0.58 : 0.42**	**0.52 : 0.48**	**0.52 : 0.48**	**0.67 : 0.33**
RM296	**0.53 : 0.47**	**0.33 : 0.67**	**0.40 : 0.60**	**0.65 : 0.35**
RM4862	0.71 : 0.29	0.64 : 0.36	**0.66 : 0.34**	**0.78 : 0.22**
RM3117	**0.84 : 0.16**	**0.75 : 0.25**	0.80 : 0.20	0.85 : 0.15
RM7102	0.54 : 0.46	0.51 : 0.49	**0.55 : 0.45**	**0.44 : 0.56**
RM3533	-	-	-	-
Average	0.58 : 0.42	0.53 : 0.47	0.54 : 0.46	0.64 : 0.36

Primer name	F_3 CM-RM3533	F_3 non-CM-RM3533	F_3 WM-RM3533	F_3 non-WM-RM3533
RM84	**0.67 : 0.33**	**0.50 : 0.50**	**0.56 : 0.44**	**0.69 : 0.31**
RM283	**0.68 : 0.32**	**0.50 : 0.50**	**0.56 : 0.44**	**0.70 : 0.30**
RM23	**0.67 : 0.33**	**0.46 : 0.54**	**0.55 : 0.45**	**0.66 : 0.34**
RM5	0.65 : 0.35	0.60 : 0.40	**0.61 : 0.39**	**0.67 : 0.33**
RM128	0.71 : 0.29	0.66 : 0.34	**0.67 : 0.33**	**0.75 : 0.25**
RM5356	**0.46 : 0.54**	**0.55 : 0.45**	**0.52 : 0.48**	**0.46 : 0.54**
RM5390	**0.50 : 0.50**	**0.40 : 0.60**	**0.44 : 0.56**	**0.50 : 0.50**
RM530	0.51 : 0.49	0.44 : 0.56	**0.46 : 0.54**	**0.54 : 0.46**
RM231	0.59 : 0.41	0.63 : 0.37	**0.64 : 0.36**	**0.56 : 0.44**
RM16	**0.57 : 0.43**	**0.47 : 0.53**	**0.51 : 0.49**	**0.57 : 0.43**
RM8277	0.52 : 0.48	0.54 : 0.46	**0.55 : 0.45**	**0.49 : 0.51**
RM168	0.43 : 0.57	0.48 : 0.52	0.46 : 0.54	0.45 : 0.55
RM186	0.48 : 0.52	0.51 : 0.49	**0.52 : 0.48**	**0.45 : 0.55**

RM520	**0.88 : 0.12**	**0.82 : 0.18**	**0.83 : 0.17**	**0.90 : 0.10**
RM532	0.43 : 0.57	0.39 : 0.61	0.41 : 0.59	0.43 : 0.57
RM211	0.48 : 0.52	0.52 : 0.48	0.49 : 0.51	0.50 : 0.50
RM190	**0.60 : 0.40**	**0.52 : 0.48**	**0.52 : 0.48**	**0.66 : 0.34**
RM587	0.60 : 0.40	0.59 : 0.41	**0.57 : 0.43**	**0.63 : 0.37**
RM4128	0.47 : 0.53	0.43 : 0.57	0.45 : 0.55	0.47 : 0.53
RM527	0.59 : 0.41	0.60 : 0.40	0.60 : 0.40	0.58 : 0.42
RM340	**0.50 : 0.50**	**0.39 : 0.61**	**0.41 : 0.59**	**0.53 : 0.47**
RM295	**0.53 : 0.47**	**0.45 : 0.55**	0.51 : 0.49	0.46 : 0.54
RM214	**0.56 : 0.44**	**0.46 : 0.54**	**0.45 : 0.55**	**0.66 : 0.34**
RM172	0.54 : 0.46	0.50 : 0.50	0.53 : 0.47	0.50 : 0.50
RM8243	0.54 : 0.46	0.54 : 0.46	0.56 : 0.44	0.51 : 0.49
RM447	**0.55 : 0.45**	**0.46 : 0.54**	**0.49 : 0.51**	**0.55 : 0.45**
RM8206	**0.41 : 0.59**	**0.50 : 0.50**	**0.49 : 0.51**	**0.36 : 0.64**
RM296	**0.49 : 0.51**	**0.29 : 0.71**	**0.34 : 0.66**	**0.54 : 0.46**
RM4862	0.49 : 0.51	0.45 : 0.55	**0.45 : 0.55**	**0.51 : 0.49**
RM3117	0.81 : 0.19	0.80 : 0.20	**0.78 : 0.22**	**0.86 : 0.14**
RM7102	0.52 : 0.48	0.50 : 0.50	0.52 : 0.48	0.49 : 0.51
RM3533	**-**	**-**	-	-
Average	0.56 : 0.44	0.51 : 0.49	0.53 : 0.47	0.57 : 0.43

Primer name	F_2 CM-RM172	F_2 non-CM-RM172	F_2 WM-RM172	F_2 non-WM-RM172
RM84	0.66 : 0.34	0.62 : 0.38	0.64 : 0.36	0.69 : 0.31
RM283	**0.59 : 0.41**	**0.46 : 0.54**	**0.52 : 0.48**	**0.67 : 0.33**
RM23	**0.48 : 0.52**	**0.58 : 0.42**	0.52 : 0.48	0.47 : 0.53
RM5	**0.64 : 0.36**	**0.56 : 0.44**	0.62 : 0.38	0.62 : 0.38

RM128	**0.68 : 0.32**	**0.54 : 0.46**	**0.61 : 0.39**	**0.74 : 0.26**
RM5356	**0.54 : 0.46**	**0.41 : 0.59**	0.49 : 0.51	0.55 : 0.45
RM5390	0.46 : 0.54	0.51 : 0.49	**0.49 : 0.51**	**0.42 : 0.58**
RM530	0.46 : 0.54	0.44 : 0.56	**0.44 : 0.56**	**0.50 : 0.50**
RM231	**0.77 : 0.23**	**0.58 : 0.42**	**0.71 : 0.29**	**0.80 : 0.20**
RM16	0.62 : 0.38	0.63 : 0.37	0.61 : 0.39	0.66 : 0.34
RM8277	**0.58 : 0.42**	**0.46 : 0.54**	**0.54 : 0.46**	**0.61 : 0.39**
RM168	**0.57 : 0.43**	**0.47 : 0.53**	0.55 : 0.45	0.57 : 0.43
RM186	**0.58 : 0.42**	**0.45 : 0.55**	**0.53 : 0.47**	**0.60 : 0.40**
RM520	**0.88 : 0.12**	**0.76 : 0.24**	**0.82 : 0.18**	**0.94 : 0.06**
RM532	**0.29 : 0.71**	**0.16 : 0.84**	0.23 : 0.77	0.28 : 0.72
RM211	0.50: 0.50	0.52 : 0.48	0.50 : 0.50	0.50 : 0.50
RM190	**0.57 : 0.43**	**0.44 : 0.56**	0.53 : 0.47	0.53 : 0.47
RM587	0.61 : 0.39	0.56 : 0.44	**0.62 : 0.38**	**0.55 : 0.45**
RM4128	**0.73 : 0.27**	**0.65 : 0.35**	**0.74 : 0.26**	**0.65 : 0.35**
RM527	0.53 : 0.47	0.51 : 0.49	0.51 : 0.49	0.56 : 0.44
RM340	**0.47 : 0.53**	**0.56 : 0.44**	**0.47 : 0.53**	**0.53 : 0.47**
RM295	**0.54 : 0.46**	**0.41 : 0.59**	0.52 : 0.48	0.52 : 0.48
RM214	0.43 : 0.57	0.44 : 0.56	0.43 : 0.57	0.43 : 0.57
RM172	-	-	-	-
RM8243	**0.55 : 0.45**	**0.61 : 0.39**	0.57 : 0.43	0.54 : 0.46
RM447	0.54 : 0.46	0.54 : 0.46	**0.50 : 0.50**	**0.64 : 0.36**
RM8206	**0.61 : 0.39**	**0.33 : 0.67**	**0.53 : 0.47**	**0.63 : 0.37**
RM296	**0.49 : 0.51**	**0.34 : 0.66**	**0.44 : 0.56**	**0.52 : 0.48**
RM4862	**0.70 : 0.30**	**0.64 : 0.36**	**0.75 : 0.25**	**0.56 : 0.44**
RM3117	**0.80 : 0.20**	**0.88 : 0.12**	0.82 : 0.18	0.79 : 0.21

RM7102	0.53 : 0.47	0.52 : 0.48	0.53 : 0.47	0.51 : 0.49
RM3533	0.53 : 0.47	0.54 : 0.46	**0.50 : 0.50**	**0.63 : 0.37**
Average	0.58 : 0.42	0.52 : 0.48	0.56 : 0.44	0.59 : 0.41

Primer name	F_3 CM-RM172	F_3 non-CM-RM172	F_3 WM-RM172	F_3 non-WM-RM172
RM84	**0.64 : 0.36**	**0.54 : 0.46**	**0.58 : 0.42**	**0.63 : 0.37**
RM283	**0.65 : 0.35**	**0.54 : 0.46**	**0.56 : 0.44**	**0.68 : 0.32**
RM23	**0.62 : 0.38**	**0.54 : 0.46**	**0.56 : 0.44**	**0.62 : 0.38**
RM5	0.64 : 0.36	0.61 : 0.39	**0.61 : 0.39**	**0.66 : 0.34**
RM128	**0.72 : 0.28**	**0.65 : 0.35**	**0.67 : 0.33**	**0.73 : 0.27**
RM5356	**0.51 : 0.49**	**0.46 : 0.54**	**0.46 : 0.54**	**0.54 : 0.46**
RM5390	0.45 : 0.55	0.49 : 0.51	0.49 : 0.51	0.42 : 0.58
RM530	0.50 : 0.50	0.46 : 0.54	0.48 : 0.52	0.49 : 0.51
RM231	**0.65 : 0.35**	**0.53 : 0.47**	**0.53 : 0.47**	**0.72 : 0.28**
RM16	0.52 : 0.48	0.55 : 0.45	0.54 : 0.46	0.52 : 0.48
RM8277	0.53 : 0.47	0.52 : 0.48	0.54 : 0.46	0.51 : 0.49
RM168	0.46 : 0.54	0.45 : 0.55	**0.41 : 0.59**	**0.52 : 0.48**
RM186	0.50 : 0.50	0.48 : 0.52	0.50 : 0.50	0.48 : 0.52
RM520	**0.83 : 0.17**	**0.90 : 0.10**	**0.91 : 0.09**	**0.77 : 0.23**
RM532	0.41 : 0.59	0.42 : 0.58	**0.44 : 0.56**	**0.37 : 0.63**
RM211	0.50 : 0.50	0.49 : 0.51	0.49 : 0.51	0.50 : 0.50
RM190	0.54 : 0.46	0.61 : 0.39	**0.62 : 0.38**	**0.50 : 0.50**
RM587	**0.57 : 0.43**	**0.63 : 0.37**	**0.64 : 0.36**	**0.53 : 0.47**
RM4128	0.47 : 0.53	0.43 : 0.57	**0.43 : 0.57**	**0.49 : 0.51**
RM527	**0.57 : 0.43**	**0.63 : 0.37**	**0.62 : 0.38**	**0.56 : 0.44**
RM340	0.44 : 0.56	0.48 : 0.52	0.44 : 0.56	0.48 : 0.52

RM295	**0.50 : 0.50**	**0.50 : 0.50**	0.50 : 0.50	0.49 : 0.51
RM214	0.51 : 0.49	0.54 : 0.46	0.52 : 0.48	0.52 : 0.48
RM172	-	-	-	-
RM8243	0.54 : 0.46	0.54 : 0.46	0.54 : 0.46	0.54 : 0.46
RM447	0.51 : 0.49	0.53 : 0.47	0.53 : 0.47	0.50 : 0.50
RM8206	**0.40 : 0.60**	**0.54 : 0.46**	0.47 : 0.53	0.41 : 0.59
RM296	**0.45 : 0.55**	**0.34 : 0.66**	**0.39 : 0.61**	**0.45 : 0.55**
RM4862	0.47 : 0.53	0.49 : 0.51	0.50 : 0.50	0.44 : 0.56
RM3117	0.80 : 0.20	0.83 : 0.17	**0.83 : 0.17**	**0.77 : 0.23**
RM7102	0.49 : 0.51	0.55 : 0.45	**0.55 : 0.45**	**0.46 : 0.54**
RM3533	0.49 : 0.51	0.46 : 0.54	0.49 : 0.51	0.47 : 0.53
Average	0.55 : 0.45	0.54 : 0.46	0.50 : 0.50	0.54 : 0.46

Primer name	F_2 CM-RM186	F_2 non-CM-RM186	F_2 WM-RM186	F_2 non-WM-RM186
RM84	**0.68 : 0.32**	**0.53 : 0.47**	0.65 : 0.35	0.67 : 0.33
RM283	**0.59 : 0.41**	**0.47 : 0.53**	0.56 : 0.44	0.58 : 0.42
RM23	**0.48 : 0.52**	**0.60 : 0.40**	**0.53 : 0.47**	**0.44 : 0.56**
RM5	**0.63 : 0.37**	**0.56 : 0.44**	**0.58 : 0.42**	**0.71 : 0.29**
RM128	0.65 : 0.35	0.64 : 0.36	0.64 : 0.36	0.69 : 0.31
RM5356	**0.53 : 0.47**	**0.38 : 0.62**	0.50 : 0.50	0.51 : 0.49
RM5390	**0.49 : 0.51**	**0.37 : 0.63**	0.47 : 0.53	0.47 : 0.53
RM530	**0.48 : 0.52**	**0.33 : 0.67**	**0.41 : 0.59**	**0.57 : 0.43**
RM231	**0.76 : 0.24**	**0.64 : 0.36**	**0.72 : 0.28**	**0.78 : 0.22**
RM16	**0.70 : 0.30**	**0.27 : 0.73**	**0.55 : 0.45**	**0.82 : 0.18**
RM8277	**0.66 : 0.34**	**0.09 : 0.91**	**0.41 : 0.59**	**0.94 : 0.06**
RM168	**0.65 : 0.35**	**0.10 : 0.90**	**0.39 : 0.61**	**0.96 : 0.04**

RM186	-	-	-	-
RM520	0.85 : 0.15	0.82 : 0.18	0.84 : 0.16	0.84 : 0.16
RM532	**0.32 : 0.68**	**0.02 : 0.98**	**0.05 : 0.95**	**0.89 : 0.11**
RM211	**0.52 : 0.48**	**0.44 : 0.56**	**0.52 : 0.48**	**0.46 : 0.54**
RM190	**0.52 : 0.48**	**0.58 : 0.42**	**0.51 : 0.49**	**0.59 : 0.41**
RM587	**0.58 : 0.42**	**0.68 : 0.32**	0.58 : 0.42	0.63 : 0.37
RM4128	**0.75 : 0.25**	**0.52 : 0.48**	0.70 : 0.30	0.74 : 0.26
RM527	**0.51 : 0.49**	**0.58 : 0.42**	**0.54 : 0.46**	**0.48 : 0.52**
RM340	**0.52 : 0.48**	**0.29 : 0.71**	0.48 : 0.52	0.49 : 0.51
RM295	**0.53 : 0.47**	**0.45 : 0.55**	**0.55 : 0.45**	**0.44 : 0.56**
RM214	**0.44 : 0.56**	**0.38 : 0.62**	0.43 : 0.57	0.45 : 0.55
RM172	0.56 : 0.44	0.53 : 0.47	**0.52 : 0.48**	**0.66 : 0.34**
RM8243	**0.58 : 0.42**	**0.45 : 0.55**	0.55 : 0.45	0.58 : 0.42
RM447	0.53 : 0.47	0.57 : 0.43	**0.52 : 0.48**	**0.64 : 0.36**
RM8206	0.57 : 0.43	0.53 : 0.47	0.57 : 0.43	0.53 : 0.47
RM296	**0.48 : 0.52**	**0.37 : 0.63**	0.46 : 0.54	0.48 : 0.52
RM4862	**0.72 : 0.28**	**0.54 : 0.46**	**0.67 : 0.33**	**0.74 : 0.26**
RM3117	0.80 : 0.20	0.84 : 0.16	**0.79 : 0.21**	**0.85 : 0.15**
RM7102	0.52 : 0.48	0.54 : 0.46	**0.56 : 0.44**	**0.43 : 0.57**
RM3533	**0.52 : 0.48**	**0.63 : 0.37**	**0.52 : 0.48**	**0.58 : 0.42**
Average	0.58 : 0.42	0.48 : 0.52	0.54 : 0.46	0.63 : 0.37

Primer name	F_3 CM-RM186	F_3 non-CM-RM186	F_3 WM-RM186	F_3 non-WM-RM186
RM84	0.61 : 0.39	0.59 : 0.41	0.59 : 0.41	0.62 : 0.38
RM283	**0.58 : 0.42**	**0.66 : 0.34**	**0.64 : 0.36**	**0.56 : 0.44**
RM23	0.57 : 0.43	0.61 : 0.39	0.58 : 0.42	0.61 : 0.39

RM5	**0.61 : 0.39**	**0.67 : 0.33**	**0.60 : 0.40**	**0.68 : 0.32**
RM128	**0.65 : 0.35**	**0.76 : 0.24**	0.69 : 0.31	0.69 : 0.31
RM5356	**0.45 : 0.55**	**0.56 : 0.44**	0.49 : 0.51	0.50 : 0.50
RM5390	0.46 : 0.54	0.46 : 0.54	0.46 : 0.54	0.45 : 0.55
RM530	0.50 : 0.50	0.46 : 0.54	0.47 : 0.53	0.52 : 0.48
RM231	0.61 : 0.39	0.60 : 0.40	0.59 : 0.41	0.64 : 0.36
RM16	**0.62 : 0.38**	**0.38 : 0.62**	**0.46 : 0.54**	**0.66 : 0.34**
RM8277	**0.78 : 0.22**	**0.11 : 0.89**	**0.27 : 0.73**	**0.98 : 0.02**
RM168	**0.70 : 0.30**	**0.03 : 0.97**	**0.25 : 0.75**	**0.79 : 0.21**
RM186	-	-	-	-
RM520	**0.91 : 0.09**	**0.77 : 0.23**	**0.83 : 0.17**	**0.91 : 0.09**
RM532	**0.57 : 0.43**	**0.16 : 0.84**	**0.15 : 0.85**	**0.87 : 0.13**
RM211	**0.52 : 0.48**	**0.46 : 0.54**	0.51 : 0.49	0.47 : 0.53
RM190	0.55 : 0.45	0.59 : 0.41	0.56 : 0.44	0.57 : 0.43
RM587	0.58 : 0.42	0.61 : 0.39	0.58 : 0.42	0.61 : 0.39
RM4128	**0.53 : 0.47**	**0.34 : 0.66**	**0.39 : 0.61**	**0.57 : 0.43**
RM527	**0.55 : 0.45**	**0.67 : 0.33**	**0.63 : 0.37**	**0.52 : 0.48**
RM340	**0.51 : 0.49**	**0.37 : 0.63**	0.45 : 0.55	0.46 : 0.54
RM295	**0.54 : 0.46**	**0.43 : 0.57**	**0.41 : 0.59**	**0.64 : 0.36**
RM214	**0.47 : 0.53**	**0.61 : 0.39**	**0.54 : 0.46**	**0.48 : 0.52**
RM172	0.51 : 0.49	0.54 : 0.46	0.51 : 0.49	0.54 : 0.46
RM8243	**0.59 : 0.41**	**0.46 : 0.54**	**0.50 : 0.50**	**0.60 : 0.40**
RM447	**0.54 : 0.46**	**0.48 : 0.52**	**0.47 : 0.53**	**0.60 : 0.40**
RM8206	**0.47 : 0.53**	**0.41 : 0.59**	0.46 : 0.54	0.43 : 0.57
RM296	0.42 : 0.58	0.41 : 0.59	0.40 : 0.60	0.44 : 0.56
RM4862	0.49 : 0.51	0.44 : 0.56	0.46 : 0.54	0.50 : 0.50

RM3117	0.81 : 0.19	0.81 : 0.19	0.81 : 0.19	0.81 : 0.19
RM7102	0.51 : 0.49	0.51 : 0.49	0.51 : 0.49	0.52 : 0.48
RM3533	0.46 : 0.54	0.50 : 0.50	**0.50 : 0.50**	**0.44 : 0.56**
Average	0.57 : 0.43	0.50 : 0.50	0.51 : 0.49	0.60 : 0.40

Primer name	F_2 ideal-1	F_2 ideal-2	F_3 ideal-1	F_3 ideal-2
RM84	0.65 : 0.35	0.65 : 0.35	0.61 : 0.39	0.60 : 0.40
RM283	0.57 : 0.43	0.56 : 0.44	0.35 : 0.65	0.35 : 0.65
RM23	0.51 : 0.49	0.50 : 0.50	0.60 : 0.40	0.58 : 0.42
RM5	0.62 : 0.38	0.63 : 0.37	0.64 : 0.36	0.62 : 0.38
RM128	0.65 : 0.35	0.65 : 0.35	0.70 : 0.30	0.69 : 0.31
RM5356	0.51 : 0.49	0.50 : 0.50	0.43 : 0.57	0.44 : 0.56
RM5390	0.47 : 0.53	0.47 : 0.53	0.42 : 0.58	0.43 : 0.57
RM530	0.46 : 0.54	0.46 : 0.54	0.49 : 0.51	0.48 : 0.52
RM231	0.73 : 0.27	0.74 : 0.26	0.60 : 0.40	0.60 : 0.40
RM16	0.62 : 0.38	0.64 : 0.36	0.53 : 0.47	0.54 : 0.46
RM8277	0.56 : 0.44	0.57 : 0.43	0.53 : 0.47	0.53 : 0.47
RM168	0.55 : 0.45	0.56 : 0.44	0.45 : 0.55	0.45 : 0.55
RM186	0.55 : 0.45	0.56 : 0.44	0.49 : 0.51	0.50 : 0.50
RM520	0.84 : 0.16	0.83 : 0.17	0.86 : 0.14	0.86 : 0.14
RM532	0.25 : 0.75	0.26 : 0.74	0.42 : 0.58	0.41 : 0.59
RM211	0.51 : 0.49	0.49 : 0.51	0.50 : 0.50	0.48 : 0.52
RM190	0.53 : 0.47	0.52 : 0.48	0.51 : 0.49	0.53 : 0.47
RM587	0.60 : 0.40	0.59 : 0.41	0.60 : 0.40	0.59 : 0.41
RM4128	0.71 : 0.29	0.72 : 0.28	0.45 : 0.55	0.47 : 0.53
RM527	0.52 : 0.48	0.53 : 0.47	0.60 : 0.40	0.58 : 0.42

RM340	0.49 : 0.51	0.48 : 0.52	0.46 : 0.54	0.46 : 0.54
RM295	0.52 : 0.48	0.53 : 0.47	0.50 : 0.50	0.49 : 0.51
RM214	0.43 : 0.57	0.43 : 0.57	0.53 : 0.47	0.52 : 0.48
RM172	0.56 : 0.44	0.56 : 0.44	0.52 : 0.48	0.53 : 0.47
RM8243	0.56 : 0.44	0.56 : 0.44	0.54 : 0.46	0.54 : 0.46
RM447	0.54 : 0.46	0.54 : 0.46	0.52 : 0.48	0.52 : 0.48
RM8206	0.56 : 0.44	0.56 : 0.44	0.45 : 0.55	0.45 : 0.55
RM296	0.47 : 0.53	0.46 : 0.54	0.41 : 0.59	0.41 : 0.59
RM4862	0.69 : 0.31	0.69 : 0.31	0.48 : 0.52	0.47 : 0.53
RM3117	0.81 : 0.19	0.82 : 0.18	0.81 : 0.19	0.80 : 0.20
RM7102	0.52 : 0.48	0.53 : 0.47	0.70 : 0.30	0.71 : 0.29
RM3533	0.54 : 0.46	0.54 : 0.46	0.48 : 0.52	0.47 : 0.53
Average	0.57 : 0.43	0.57 : 0.43	0.54 : 0.46	0.53 : 0.47

Supplementary Table 7 DNA sequences of SSR (single sequence repeat) primer pairs used in this study**.**

Primer name	Chr.	Forward primer	Reverse primer
RM84	1	taagggtccatccacaagatg	ttgcaaatgcagctagagtac
RM575	1	caatttccataggctgcatg	gcttgggttagcgacgac
RM283	1	gtctacatgtacccttgttggg	cggcatgagagtctgtgatg
RM572	1	cggttaatgtcatctgattgg	ttcgagatccaagactgacc
RM23	1	cattggagtggaggctgg	gtcaggcttctgccattctc
RM9	1	ggtgccattgtcgtcctc	acggccctcatcaccttc
RM5	1	tgcaacttctagctgctcga	gcatccgatcttgatggg
RM128	1	agcttgggtgatttcttggaagcg	acgacgaggagtcgccgtgcag
RM212	1	ccactttcagctactaccag	cacccatttgtctctcattatg
RM6895	2	tcaaataagatggacggtc	cgccaccacagtagtactag
RM5356	2	agagcacttggagtgaagagg	aagcgagggagacagatgc
RM5390	2	gcaatttaacccttattcctg	gggaagaagaaagccattag
RM7245	2	ctcgatctggtgacatgacg	gaagaagtacgcgcccaac
RM530	2	gcactgaccacgactgtttg	accgtaacccggatctatcc
RM1251	2	gagacaatgacagtctgcgc	ccttcagcccttcacgtatc
RM523	3	aaggcattgcagctagaagc	gcacttgggaggtttgctag
RM231	3	ccagattatttcctgaggtc	cacttgcatagttctgcattg
RM282	3	ctgtgtcgaaaggctgcac	cagtcctgtgttgcagcaag
RM16	3	cgctagggcagcatctaaa	aacacagcaggtacgcgc
RM8277	3	agcacaagtaggtgcatttc	atttgcctgtgatgtaatagc
RM168	3	tgctgcttgcctgcttccttt	gaaacgaatcaatccacggc
RM186	3	tcctccatctcctccgctcccg	gggcgtggtggccttcttcgtc
RM520	3	aggagcaagaaaagttcccc	gccaatgtgtgacgcaatag
RM468	3	cccttccttgttgtggctac	tgatttctgagagccaaccc
RM143	3	gtcccgaaccctagcccgaggg	agaggccctccacatggcgacc
RM130	3	cgagaagaaggagcccatctcg	gcgaagcgaagaggagcaagg
RM16071	3	ttcgatgaacgatctggtttgc	gctttggtagccaaccttgtacg
RM16074	3	gacgaccaccaagaaagtgatcg	acagcccgtcgatgaagtttagc
RM565	3	agtaacgagcatagcaggcg	gcaaagccttcaggaatcag
RM3558	4	acgagagatcttctttgcag	cctctatttatgcctctacgc
RM5320	4	tctataatgtagcccccccc	tttcaggggcttctaccaac
RM6089	4	ccaccgaatcgaataaccac	atggccagcgtgatctcc
RM6748	4	attgggtttctcatattatg	ccaacactcctaactagttc
RM3419	5	atcttggtgaaacagtgctc	ctgctgctattcctcaagac
RM6841	5	ggcccacatgtcagttacac	cccaccagcctcacttactg
RM211	5	ccgatctcatcaaccaactg	cttcacgaggatctcaaagg
RM190	6	ctttgtctatctcaagacac	ttgcagatgttcttcctgatg
RM587	6	acgcgaacaaattaacagcc	ctttgctaccagtagatccagc
RM111	6	cacaacctttgagcaccgggtc	acgcctgcagcttgatcaccgg
RM276	6	ctcaacgttgacacctcgtg	tcctccatcgagcagtatca
RM4128	6	agtaactcgatcaaactaac	agagtccatatagaatttca

RM527	6	ggctcgatctagaaaatccg	ttgcacaggttgcgatagag
RM5371	6	gcagaggatgcccacttaattcc	gggctagctttagctgcgttgc
RM20460	6	acatcccactcccaatcttgtgc	ctctcgctgctgcttacgattcc
RM20468	6	aaacgagaatccggccatcttcg	gaagccgacgacctgatgttcc
RM6395	6	ggcttcggcttctgaactagc	cgactaagcagcagtaacaatctcg
RM340	6	gggtaaatggacaatcctatgg	accctattctggagttcatctgg
RM295	7	cgagacgagcatcggataag	gatctggtggaggggagg
RM214	7	ctgatgatagaaacctcttctc	aagaacagctgacttcacaa
RM11	7	tctcctcttcccccgatc	atagcgggcgaggcttag
RM505	7	agagttatgagccgggtgtg	gatttggcgatcttagcagc
RM172	7	tgcagctgcgccacagccatag	caaccacgacaccgccgtgttg
RM408	8	caacgagctaacttccgtcc	actgctacttgggtagctgacc
RM5068	8	gaggtgtttatagaagtagg	aattagcttatcttgtgttc
RM8243	8	ctcgtgcaaccattatattc	accttagctgtcctgaattg
RM331	8	gaaccagaggacaaaaatgc	catcatacatttgcagccag
RM447	8	cccttgtgctgtctcctctc	acgggcttcttctccttctc
RM8206	9	tctttctggatcattggatg	actctgccaaggataacaag
RM296	9	cacatggcaccaacctcc	gccaagtcattcactactctgg
RM4455	10	ctctcaaagaactaggactc	gagaaggtatgataaccaat
RM333	10	gtacgactacgagtgtcaccaa	gtcttcgcgatcactcgc
RM7557	11	gtgtactgccatgaaaggcc	gaagtgcctttgcaggagag
RM332	11	gcgaaggcgaaggtgaag	catgagtgatctcactcaccc
RM4862	11	caactttctggcataaacta	tggtgaaagatatttcagac
RM3117	11	gccatctctctctctctctc	ccttagctcatcaagcgagg
RM224	11	atcgatcgatcttcacgagg	tgctataaaaggcattcggg
RM8216	12	agttgattatccatcatgtgc	tgtaggccctatgaatctcc
RM3483	12	cctagctttcaggagcaag	cccacaatgagaaacagttg
RM6296	12	tcttgcctcgctagggttag	cccacgtttctcttgtcctc
RM7102	12	cggcttgagagcgtttttag	tacttggttactcgggtcgg
RM3533	12	ttccaacctgtcagggaatc	catttcccttccctctcctc
RM277	12	cggtcaaatcatcacctgac	caaggcttgcaagggaag
RM6732	12	aattttgaacacctcaaagg	ttttcagtgcatgtcttcg
RM17	12	tgccctgttattttcttctctc	ggtgatcctttcccatttca
